# Evolutionary divergence of the nuclear pore complex from fungi to metazoans

**DOI:** 10.1002/pro.3558

**Published:** 2018-12-24

**Authors:** Kriti Chopra, Shrankhla Bawaria, Radha Chauhan

**Affiliations:** ^1^ National Center for Cell Science, S.P. Pune University Pune 411007 Maharashtra India

**Keywords:** nuclear pore complex, supertree, selection pressure, evolution

## Abstract

Nuclear pore complex (NPC) is the largest multimeric protein assembly of the eukaryotic cell, which mediates the nucleocytoplasmic transport. The constituent proteins of this assembly (nucleoporins) are present in varying copy numbers to give a size from ~ 60 MDa (yeast) to 112 MDa (human) and share common ancestry with other membrane‐associated complexes such as COPI/COPII and thus share the same structural folds. However, the nucleoporins across species exhibit very low percentage sequence similarity and this reflects in their distinct secondary structure and domain organization. We employed thorough sequence and phylogenetic analysis guided from structure‐based alignments of all the nucleoporins from fungi to metazoans to understand the evolution of NPC. Through evolutionary pressure analysis on various nucleoporins, we deduced that these proteins are under differential selection pressure and hence the homologous interacting partners do not complement each other in the *in vitro* pull‐down assay. The super tree analysis of all nucleoporins taken together illustrates divergent evolution of nucleoporins and notably, the degree of divergence is more apparent in higher order organisms as compared to lower species. Overall, our results support the hypothesis that the protein–protein interactions in such large multimeric assemblies are species specific in nature and hence their structure and function should also be studied in an organism‐specific manner.

**Abbreviations**
CTC: central transport channel
NPC: nuclear pore complex
Nup: nucleoporin
RMSD: root mean square deviation
hNup: *H. sapiens'* Nup
scNup: *S. cerevisiae* Nup
ctNup: *C. thermophilum* Nup
xNup: *X. laevis* Nup

## Introduction

The nuclear membrane is embedded with a multiprotein structure called the nuclear pore complex (NPC), which aids bidirectional transport of cargos. NPCs act as a selectivity barrier for the transport of cargos across the nuclear envelope and are permeable to small molecules such as ions and small metabolites.^1^ The tomographic studies of NPC depict it to be made up of three layers namely, cytoplasmic filaments, the spoke region which is anchored to the nuclear membrane and the nuclear basket towards the nucleoplasm, thus forming an hour‐glass shape architecture.^2^ These three regions are populated by distinct proteins called the nucleoporins (Nups) and are followed by a number which is their molecular weight.^3^, ^4^

The electron tomography‐based structures from *Xenopus* oocytes and later mammalian cells show that the NPC follows an eight‐fold rotational symmetry where each of the eight spokes consists of six sub‐complexes; hNup88‐hNup214‐hNup62 complex present on the cytoplasmic side, followed by two scaffold ring complexes annotated as the Y‐shaped complex (hNup133‐hNup107‐hNup85‐hNup160‐hNup96‐hSec13‐hSeh1‐hNup37‐hNup43‐hAladin‐hELYS) and adaptor ring complex (hNup93‐hNup155‐hNup35‐hNup205/hNup188).^5^, ^6^ The adaptor ring is anchored to the nuclear membrane with the help of transmembrane proteins (hPOM121‐hNDC1‐hGp210‐hTMEM33) and hNup93 of this subcomplex extends to interact with the central channel (hNup62‐hNup54‐hNup58) forming the pore of the NPC.^7^, ^8^ hNup153‐hNup50‐hTPR complex form the nuclear basket/ring of the mammalian NPC.^9^ The total mass of the intact NPC varies from about 60 MDa of yeast to 112 MDa for vertebrates.^10^, ^11^ This difference in size is marked by a number of factors such as a distinct number of Nups present in different species, the length of orthologous pairs of Nups, the oligomerization of Nups present in the various rings of the NPC, and post‐translational modifications of mammalian Nups.^12^, ^13^

Although the composition of NPC is believed to be taxonomically conserved, a number of proteomic‐based studies from various species such as *Aspergillus nidulans,* 14 *Schizosaccharomyces pombe,* 15 *Chaetomium thermophilum,* 16 *Arabidopsis thaliana,* 17 *Caenorhabditis elegans,* 18 *Trypanosoma brucei*,^19^ and *Tetrahymena thermophila* 20 report otherwise. Interestingly, all these species have differences in the number of Nups identified (Table S1). Overall, it is evident that there is significant variation in the number of Nups in different species as per the studies published till date.

The structural architecture of the individual nucleoporins is believed to indicate common ancestry with other eukaryotic endomembrane coatomer proteins such as those of COPI and COPII.^21^ Recently, Ancestral Coatomer Element I (ACE1) was defined as the common architectural motif across diverse coatomer molecules thus defining both sequence and structural conserved motifs between the vesicle transport and nuclear pore complexes.^22^ The fold analysis study of the *S. cerevisiae* Nups also shows that all the nucleoporins can be classified into seven classes which being α‐helical, β‐propeller, coiled coils, cadherin fold, RRM (RNA recognition motif), autoproteolytic fold (hNup98), and the unstructured FG repeat regions.^23^ These predicted fold types were assigned to nearly 28 Nups of *S. cerevisiae* which indicates that all of the Nups originated from a minimum set of precursor proteins by wide‐ranging intragenic and intergenic duplications.^23^

There has been enormous growth in the availability of tomography structures of the intact NPC from various sources such as *Xenopus* oocytes,^2^ *D. discodieum* nuclei^24^ and human carcinoma cell line,^25^ and the recent cryo‐electron tomography structure from *Xenopus* oocytes significantly improved the resolution to ~20 Å in the Y‐shaped region of the NPC and 50 Å–60 Å in the central channel region.^26^ Of the total number of X‐ray crystallography structures of Nups deposited in the PDB, there are only 12 non‐redundant partial domain structures from vertebrates sourced from *H. sapiens,* 27, 28, 29, 30, 31, 32, 33, 34, 35, 36, 37, 38, 39, 40 *M. musculus,* 41, 42, 43 *R. norvegius,* 44, 45, 46 and *X. leavis* 47 and four protein complex structures available from *H. sapiens,* 48, 49 *R. norvegius,* 50 and *X. leavis*.^47^ The scarcity in the crystal structures from metazoans origin is complemented by the presences of 11 non‐redundant partial domain structures from fungi sourced from *S. cerevisiae* 48, 51, 52, 53, 54, 55, 56, 57, 58, 59 and *C. thermophilum* 6, 60 along with 10 protein complex structures available from *S. cerevisiae* 61, 62, 63, 64, 65, 66, 67 and *C. thermophilum* 6, 60 (Table S2).

Recently, there have been reports whereby using the X‐ray crystal structures of either individual Nups or complexes from *C. thermophilum*, and the cryo‐ET maps of human NPC, the complete interaction network within the human NPC has been deciphered.^60^ The protein–protein interaction network of the NPC has been reported for both yeast and human through yeast two‐hybrid method^68^ but owing to its complexity, the picture is still unclear for the human NPC. Although the predicted folds seem to be conserved for all the species, there are obvious differences in the size of many orthologous Nup pairs which would lead to differences in their tertiary structure assemblies. Due to lack of availability of crystal structures of Nups from the mammalian origin, these aspects have not been studied well to date.

In this study, we aimed to identify the pattern of evolution of the nucleoporins from a structural perspective. Through in‐depth phylogenetic analysis, we report that the evolution of NPC is divergent in nature. By performing secondary structure and fold composition analysis of the metazoan (*H. sapiens*) nucleoporins in comparison to fungal species (*S. cerevisiae* and *C. thermophilum*), we were able to identify distinct structural features in some of the nucleoporins, which can also be explained in terms of evolution. We report that a majority of Nups of metazoan origin harbor differences at sequence/domain/secondary structure or tertiary structure level. Furthermore, our phylogenetic analysis across various species could identify the differences that certain Nups have domains specific to a group of species. The residue‐specific dN/dS analysis work out the codon substitution rates in the group of sequences provided and explain the probabilities of a codon/amino acid being under positive selection pressure(mutability) or purifying selection pressure (evolutionary conserved). This analysis gave us a clear insight into the presence of differential selection pressure on the sequences of Nups to harbor specific domains amongst different species. Our analysis sheds light on the regions of homologous Nups that are under positive selection pressure and which might lead to different interaction networks across species. These dissimilarities of specific nucleoporins from various species also indicate that NPC assembly could function in a species‐specific manner and is likely to be linked with the unique structural features of Nups of a particular species.

## Results and Discussion

### *Secondary structure prediction and domain analysis of all nucleoporins*

*S. cerevisiae* and *C. thermophilum* are two well‐studied fungal species with respect to nucleoporins, hence orthologue Nups of these two species were taken as query to identify the metazoan homologs (*H. sapiens*) through HMM‐based searches and validated through reciprocal searches. [Refer to Supplementary Material for details. HMM search results are listed in Tables S3(A–F) and UniProt Ids are listed in Table S4 (nomenclature translation table)]. Percentage sequence identity and similarity comparisons were made through both HMM searches as well as standard global alignments (Needleman–Wunsch algorithm) amongst the homologs of these fungal (*S. cerevisiae* and *C. thermophilum*) and metazoan (*H. sapiens*) species (Table S5). We could classify nucleoporins in two categories as those either showing moderate sequence conservation viz. Nup62, Nup54, Nup98, Nup155, Nup188, Nup205, Nup93, Sec13, TMEM33 and Rae1 (~20% sequence identity and ~30% sequence similarity) ,and few Nups (Nup35, Nup42, Nup214, Nup160, Nup58, Nup88, and Nup50) that have very low sequence conservation (less than 20% sequence identity).

**Figure 1:**
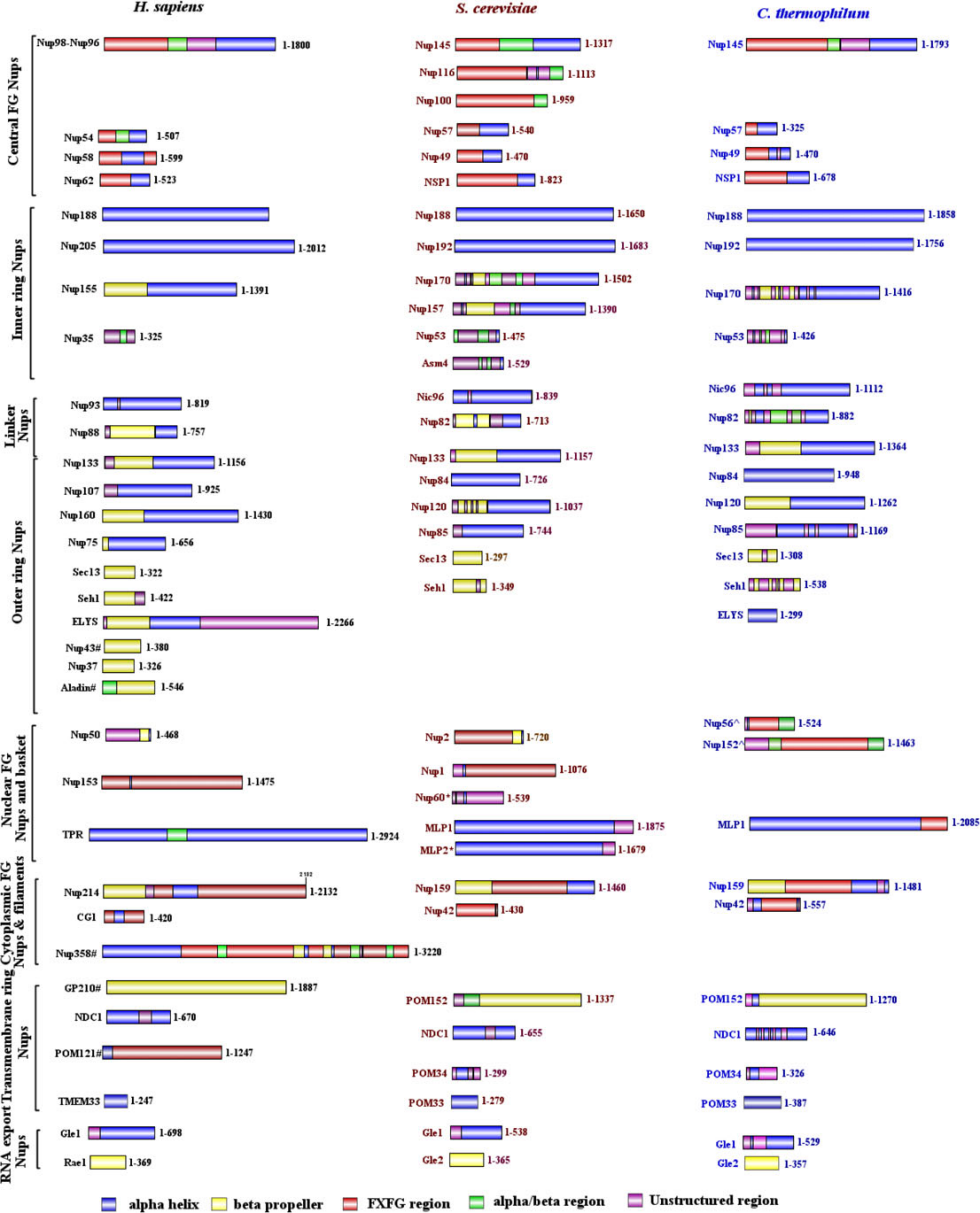
**Representative secondary structure organization for all the nucleoporins from *H. sapiens*, *S. cerevisiae,* and *C. thermophilum*.** PSIPRED was used to determine the secondary structure organization amongst the nucleoporins from three species. The α‐helix regions are shown in blue, β‐sheet regions in yellow, α/β‐regions in green, unstructured regions in purple and FG repeat regions in red. The image was generated using IBS illustrator.

**Figure 2:**
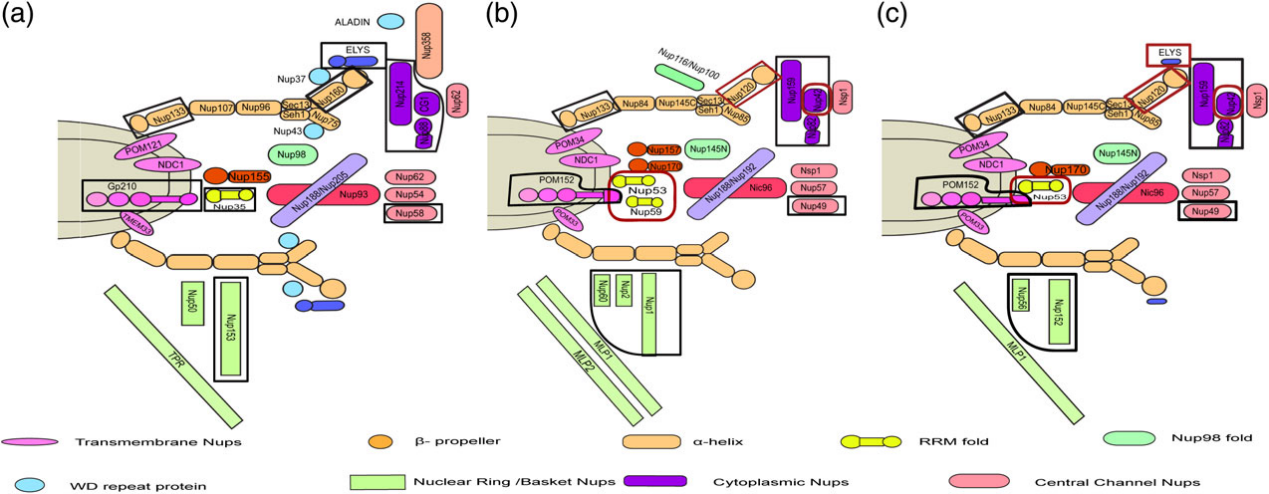
**Schematic representation of the compositional differences in human and yeast NPC.** Two‐dimensional representation of the NPC composition based on homology searches and fold prediction for (a) *H. sapiens*, (b) *S. cerevisiae,* and (c) *C. thermophilum*. These differences indicate the species‐specific composition of the nuclear pore complexes from different organisms. Differences between the metazoan and fungal species are marked with black lines and those between the fungal species are marked with a red line.

Despite low percentage sequence similarity, it may be argued that a structural conservation exists in the homologous nucleoporins owing to common coatomer ancestry. To decipher the similarity or differences, which might exist, we performed secondary structure predictions for all the Nups from three species (*H. sapiens*, *S. cerevisiae,* and *C. thermophilum*) using PSIPRED^69^ (Fig. 1). Along with secondary structure, the PFAM domains for each nucleoporin were also analyzed using HMMSCAN (Table S6). Notably, we observed some nucleoporins exhibit significant differences in the secondary structure and domain organization among the three species in spite of conservation at the sequence level (Fig. 2, S1). It is interesting to note that there are differences even between the two fungal species i.e. *C. thermophilum* and *S. cerevisiae* which are marked with * in Figure S1.

Comparing the composition of *H. sapiens* NPC with that of *C. thermophilum* [Figs. 2(a,c)], a number of differences can be highlighted. Apart from lacking the vertebrate‐specific Nups (Nup358, Nup37, Nup43, and Aladin), *C. thermophilum* does not have a true homolog of Nup50 and Nup153 which are the major components of the nuclear basket/ring.^70^, ^71^, ^72^ Instead, it has two additional Nups, Nup56 and Nup152, which have only the Ran‐binding domain. While comparing the trans‐membrane Nups from these two species, it was observed that the orientation in which Gp210 (C‐terminal)^73^ and POM152 (N‐terminal) anchor the nuclear membrane^74^ is distinct.

Interestingly, *S. cerevisiae* has many more components present in its NPC when compared to both *C. thermophilum* and *H. sapiens* [Figs. 2(a,b)]. First being the presence of paralogs for certain Nups such as hNup98 has three orthologs in budding yeast i.e. Nup145N, Nup116, and Nup100. Similarly, hNup155, hNup35, and hTPR have two orthologs each being Nup157/Nup170, Nup53/Nup59, and MLP1/MLP2 respectively. Apart from these, Nup60 present in the nuclear basket is unique to *S. cerevisiae* and its orthologs are absent in both *H. sapiens* and *C. thermophilum*.

It can be hypothesized that these sequence guided analysis and compositional differences would have implications on the overall assembly of the NPC in different species as well as protein–protein interactions of individual Nups across species. Details and implications of some of these differences apart from the ones mentioned above are discussed in detail in the following sections.

### *Distinct domain organization is present among species in ELYS and Nup133*

#### *A. Three classes of domain organization are predicted across species in ELYS*

**Figure 3:**
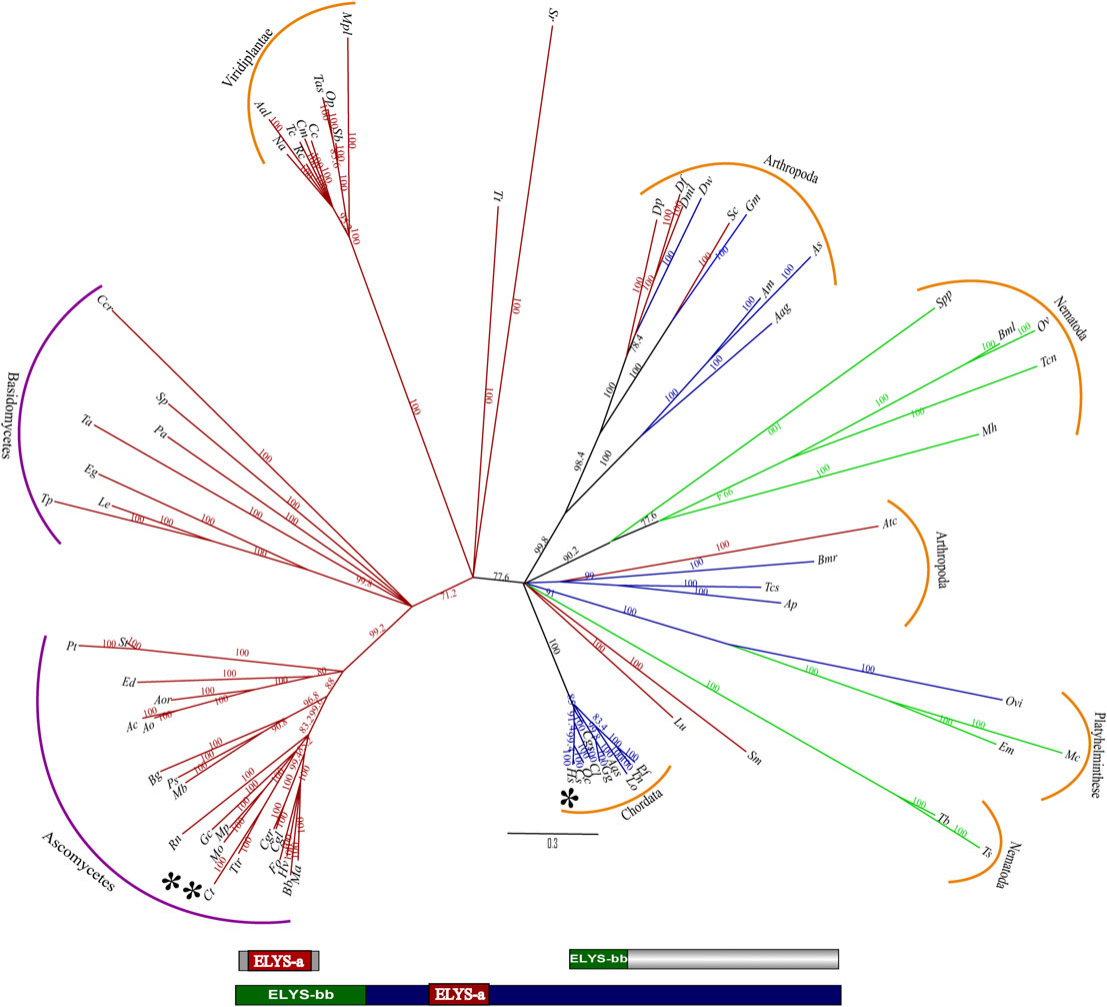
**Phylogenetic analysis and domain organization of ELYS homologs.** Based on structure‐guided multiple sequence alignment, representative homologs of ELYS were subjected to phylogenetic analysis (neighbor‐joining). In the unrooted tree shown, branch labels represent the percentage bootstrap values and the branch length is scaled to evolutionary distance. The branches are colored according to the type of domain present in different species, blue represents species predicted to have both ELYS‐bb and ELYS‐a domain. Green represents species predicted to have only ELYS‐bb domain and red depicts only ELYS‐a domain. The domain organization of the three classes of ELYS is depicted below the tree. The species names are abbreviated for the ease of representation and the detailed information is provided separately in Table S8 (* *H. sapiens* and ** *C. thermophilum*). The fungal species are grouped under purple color and the metazoan species under the orange color bars.

Embryonic large molecule derived from yolk sac (ELYS) has been identified as a transcription factor and was shown to interact with the outer ring complex of the NPC (Nup107‐Nup160).^75^ It is reported to be present in *C. thermophilum* though it is absent in *S. cerevisiae* and various other fungal species. When we analyzed the domain organization of *H. sapiens* ELYS, it was found to be composed of two domains namely ELYS‐bb [β propeller domain (PF16687.4)] and ELYS‐a [α helical domain (PF13934.5)] followed by a long unstructured region. In *C. thermophilum* ELYS, only the ELYS‐a (α domain) is present and the protein itself is smaller as compared to that of *H. sapiens* ELYS (299 amino acid in *C. thermophilum* vs. 2266 amino acid in human) (Fig. 3).

Through our phylogenetic analysis for ELYS of 77 representative species, we observed that there are many species, which have only the ELYS‐bb domain along with those similar in domain organization to *H. sapiens* and *C. thermophilum* (Fig. 3). The phylogenetic tree also indicates the lower organisms such as fungi have only the ELYS‐a domain and higher organism such as chordates have both ELYS‐bb and ELYS‐a domains. Interestingly, plants have only the ELYS‐a domain and nematode and platyhelminths have only ELYS‐bb domain. Arthropoda show the most interesting case as some of them contain only ELYS‐a domain such as *D. melanogaster* (Dml), *D. psuedobscura* (Dp), *D. ficusphila* (Df), *S. calcitrans* (Sc), *S. mimosarum* (Sm), and *A. cephalotes* (Atc) while some show both ELYS‐bb and ELYS‐a domain such as *D. willistoni* (Dw), *G. morsians* (Gm), *A. merus* (Am), *A. sinensis* (As), *A. aegypti* (Aag), *B. mori* (Bmr), *T. castaneum* (Tcs), and *A. planipennis* (Ap).

It is known that the β propeller region of ELYS is important for interaction with Nup160 of the Y‐shaped complex as well as with Nup37, which is exclusively present in vertebrates.^76^ The shorter ELYS‐a containing only the α helical region of *S. pombe* is known to interact with Nup120 of the Y‐shaped complex. The vertebrate ELYS is also known to help in the recruitment of POM121 and NDC1 to the NPC.^77^ Hence, the additional β propeller domain in human ELYS, strongly suggest that interactome of Nup107‐Nup160 complex is likely to be distinguished than its counterpart in fungal species.

#### *B. Insertion/deletion in Nup133 lead to different domain identification*

Nup133 is one of the major components of the Y‐shaped complex (outer ring complex) of the NPC.^5^ This protein is mostly present in all species ranging from fungi to metazoan and is known to be evolutionarily conserved. The secondary structure fold defined for Nup133 is a seven bladed β propeller domain followed by α helical domain. There are three structures known for these distinct domains, two for the β propeller region (PDB ID: 4Q9T [fungal] and 1XKS (vertebrate)) and one for partial α helical region (PDB ID: 3KFO (fungal)). Although in our sequence analysis we observed the difference in its PFAM domain identification between *H. sapiens*, *S. cerevisiae,* and *C. thermophilum*. For *H. sapiens*, Nup133 was predicted to have only a single PFAM domain Nucleoporin_C (defined as the non‐repetitive C‐terminal protein [PF03177]), whereas for *S. cerevisiae,* it was predicted to have a Nucleoporin_N domain [defined as N‐terminal half which forms seven‐bladed β propeller structure (PF08801)]. However, for *C. thermophilum*, both the domains were predicted to be present. This mis identification at the sequence level could be either due to insertion/deletions or sequence variability amounting from mutations. The structure‐guided alignment of these three representative species (Fig. S2) also depicts that there are insertions/ deletions at a number of places in the complete sequence which would account to additional secondary structures being formed.

**Figure 4:**
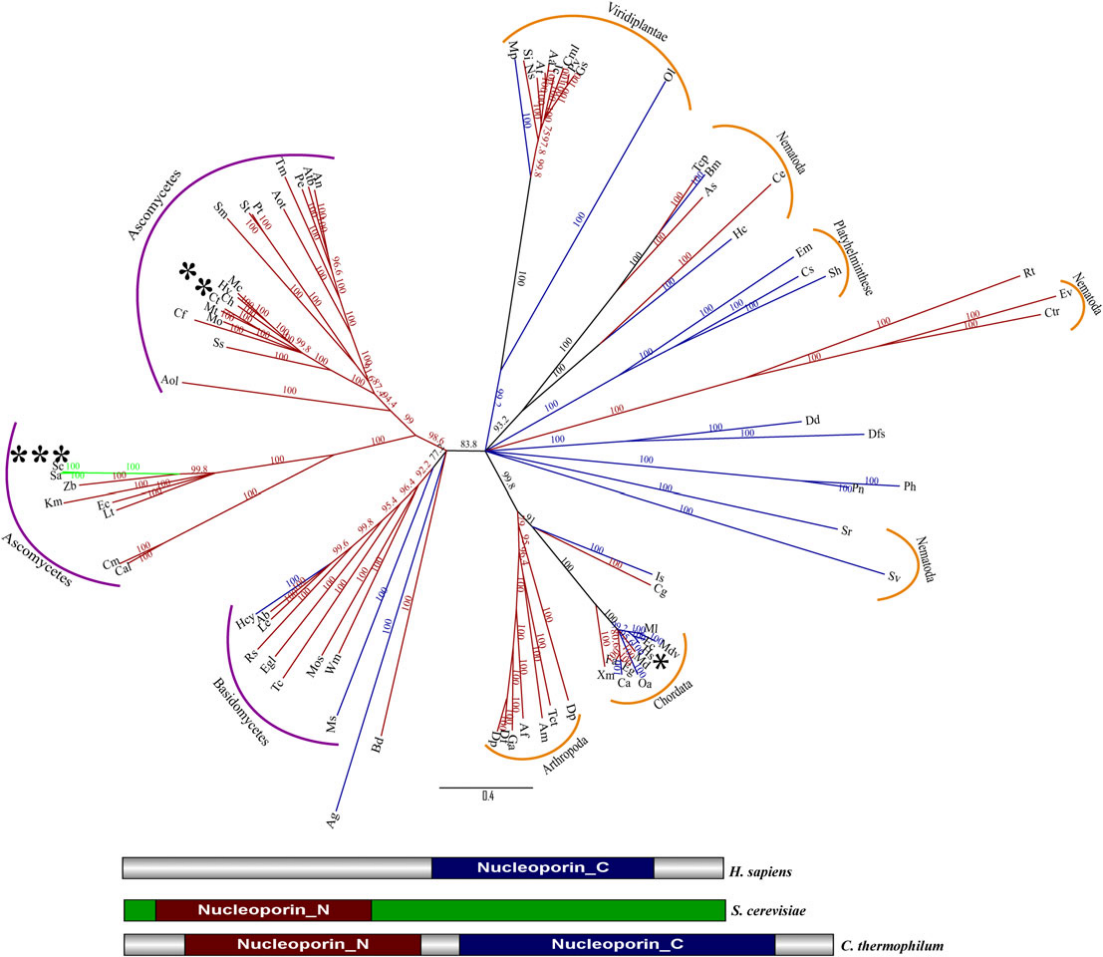
**Phylogenetic analysis and domain organization of Nup133 homologs.** Based on structure‐guided multiple sequence alignment, representative homologs of Nup133 were subjected to phylogenetic analysis (neighbor‐joining). In the unrooted tree, branch labels represent the percentage bootstrap values and the branch length is scaled to evolutionary distance. The branches are colored according to the type of domain present in different species, blue represents species predicted to have only Nucleoporin_C domain, green predicted to have only Nucleoporin_N domain and red depicts that both Nucleoporin_N and Nucleoporin_C were predicted for these group of species. The domain organization of the three classes of Nup133 is shown below the tree. The species names are abbreviated for the ease of representation and the detailed information is provided separately in Table S9 (* *H. sapiens*, ** *C. thermophilum*, and *** *S. cerevisiae*). The fungal species are grouped under purple color and the metazoan species under the orange color bars.

Our phylogenetic analysis of Nup133 in 84 representative species based on structure guided alignments revealed three distinct classes of domain organization. Fungi contain both Nucleoporin_N and Nucleoporin_C PFAM domain with an exception of *Saccharomyces* species, which were predicted to consist of only Nucleoporin_N PFAM domain as per HMMSCAN. Higher organisms, such as arthropods, nematodes, and plants were majorly predicted to have both the domains but chordates, platyhelminths, amoebozoan, and stramenophiles were predicted to have only Nucleoporin_C domain (Fig. 4).

To access the hypothesis of sequence variability which can be translated back to the structure as well, we conducted a selection pressure analysis on the residues of Nup133 by dividing the complete dataset of 84 representative sequences into three groups based on the domains that were predicted by HMMSCAN. These three groups were analyzed separately to calculate the Bayes Empirical Bayes (BEB) probability of a residue being under purifying (conserved) (dN/dS <1), neutral (dN/dS = 1) or positive (not conserved) (dN/dS > 1) selection pressure (Fig. S3). It was observed that in all the three groups a number of residues show a high probability of being under positive selection pressure. This would limit even as sensitive methods as HMMSCAN to correctly identify the domains.

We compared the structural organization of Nup133 by superimposing the available crystal structure of Nup133 N‐terminal domain from *H. sapiens* 31 (PDB ID: 1XKS) and *Vanderwaltozyma polyspora* (PDB ID: 4Q9T) and obtained an RMSD of 2.12 Å for the Cα atoms. It can be clearly observed that there are various insertions in both the structures depicting distinct secondary structure features at certain stretches (Fig. S4). We also mapped the positive selection sites onto the crystal structures of the Nup133 N‐terminal domain as discussed above to know their exact positions (Fig. S5). This analysis shows that the residues which do not superimpose well are mostly under positive selection pressure. Thus, from all these observations we may hypothesize that these insertion/deletions would reflect in the interaction network of the Y‐shaped complex in a species‐specific manner.

### *Structured region of central channel Nups depict differential selection pressures*

Three metazoan Nups (Nup62, Nup54, Nup58) and corresponding Nups in fungi (Nsp1, Nup57, Nup49) are known to form the lining of the central transport channel (CTC) of the NPC and provide the selective permeability barrier for the biomolecules across the nuclear envelope. Based on sequence analysis and secondary structure prediction, we observed that Nup62 is comparatively more conserved Nup among the species (average of 25% sequence identity and 35% sequence similarity between human and yeast). Human Nup54 has an extended structural domain (α/β region) of about 143 amino acids, which spans only 66 amino acids in yeast homologous Nups (Nup57). Interestingly, at the sequence similarity level, structured regions of human Nup58 and yeast Nup49 (determined using PSIPRED) showed low *e*‐values when *C. thermophilum* was used as a query and *H. sapiens* as the subject. The HMMER search with *C. thermophilum* as query could identify the human homolog at the fourth iteration with two regions of similarity (*e*‐values of 0.0005 and 4.4) (Fig. S1). Moreover, in terms of secondary structure, human Nup58‐structured region is completely α helical and is flanked by FG repeat regions at both ends whereas yeast Nup49 has a shorter structured α helical region and FG repeats are present only at the N‐terminus.

**Figure 5:**
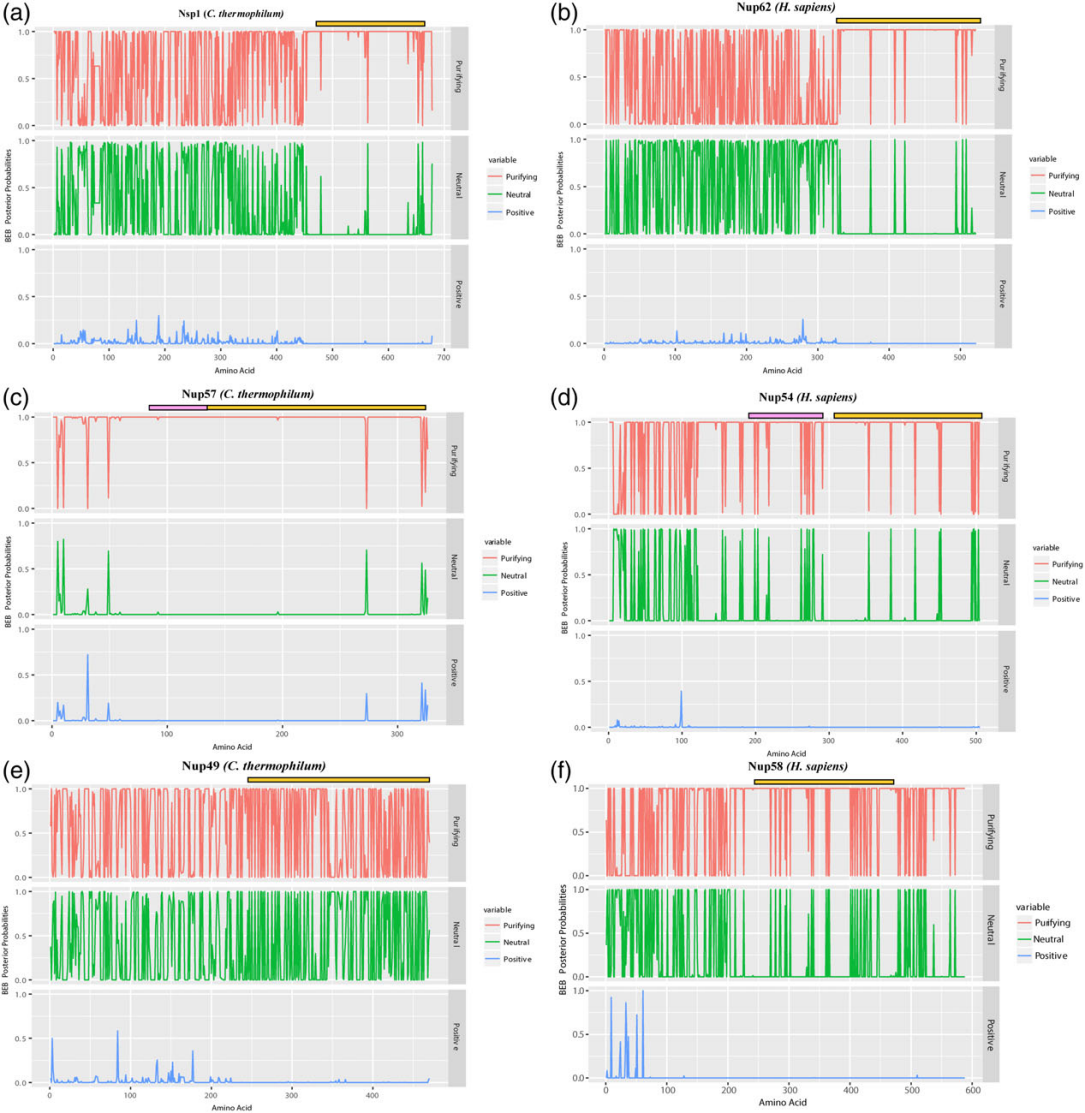
**Evolutionary pressure analysis on nucleoporins of the central channel.** Bayes empirical Bayes (BEB) probability obtained after dN/dS (ω) calculation is depicted for the three nucleoporins of the central channel of the NPC in both *C. thermophilum* (left column) and *H. sapiens* (right column) (a: Nsp1, b: Nup62, c: Nup57, d: Nup54, e: Nup49, f: Nup58). The BEB of residue positions under purifying selection pressure (ω < 1) are colored as red, neutral selection pressure (ω = 1) positions are colored as green and positive selection pressure (ω > 1) are colored as blue. The α helical regions are marked with the yellow bar in all the six Nups. The α/β region present in Nup57 and Nup54 is represented with a pink bar. The comparison of the structured regions of all the three Nups of the central channel in human and their orthologues in thermophilic yeast show a differential pattern of evolutionary pressures.

To understand the evolution of the structured regions of CTC proteins, we deployed the selection pressure analysis on these protein sequences. Although the full‐ length protein and nucleotide sequences were used for the analysis, we focused the results obtained only on the structured regions as predicted by PSIPRED since all the proteins of CTC show low complexity FG repeat regions. To avoid the impact of these low complexity regions, we used structure‐guided alignments [representative structure‐guided alignments of CTC proteins Nup62 (322–525 region, Fig. S6), Nup54 (190–507 region, Fig. S7), and Nup58 (249–475 region, Fig. S8)]. *C. thermophilum* was taken as the representative sequence of fungi central channel and *H. sapiens* as the representative sequence of the metazoan central channel. The results obtained are tabulated in Table 1 and Figure 5 (also refer to Supplementary Material for details).

**Table 1:** Percentage of Residues Under Purifying or Neutral Selection Pressure for the Structured Regions of Central Channel Proteins from Fungi and Metazoan Species

Nucleoporin (region of residues)	% of residues under purifying selection pressure	% of residues under neutral selection pressure
Nsp1 (466–678)	97	3
Nup62 (323–522)	94	6
Nup57 (74–325)	99	1
Nup54 (190–507)	94	6
Nup49 (245–470)	56	44
Nup58 (249–475)	88	12

Overall, in terms of evolution of the CTC proteins from fungi to metazoan, Nup62 can be called evolutionarily conserved. Nup54 gained an extended α/β region which is also under purifying selection pressure and its α helical region is also conserved between the fungal and metazoan species. However, Nup58 has diverged from its ancestral Nup49 since the α helical region is not as well conserved as the structured regions of other two CTC Nups. The presence of FG repeat region in Nup58 at C‐ terminus also being under purifying selection indicates a gain of additional FG domain in the metazoan lineage. This could also indicate different spatial orientation or tethering of Nup58 in the central channel of the NPC and hence gain of FG‐specific features in the vertebrate Nup58.

Additionally, the phylogenetic spread and domain prediction of Nup58 and its homologs from different phyla depict the presence of different PFAM domains (Nucleoporin_FG2 domain (described as a family of chordate nucleoporins (PF15967) and Nucleoporin_FG domain [represents the family of Nups having FG repeat regions (PF13634)] (Fig. S9). Since the major fraction of amino acids are not conserved in the homologous sequences of Nup58 as indicated through the selection pressure analysis, it might lead to different PFAM domains predictions.

**Figure 6:**
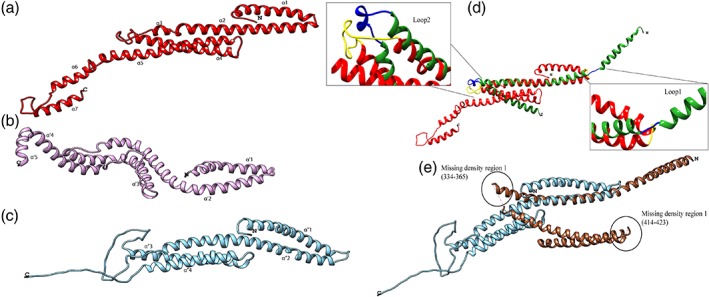
**Structural comparison of Nup58 from *H. sapiens* with Nup49 of the central channel from *S. cerevisiae* and *C. thermophilum*.** Structured region (α‐helix) of Nup58 from *H. sapiens* (red) and Nup49 from *S. cerevisiae* (purple) and *C. thermophilum* (cyan) was submitted as a query to pGenThreader for tertiary structure prediction. (a) Nup58 from *H. sapiens* (structured region only). (b) Nup49 from *S. cerevisiae* (structured region only). (c) Nup49 from *C. thermophilum* (structured region only). (d) Comparison of predicted Nup58 structure (red) with Nup58 chain of *Xenopus* Nup62 subcomplex (PDB ID: 5C3L) structure (green). Modulation in helix conformations can be observed when comparing the predicted structure with the experimentally solved structure. The residues marked in yellow on predicted Nup58 structure and light green on the Xenopus Nup58 chain show 100% identity in the first loop and 75% identity in the second loop [superimposition alignment in Fig. S10(a)] explaining the different conformations attained by the protein when present alone and when part of a triple helix bundle (Nup54‐Nup58‐Nup62). (e) Comparison of predicted Nup49 structure(cyan) from *C. thermophilum* with its crystal structure of Nup49 chain (brown) of Nsp1 complex (PDB ID: 5CWS). The modularity of the helices is not observed in this case and the residues are also not conserved when comparing the small loop regions present in the crystal structure [Fig. S10(b)]. The structures do not superimpose completely owing to the missing densities in the crystal structure (shown in circles). No coordinates were determined for Regions 334–365 and 414–423 which are also part of the coiled‐coil domain of Nup49.

To understand the three‐dimensional structural implications of these sequence variations on fungal Nup49 and metazoan Nup58, threading with pGenThreader^78^ was employed to model the tertiary structure folds of Nup58/Nup49 structured regions [hNup58(245–477), scNup49(267–472), and ctNup49(241–470)] [Figs. 6(a–c)]. The template used for generating these models is described in Table S7. Although both of the proteins folded as coiled‐coils, *H. sapiens* Nup58 consist of seven helices [α1–α7, Fig. 6(a)], *S. cerevisiae* is predicted to have five α‐helices [α’1–α’5, Fig. 6b) and *C. thermophilum* consists of only four helices [α”1–α”4, Fig. 6(c)]. An extended loop was observed between α’3 and α’4 (408–428) in *S. cerevisiae* and between α”2 and α”3 (343–380) in *C. thermophilum* which is absent in the predicted Nup58 structure from *H. sapiens*. Apart from this, the C‐terminus of Nup49 from *C. thermophilum* (450–470) is also predicted to be unstructured, whereas the corresponding region in both *S. cerevisiae* and *H. sapiens* consists of α helices.

Recently, two crystal structures of the CTC (Nup62•Nup54•Nup58) were described sourced from *Xenopus* (PDB ID: 5C3L)^47^ and from *Chaetomium* (PDB ID: 5CWS; along with 40 a.a. Nic96 interacting region).^6^ We further compared our predicted structures with the available X‐ray crystal structures to understand if any conformational modulation exists when Nup58/Nup49 is present alone and when it is present as a trimeric complex of the CTC. Since *Xenopus* is evolutionarily closer to *H. sapiens*, we compared the predicted human Nup58 structure with the Nup58 chain of *Xenopus* trimeric complex structure (PDB ID: 5C3L). The structured region of xNup58 (283–406) and hNup58 (249–374) show 88% sequence identity and 94% sequence similarity. The superimposition of the Nup58 modeled structure onto corresponding *Xenopus* Nup58 chain of trimeric structure (PDB ID: 5C3L, chain B) showed RMSD of 0.922 Å (for 30 aligned pair of Cα atoms) [Fig. 6(d)]. We could identify two regions, one being a loop (275–277) and other a helix‐turn‐helix (322–345) which may play a role in the conformational changes when this protein is present as a trimer in the Nup62 subcomplex [Fig. 6(d)]. Both these regions show high sequence similarity [Fig. S10(a)]. The first region has an identical three residues stretch (MSS) in both structures, which would lead to an untwisting of the first helix to attain the open conformation as explained in the *Xenopus* structure. The second region consists of a helix–loop–helix of 26 residues in hNup58 (320–346) out of which 19 are identical to *Xenopus* Nup58 crystal structure where this region corresponds to a loop–helix–loop conformation (xNup58 [364–375]). Based on all these observations, we propose that these regions could contribute to different conformations of hNup58 when present independently as opposed to as a complex with its other interacting partners (hNup62 and hNup54).

On the contrary, while comparing the predicted *C. thermophilum* Nup49 structure with the crystal structure of *Chaetomium's* central channel (PDB ID: 5CWS), similar loop regions as observed in Xenopus structure and hNup58 model, were not observed [Fig. 6(e)]. The loop between the first and second helix of both the predicted ctNup49 structure and ctNup49 crystal structure does not have any conserved residues [Fig. S10(b)]. It is also noteworthy that the crystal structure has two major regions with unresolved densities (334–365 and 414–423) owing to which the superimposition is also not complete (RMSD of 1.06 Å for 25 aligned pair of Cα atoms). We also compared Nup58 chain of 5C3L and Nup49 chain of 5CWS and observed that they superimpose with an RMSD of 1.09 (for 68 aligned pair of Cα atoms). This suggests that in presence of other interacting partners (Nup62•Nup54 and Nsp1•Nup57, respectively) as well as the stabilizing agents (Fab and nanobody, respectively) Nup58/Nup49 undergoes major conformational alterations. It is evident from crystal structures of *R. norvegicus* CTC proteins that they exist in different quaternary assemblies when present as an independent protein (PDB ID: 5H1X, 4J3H, 2OSZ)^21^, ^32^, ^73^ as compared to when in complex with one of the interacting partners (PDB ID: 3T97, 3T98).^50^ For example, Nup54 when present as a single entity attains a homo‐tetramer conformation (PDB ID: 4J3H),^45^ and when present in complex with Nup62 forms a heterotrimer, with two chains of Nup62 and a single chain of Nup54 (PDB ID: 3T97).^50^ Similarly, when Nup54 is present in complex with Nup58 it forms a heterotrimer with two chains of Nup54 and a single chain of Nup58 (PDB ID: 3T98).^50^ Thus, validating our tertiary structure prediction of Nup58 α helical region to exhibit a compact conformation when present as an independent entity as compared to an open conformation when present as a complex (as seen in Xenopus crystal structure).

These differences in tertiary structure, as well as the absence of FG repeats on the C‐terminus of yeast Nup49 may contribute to the distinct structural and functional role of Nup58 in the metazoan NPC and Nup49 in the fungi NPC, and also is likely to influence the interaction with other nucleoporins as well as the cargo molecules. All these observations taken together indicate the presence of a metazoan‐specific Nup58 and fungi‐specific Nup49 of the CTC.

**Figure 7:**
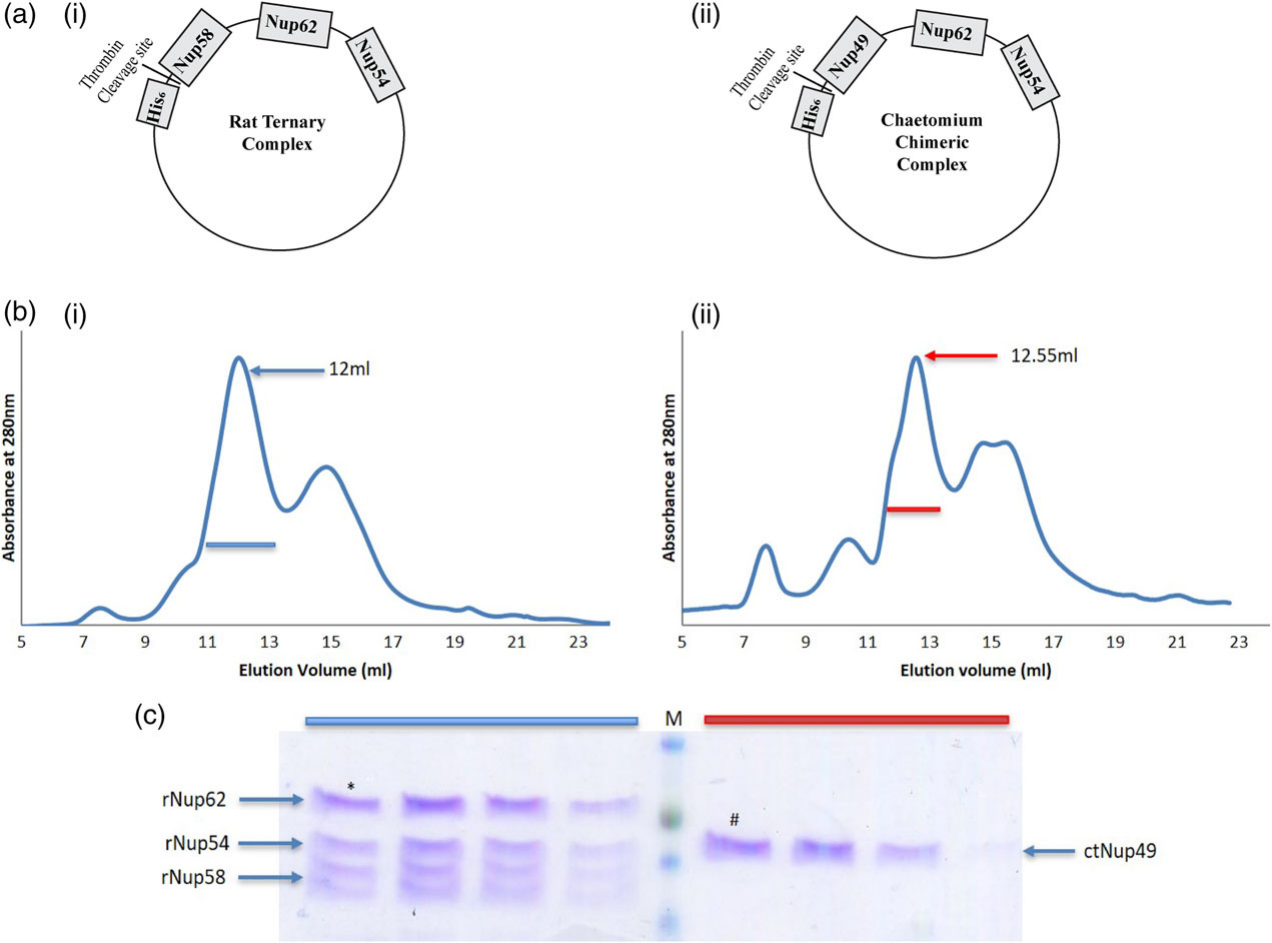
**In vitro pull‐down assay of Nup54 and Nup62 of central channel through interspecies Nup58.** (a) Schematic representation of cloned constructs. (i) Rat ternary complex and (ii) Chaetomium chimeric complex. (b) Size exclusion chromatogram of (i) three‐protein complex of rat central channel (Nup62, Nup54, and Nup58) after His_6_ tag cleavage. The fourth band is a proteolytic product of Nup58 (Nup45) (ii) chimeric construct after His_6_ tag cleavage. (c) 12% SDS‐PAGE analysis of the peak fractions marked with blue bar (rat) and red bar (Chaetomium) in b(i) and b(ii). * represents the elution fraction at 12 mL in case of rat and # represents elution fraction at 12.55 mL in case of Chaetomium. (ct: *C. thermophilum* r: *R. norvegicus*).

To validate that indeed the interaction network of both the CTC proteins i.e. Nup58/Nup49 would be different we devised an in vitro pull‐down assay. It has been shown previously that *C. thermophilum* Nup49 forms a stable complex with Nup57 and Nsp1^6^ and similarly their homolog in *R. norvegicus* forms a Nup58‐Nup54‐Nup62 complex.^46^ We generated a polycistronic plasmid to express chimeric complex ctNup49 with rNup54 and rNup62 [Fig. 7(aii)]. We observed that in case of chimeric complex ctNup49 eluted as a single protein [Fig. S11(a)] whereas for rat ternary complex, Nup58 pulled out Nup62 and Nup54 along with it in the given identical conditions of pull‐down assay [Fig. S11(b)]. The presence of His_6_‐tagged proteins (ctNup49 and rNup58) were confirmed with Anti‐His antibody western blot [Fig. S11(c)] in pull down assays. Subsequently, the gel filtration elution profile in case of rat ternary complex depicts the presence of stable three protein complex [Fig. 7(b(i))]. However, for the Chaetomium chimeric construct, a single protein corresponding to the molecular weight of ctNup49 [Fig. 7(b(ii))] was observed supporting our hypothesis that Nup49 could not complement the interaction interface of Nup58 for the rat ternary complex formation.

### *Supertree of nuclear pore complex depicts divergent evolution*

To understand the evolution pattern of all the nucleoporins taken together, supertree approach^79^ was deployed where individual trees of all nucleoporins i.e. 35 source trees (including the ones unique to metazoans and fungi) were used as input. Compiling all the information available from the 35 input trees which were build based on structure‐guided multiple sequence alignments, the nucleoporins may be divided into three different classes. Class one includes all the nucleoporins which are evolutionarily conserved namely Nup62, Nup93, Nup205, Nup155, Nup188, NDC1, TMEM33, Nup107, Nup85, Sec13, Seh1, Nup133, Gle1, and Rae 1. These trees show the distribution of species as per the tree of life. Class two includes nucleoporins which are not evolutionary conserved and the species do not follow the distribution as per the tree of life namely Nup54, Nup58, Nup35, ELYS, Nup88, Nup214, Nup160, Nup98, Nup50, and TPR. The third class includes nucleoporins which are unique to either metazoans or fungi. Gp210, Nup37, Nup43, and Aladin are unique to metazoans; Pom121 is present only in Chordates. Pom152 is only present in fungi but is conserved across all the families of fungi. Nup60 and Pom34 are present only in Ascomycetes.

**Figure 8:**
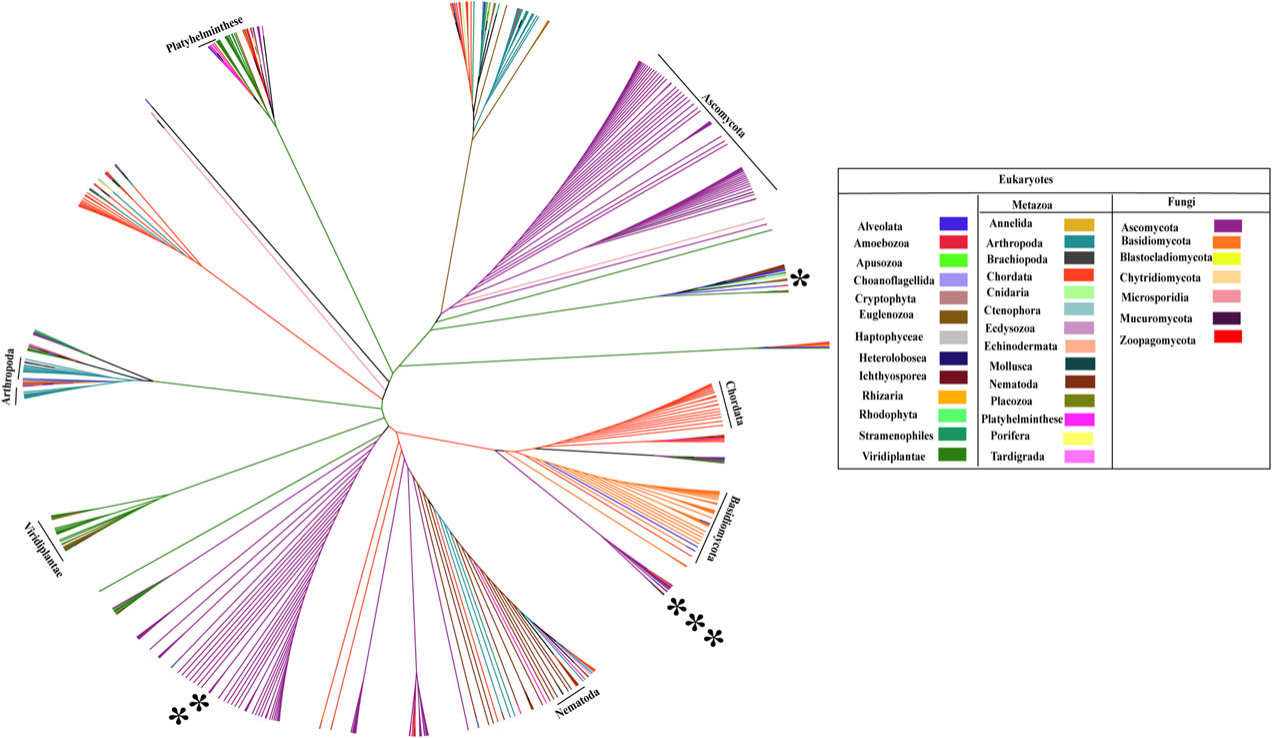
**Supertree of the nuclear pore complex**. Supertree constructed using phylogenetic trees of 35 nucleoporins (including the ones unique to metazoan and fungi). The supertree contains 631 species which were classified into 34 phylum/class/order and are colored according to the legend depicted. This is an unrooted tree where the branch lengths were transformed to equal proportions for clear representation. The tree was visualized and annotated using Figtree (http://tree.bio.ed.ac.uk/software/figtree/). List of species names and their classification are tabulated in Table S11. As represented in the tree, the lower organisms group together into separate clades but the higher organisms (namely, Chordata, Viridiplantae) show segregated distribution throughout the supertree indicating divergent evolution of nucleoporins among distinct taxa. (* *H. sapiens*, ** *C. thermophilum,* and *** *S. cerevisiae*).

Considering all these observations of the independent phylogenetic trees, we obtained a supertree depicting divergent evolution of nucleoporins across 613 species (Fig. 8). Interestingly, when we look at the positions of higher organism species such as those belonging to Viridiplantae, Arthropoda, and Chordata (colored green, cyan, and orange, respectively) in the supertree (Fig. 8), they do not form demarcated clades but are intermingled with each other. Such an observation could be either because of under representation of a particular species due to compositional differences, or due to the divergence of the sequences from the parental ones. In both scenarios, it is evident that the higher organisms have diverged to a great extent from their ancestral forms owing to increasing complexity at the sequence level, due to insertion/deletions/mutations leading to changes in the sequence length, presence/absence of certain domains as well changes in the secondary structure organizations. This analysis is thus indicative of a divergent evolution of nucleoporins. However, the sequence search space is limited to the current sequence data available.

While constructing individual trees of nucleoporins, structure‐guided alignments were taken into consideration, suggesting that this supertree also depicts structure‐guided sequence evolution of the NPC. Although in various recent studies, comparisons have been made between the crystal structure of fungi (*C. thermophilum* and *S. cerevisiae*) and tomographic structure of *H. sapiens* but this analysis suggests that these comparisons should be made with caution. As observed in the supertree also these three species are present at diverged positions from each other in terms of structure‐guided evolution of sequences.

## Conclusions

To completely decipher the complex protein–protein interaction network in this multiprotein complex of the cell, it is important to keep in mind the role of evolution on both the sequence and the structure of individual proteins. It has been reported through large‐scale interactome studies that two types of evolutionary forces exist on the interfaces of interacting proteins. One is to resist any change on the interacting interfaces to maintain the functional relevance of that interaction for evolutionarily conserved proteins. The other being to accept mutations in a way to develop new interacting interfaces with proteins which might perform the similar function but are not evolutionarily conserved.^80^ NPC might be the perfect example to observe both these evolutionary forces acting at the same time and thus leading to a species‐specific interaction network. To support our view, two recent reports on tomography structure of NPC from *Chlamydomonas reinhardtii* 81 and *S. cerevisiae* 82 depict the architectural differences in the arrangement of major subcomplexes as compared to the *H. sapiens* 26 NPC. Hence, it is important to elucidate the structural information of NPC in species‐specific context to fully understand its transport functions. Although there would be some similarities between metazoan and fungal NPC, its structural details are likely to be significantly different from other species to accommodate more complex transport functions to tackle cellular and tissue‐specific functional specificity.

## Materials and Methods

### *Secondary structure prediction*

The secondary structure prediction was performed using PSIPRED online server psipred version 3.3^83^ for all the nucleoporins from *H. sapiens*, *S. cerevisiae,* and *C. thermophilum*. A comparative chart containing all the predicted secondary structure for these three species was sketched in IBS illustrator.^84^

### *Phylogenetic analysis*

Only the representative sequences generated by PROMALS3D^85^ were considered to reduce the size of the dataset as well as the computation time for all the nucleoporins, including the ones exclusively present in *H. sapiens* and *S. cerevisiae*. The edited multiple sequence alignments were saved in phylip format to run through phylogenetic analysis using Phylip (http://evolution.genetics.washington.edu/phylip.html). The alignments were bootstrapped 500 times as a test of phylogeny. Neighbor‐joining method was used to construct unrooted trees from the distance matrices obtained. A consensus tree was generated using the program SumTrees of DendroPy^86^ at a 75% majority rule. The advantage of using SumTrees program is it retains the branch length information which might be lost while using other standard consensus programs and results in a phylogram with branch lengths scaled to the evolutionary distances. The phylogenetic trees were visualized using FigTree software version 1.4.2 (http://tree.bio.ed.ac.uk/software/figtree/).

### *Evolutionary pressure analysis*

The evolutionary pressure analysis was performed on nucleoporins of the central channel (namely, Nup62, Nup54, and Nup58) from *H. sapiens* and their homologous (namely, Nsp1, Nup57, and Nup49) from *C. thermophilum*. Nucleotide sequences for all these proteins from different species were obtained from the Uniport database search. The nucleotide sequences were then translated to protein sequences and then structure‐guided multiple sequence alignment was performed on the amino acid sequences. Using the structure‐guided alignment as reference the nucleotide sequences were aligned using DAMBE5.^87^ Maximum‐likelihood phylogenetic analysis was performed on the aligned sequences using phylip. The final maximum‐likelihood tree and nucleotide alignment obtained was used for evolutionary pressure analysis using the “codeml” program of PAML.^88^ PAMLX^89^ a GUI interface was used to set the parameters for these calculations. The final output obtained for each dataset i.e. Bayes Empirical Bayes probabilities were then plotted using ggplot2 and reshape2 package in Rstudio (R studio Team [2015]. Rstudio: Integrated Development for R. Rstudio, Insc., Boston, MA, URL http://www.rstudio.com/).

### *Tertiary structure prediction*

Tertiary structure prediction using a threading approach was performed for Nup58 of *H. sapiens* and its homolog Nup49 of *S. cerevisiae* and *C. thermophilum*. pGenThreader^78^ was used to search for best template against the sequence of these nucleoporins from the three species. The best hit which had the lowest *P*‐value and maximum query coverage were used to model the sequence onto the structure. Since both Nup58 and Nup49 contain FG repeat regions/unstructured regions the query was designed to contain only the structured region sequence based on secondary structure predictions from PSIPRED. The templates used for generation of models are described in Table S7. The predicted structures were visualized and analyzed using UCSF software Chimera.^90^

### *Cloning and purification of rat ternary and chaetomium chimeric construct*

The structured region of central channel complex from *Rattus norvegicus* was cloned in modified pET28a as described earlier.^46^ The construct contains thrombin cleavage site at the N terminus of His_6_ tagged Nup58 (239–415) followed by Nup62 (322–525) and Nup54 (332–510). The conserved region of Nup58 in *Chaetomium thermophilum* Nup49 (246–470) was gene synthesized (Invitrogen) and replaced on the above construct. Both the constructs were subjected to the same protocol for Ni‐NTA affinity purification.^46^ Briefly, BL21(DE3)‐RIL strain of *Escherichia coli* was transformed with the construct and the culture(2 L) was induced with 0.5 mM IPTG at OD ~0.6 followed by incubation of 8 h at 18°C and purified using Ni‐NTA agarose bead (Qiagen). The purified protein complex was dialysed against buffer (Tris–HCl pH 8, 250 mM NaCl, 1 mM DTT) and digested with thrombin at 4°C. The digested protein was concentrated using a 3 kDa cutoff concentrator (Merck) and subjected for SEC using superdex 200, 10/300 GL column (GE Healthcare) in SEC buffer (Tris–HCl pH 8, 250 mM NaCl, 0.5 mM EDTA, 1 mM DTT) at 4°C.

### *Supertree construction*

A supertree can be constructed if there are numerous species trees of different genes with at least few overlapping species in all trees. All the phylogenetic trees (35 in total with approximately 80 representative species in each tree) were used as input to construct a supertree by an average consensus method using the program clann.^79^ Nearest neighbor approach was utilized in merging the phylogenetic information from all the trees under consideration. The final supertree was visualized as a cladogram using FigTree software version 1.4.2 (http://tree.bio.ed.ac.uk/software/figtree/).

## Supporting information


**Appendix S1:** Supplementary Information.

## References

[1] Keminer O , Peters R (1999) Permeability of single nuclear pores. Biophys J 77:217–228. 10388751 10.1016/S0006-3495(99)76883-9 PMC1300323

[2] Akey CW , Radermacher M (1993) Architecture of the Xenopus nuclear pore complex revealed by three‐dimensional cryo‐electron microscopy. J Cell Biol 122:1–19. 8314837 10.1083/jcb.122.1.1 PMC2119598

[3] Rout MP , Aitchison JD , Suprapto A , Hjertaas K , Zhao Y , Chait BT (2000) The yeast nuclear pore complex: composition, architecture, transport mechanism. J Cell Biol 148:635–651. 10684247 10.1083/jcb.148.4.635 PMC2169373

[4] Cronshaw JM , Krutchinsky AN , Zhang W , Chait BT , Matunis MJ (2002) Proteomic analysis of the mammalian nuclear pore complex. J Cell Biol 158:915–927. 12196509 10.1083/jcb.200206106 PMC2173148

[5] Belgareh N , Rabut G , Baï SW , Van Overbeek M , Beaudouin J , Daigle N , Zatsepina OV , Pasteau F , Labas V , Fromont‐Racine M , Ellenberg J , Doye V (2001) An evolutionarily conserved NPC subcomplex, which redistributes in part to kinetochores in mammalian cells. J Cell Biol 154:1147–1160. 11564755 10.1083/jcb.200101081 PMC2150808

[6] Stuwe T , Bley CJ , Thierbach K , Petrovic S , Schilbach S , Mayo DJ , Perriches T , Rundlet EJ , Jeon YE , Collins LN , Huber FM , Lin DH , Paduch M , Koide A , Lu V , Fischer J , Hurt E , Koide S , Kossiakoff AA , Hoelz A (2015) Architecture of the fungal nuclear pore inner ring complex. Science 350:56–64. 26316600 10.1126/science.aac9176 PMC4826903

[7] Grandi P , Dang T , Pané N , Shevchenko A , Mann M , Forbes D , Hurt E (1997) Nup93, a vertebrate homologue of yeast Nic96p, forms a complex with a novel 205‐kDa protein and is required for correct nuclear pore assembly. Mol Biol Cell 8:2017–2038. 9348540 10.1091/mbc.8.10.2017 PMC25664

[8] Eisenhardt N , Redolfi J , Antonin W (2014) Interaction of Nup53 with Ndc1 and Nup155 is required for nuclear pore complex assembly. J Cell Sci 127:908–921. 24363447 10.1242/jcs.141739

[9] Krull S , Thyberg J , Bjorkroth B , Rackwitz H‐R , Cordes VC (2004) Nucleoporins as components of the nuclear pore complex core structure and Tpr as the architectural element of the nuclear basket. Mol Biol Cell 15:4261–4277. 15229283 10.1091/mbc.E04-03-0165 PMC515357

[10] Reichelt R , Holzenburg A , Buhle EL , Engel A (1990) Correlation between structure and mass distribution of the nuclear pore complex and of distinct pore complex components. J Cell Biol 110:883–894. 2324201 10.1083/jcb.110.4.883 PMC2116066

[11] Rout MP , Blobel G (1993) Isolation of the yeast nuclear pore complex. J Cell Biol 123:771–783. 8227139 10.1083/jcb.123.4.771 PMC2200146

[12] Mizuguchi‐Hata C , Ogawa Y , Oka M , Yoneda Y (2013) Quantitative regulation of nuclear pore complex proteins by O‐GlcNAcylation. Biochim Biophys Acta 1833:2682–2689. 23777819 10.1016/j.bbamcr.2013.06.008

[13] Nino CA , Guet D , Gay A , Brutus S , Jourquin F , Mendiratta S , Salamero J , Geli V , Dargemont C (2016) Posttranslational marks control architectural and functional plasticity of the nuclear pore complex basket. J Cell Biol 212:167–180. 26783300 10.1083/jcb.201506130 PMC4738382

[14] Osmani AH , Davies J , Liu H‐L , Nile A , Osmani SA (2006) Systematic deletion and mitotic localization of the nuclear pore vomplex proteins of *Aspergillus nidulans* . Mol Biol Cell 17:4946–4961. 16987955 10.1091/mbc.E06-07-0657 PMC1679664

[15] Asakawa H , Yang H‐J , Yamamoto TG , Ohtsuki C , Chikashige Y , Sakata‐Sogawa K , Tokunaga M , Iwamoto M , Hiraoka Y , Haraguchi T (2014) Characterization of nuclear pore complex components in fission yeast *Schizosaccharomyces pombe* . Nucleus 5:149–162. 24637836 10.4161/nucl.28487 PMC4049921

[16] Amlacher S , Sarges P , Flemming D , Van Noort V , Kunze R , Devos DP , Arumugam M , Bork P , Hurt E (2011) Insight into structure and assembly of the nuclear pore complex by utilizing the genome of a eukaryotic thermophile. Cell 146:277–289. 21784248 10.1016/j.cell.2011.06.039

[17] Tamura K , Fukao Y , Iwamoto M , Haraguchi T , Hara‐Nishimura I (2010) Identification and characterization of nuclear pore complex components in *Arabidopsis thaliana* . Plant Cell 22:4084–4097. 21189294 10.1105/tpc.110.079947 PMC3027183

[18] Galy V , Mattaj IW , Askjaer P (2003) *Caenorhabditis elegans* nucleoporins Nup93 and Nup205 determine the limit of nuclear pore complex size exclusion in vivo. Mol Biol Cell 14:5104–5115. 12937276 10.1091/mbc.E03-04-0237 PMC284812

[19] Obado SO , Brillantes M , Uryu K , Zhang W , Ketaren NE , Chait BT , Field MC , Rout MP (2016) Interactome mapping reveals the evolutionary history of the nuclear pore complex. PLoS Biol 14:e1002365. 26891179 10.1371/journal.pbio.1002365 PMC4758718

[20] Iwamoto M , Osakada H , Mori C , Fukuda Y , Nagao K , Obuse C , Hiraoka Y , Haraguchi T (2017) Compositionally distinct nuclear pore complexes of functionally distinct dimorphic nuclei in ciliate Tetrahymena. J Cell Sci 130:1822–1834. 28386019 10.1242/jcs.199398 PMC5450191

[21] Neumann N , Lundin D , Poole AM (2010) Comparative genomic evidence for a complete nuclear pore complex in the last eukaryotic common ancestor. PLoS One 5:e13241. 20949036 10.1371/journal.pone.0013241 PMC2951903

[22] Promponas VJ , Katsani KR , Blencowe BJ , Ouzounis CA (2016) Sequence evidence for common ancestry of eukaryotic endomembrane coatomers. Sci Rep 6:22311. 26931514 10.1038/srep22311 PMC4773986

[23] Devos D , Dokudovskaya S , Williams R , Alber F , Eswar N , Chait BT , Rout MP , Sali A (2006) Simple fold composition and modular architecture of the nuclear pore complex. Proc Natl Acad Sci U S A 103:2172–2177. 16461911 10.1073/pnas.0506345103 PMC1413685

[24] Beck M , Lucić V , Förster F , Baumeister W , Medalia O (2007) Snapshots of nuclear pore complexes in action captured by cryo‐electron tomography. Nature 449:611–615. 17851530 10.1038/nature06170

[25] Maimon T , Elad N , Dahan I , Medalia O (2012) The human nuclear pore complex as revealed by cryo‐electron tomography. Structure 20:998–1006. 22632834 10.1016/j.str.2012.03.025

[26] Eibauer M , Pellanda M , Turgay Y , Dubrovsky A , Wild A , Medalia O (2015) Structure and gating of the nuclear pore complex. Nat Commun 6:7532. 26112706 10.1038/ncomms8532 PMC4491817

[27] Hodel AE , Hodel MR , Griffis ER , Hennig KA , Ratner GA , Xu S , Powers MA (2002) The three‐dimensional structure of the autoproteolytic, nuclear pore‐targeting domain of the human nucleoporin Nup98. Mol Cell 10:347–358. 12191480 10.1016/s1097-2765(02)00589-0

[28] Sun Y , Guo H‐C (2008) Structural constraints on autoprocessing of the human nucleoporin Nup98. Protein Sci 17:494–505. 18287282 10.1110/ps.073311808 PMC2248301

[29] Napetschnig J , Blobel G , Hoelz A (2007) Crystal structure of the N‐terminal domain of the human protooncogene Nup214/CAN. Proc Natl Acad Sci 104:1783–1788. 17264208 10.1073/pnas.0610828104 PMC1794303

[30] von Moeller H , Basquin C , Conti E (2009) The mRNA export protein DBP5 binds RNA and the cytoplasmic nucleoporin NUP214 in a mutually exclusive manner. Nat Struct Mol Biol 16:247–254. 19219046 10.1038/nsmb.1561

[31] Berke IC , Boehmer T , Blobel G , Schwartz TU (2004) Structural and functional analysis of Nup133 domains reveals modular building blocks of the nuclear pore complex. J Cell Biol 167:591–597. 15557116 10.1083/jcb.200408109 PMC2172596

[32] Xu C , Li Z , He H , Wernimont A , Li Y , Loppnau P , Min J (2015) Crystal structure of human nuclear pore complex component NUP43. FEBS Lett 589:3247–3253. 26391640 10.1016/j.febslet.2015.09.008

[33] Ren Y , Seo H‐S , Blobel G , Hoelz A (2010) Structural and functional analysis of the interaction between the nucleoporin Nup98 and the mRNA export factor Rae1. Proc Natl Acad Sci U S A 107:10406–10411. 20498086 10.1073/pnas.1005389107 PMC2890840

[34] Reverter D , Lima CD (2005) Insights into E3 ligase activity revealed by a SUMO–RanGAP1–Ubc9–Nup358 complex. Nature 435:687–692. 15931224 10.1038/nature03588 PMC1416492

[35] Lin DH , Zimmermann S , Stuwe T , Stuwe E , Hoelz A (2013) Structural and functional analysis of the C‐terminal domain of Nup358/RanBP2. J Mol Biol 425:1318–1329. 23353830 10.1016/j.jmb.2013.01.021 PMC4226655

[36] Gareau JR , Reverter D , Lima CD (2012) Determinants of small ubiquitin‐like modifier 1 (SUMO1) protein specificity, E3 ligase, and SUMO‐RanGAP1 binding activities of nucleoporin RanBP2. J Biol Chem 287:4740–4751. 22194619 10.1074/jbc.M111.321141 PMC3281653

[37] Kassube SA , Stuwe T , Lin DH , Antonuk CD , Napetschnig J , Blobel G , Hoelz A (2012) Crystal structure of the N‐terminal domain of Nup358/RanBP2. J Mol Biol 423:752–765. 22959972 10.1016/j.jmb.2012.08.026 PMC4226657

[38] Pumroy RA , Nardozzi JD , Hart DJ , Root MJ , Cingolani G (2012) Nucleoporin Nup50 stabilizes closed conformation of armadillo repeat 10 in importin α5. J Biol Chem 287:2022–2031. 22130666 10.1074/jbc.M111.315838 PMC3265882

[39] Jinek M , Rehwinkel J , Lazarus BD , Izaurralde E , Hanover JA , Conti E (2004) The superhelical TPR‐repeat domain of O‐linked GlcNAc transferase exhibits structural similarities to importin [alpha]. Nat Struct Mol Biol 11:1001–1007. 15361863 10.1038/nsmb833

[40] Napetschnig J , Kassube SA , Debler EW , Wong RW , Blobel G , Hoelz A (2009) Structural and functional analysis of the interaction between the nucleoporin Nup214 and the DEAD‐box helicase Ddx19. Proc Natl Acad Sci U S A 106:3089–3094. 19208808 10.1073/pnas.0813267106 PMC2651337

[41] Matsuura Y , Stewart M (2005) Nup50/Npap60 function in nuclear protein import complex disassembly and importin recycling. EMBO J 24:3681–3689. 16222336 10.1038/sj.emboj.7600843 PMC1276725

[42] Handa N , Kukimoto‐Niino M , Akasaka R , Kishishita S , Murayama K , Terada T , Inoue M , Kigawa T , Kose S , Imamoto N , Tanaka A , Hayashizaki Y , Shirouzu M , Yokoyama S (2006) The crystal structure of mouse Nup35 reveals atypical RNP motifs and novel homodimerization of the RRM domain. J Mol Biol 363:114–124. 16962612 10.1016/j.jmb.2006.07.089

[43] Bilokapic S , Schwartz TU (2013) Structural and functional studies of the 252 kDa nucleoporin ELYS reveal distinct roles for its three tethered domains. Structure 21:572–580. 23499022 10.1016/j.str.2013.02.006 PMC4077343

[44] Dewangan PS , Sonawane PJ , Chouksey AR , Chauhan R (2017) The Nup62 coiled‐coil motif provides plasticity for triple‐helix bundle formation. Biochemistry 56:2803–2811. 28406021 10.1021/acs.biochem.6b01050

[45] Solmaz SR , Blobel G , Melcak I (2013) Ring cycle for dilating and constricting the nuclear pore. Proc Natl Acad Sci 110:5858–5863. 23479651 10.1073/pnas.1302655110 PMC3625290

[46] Ivo M , Hoelz A , Blobel G (2007) Structure of Nup58/45 suggests flexible nuclear pore diameter by intermolecular sliding. Science 315:1729–1733. 17379812 10.1126/science.1135730

[47] Chug H , Trakhanov S , Hülsmann BB , Pleiner T , Görlich D (2015) Crystal structure of the metazoan Nup62•Nup58•Nup54 nucleoporin complex. Science 350:106–110. 26292704 10.1126/science.aac7420

[48] Whittle JRR , Schwartz TU (2009) Architectural nucleoporins Nup157/170 and Nup133 are structurally related and descend from a second ancestral element. J Biol Chem 284:28442–28452. 19674973 10.1074/jbc.M109.023580 PMC2788893

[49] Boehmer T , Jeudy S , Berke IC , Schwartz TU (2008) Structural and functional studies of Nup107/Nup133 interaction and its implications for the architecture of the nuclear pore complex. Mol Cell 30:721–731. 18570875 10.1016/j.molcel.2008.04.022 PMC2446439

[50] Solmaz SR , Chauhan R , Blobel G , Melčák I (2011) Molecular architecture of the transport channel of the nuclear pore complex. Cell 147:590–602. 22036567 10.1016/j.cell.2011.09.034 PMC3431207

[51] Sampathkumar P , Kim SJ , Upla P , Rice WJ , Phillips J , Timney BL , Pieper U , Bonanno JB , Fernandez‐Martinez J , Hakhverdyan Z , Ketaren NE , Matsui T , Weiss TM , Stokes DL , Sauder JM , Burley SK , Sali A , Rout MP , Almo SC (2013) Structure, dynamics, evolution, and function of a major scaffold component in the nuclear pore complex. Structure 21:560–571. 23499021 10.1016/j.str.2013.02.005 PMC3755625

[52] Weirich CS , Erzberger JP , Berger JM , Weis K (2004) The N‐terminal domain of Nup159 forms a beta‐propeller that functions in mRNA export by tethering the helicase Dbp5 to the nuclear pore. Mol Cell 16:749–760. 15574330 10.1016/j.molcel.2004.10.032

[53] Jeudy S , Schwartz TU (2007) Crystal structure of nucleoporin Nic96 reveals a novel, intricate helical domain architecture. J Biol Chem 282:34904–34912. 17897938 10.1074/jbc.M705479200

[54] Schrader N , Stelter P , Flemming D , Kunze R , Hurt E , Vetter IR (2008) Structural basis of the Nic96 subcomplex organization in the nuclear pore channel. Mol Cell 29:46–55. 18206968 10.1016/j.molcel.2007.10.022

[55] Leksa NC , Brohawn SG , Schwartz TU (2009) The structure of the scaffold nucleoporin Nup120 reveals a new and unexpected domain architecture. Structure 17:1082–1091. 19576787 10.1016/j.str.2009.06.003 PMC2743489

[56] Seo H‐S , Ma Y , Debler EW , Wacker D , Kutik S , Blobel G , Hoelz A (2009) Structural and functional analysis of Nup120 suggests ring formation of the Nup84 complex. Proc Natl Acad Sci U S A 106:14281–14286. 19706512 10.1073/pnas.0907453106 PMC2732846

[57] Sampathkumar P , Ozyurt SA , Do J , Bain KT , Dickey M , Rodgers LA , Gheyi T , Sali A , Kim SJ , Phillips J , Pieper U , Fernandez‐Martinez J , Franke JD , Martel A , Tsuruta H , Atwell S , Thompson DA , Emtage JS , Wasserman SR , Rout MP , Sauder JM , Burley SK (2010) Structures of the autoproteolytic domain from the *Saccharomyces cerevisiae* nuclear pore complex component, Nup145. Proteins 78:1992–1998. 20310066 10.1002/prot.22707 PMC3136511

[58] Sampathkumar P , Gheyi T , Miller SA , Bain KT , Dickey M , Bonanno JB , Kim SJ , Phillips J , Pieper U , Fernandez‐Martinez J , Franke JD , Martel A , Tsuruta H , Atwell S , Thompson DA , Emtage JS , Wasserman SR , Rout MP , Sali A , Sauder JM , Burley SK (2011) Structure of the C‐terminal domain of *Saccharomyces cerevisiae* Nup133, a component of the nuclear pore complex. Proteins 79:1672–1677. 21365675 10.1002/prot.22973 PMC3350809

[59] Seo H‐S , Blus BJ , Jankovic NZ , Blobel G (2013) Structure and nucleic acid binding activity of the nucleoporin Nup157. Proc Natl Acad Sci U S A 110:16450–16455. 24062435 10.1073/pnas.1316607110 PMC3799309

[60] Lin DH , Stuwe T , Schilbach S , Rundlet EJ , Perriches T , Mobbs G , Fan Y , Thierbach K , Huber FM , Collins LN , Davenport AM , Jeon YE , Hoelz A (2016) Architecture of the symmetric core of the nuclear pore. Science 352:aaf1015. 27081075 10.1126/science.aaf1015 PMC5207208

[61] Yoshida K , Seo H‐S , Debler EW , Blobel G , Hoelz A (2011) Structural and functional analysis of an essential nucleoporin heterotrimer on the cytoplasmic face of the nuclear pore complex. Proc Natl Acad Sci U S A 108:16571–16576. 21930948 10.1073/pnas.1112846108 PMC3189060

[62] Debler EW , Ma Y , Seo HS , Hsia KC , Noriega TR , Blobel G , Hoelz A (2008) A fence‐like coat for the nuclear pore membrane. Mol Cell 32:815–826. 19111661 10.1016/j.molcel.2008.12.001

[63] Brohawn SG , Leksa NC , Spear ED , Rajashankar KR , Schwartz TU (2008) Structural evidence for common ancestry of the nuclear pore complex and vesicle coats. Science 322:1369–1373. 18974315 10.1126/science.1165886 PMC2680690

[64] Nagy V , Hsia K‐C , Debler EW , Kampmann M , Davenport AM , Blobel G , Hoelz A (2009) Structure of a trimeric nucleoporin complex reveals alternate oligomerization states. Proc Natl Acad Sci U S A 106:17693–17698. 19805193 10.1073/pnas.0909373106 PMC2764879

[65] Brohawn SG , Schwartz TU (2009) Molecular architecture of the Nup84–Nup145C–Sec13 edge element in the nuclear pore complex lattice. Nat Struct Mol Biol 16:1173–1177. 19855394 10.1038/nsmb.1713 PMC3398507

[66] Hsia KC , Stavropoulos P , Blobel G , Hoelz A (2007) Architecture of a coat for the nuclear pore membrane. Cell 131:1313–1326. 18160040 10.1016/j.cell.2007.11.038 PMC2213454

[67] Ahmed FH , Carr PD , Lee BM , Afriat‐Jurnou L , Mohamed AE , Hong N‐S , Flanagan J , Taylor MC , Greening C , Jackson CJ , Baier D , Purschke B , Schmit C , Rawel HM , Knorr D , Bhabha G , Biel JT , et al. (2015) Architecture of the nuclear pore complex coat. Nature 112:423–430.

[68] Apelt L , Knockenhauer KE , Leksa NC , Benlasfer N , Schwartz TU , Stelzl U (2016) Systematic protein–protein interaction analysis reveals inter‐subcomplex contacts in the nuclear pore complex. Mol Cell Proteomics 15:2594–2606. 27194810 10.1074/mcp.M115.054627 PMC4974338

[69] Buchan DWA , Minneci F , Nugent TCO , Bryson K , Jones DT (2013) Scalable web services for the PSIPRED protein analysis workbench. Nucleic Acids Res 41:W349–W357. 23748958 10.1093/nar/gkt381 PMC3692098

[70] Guan T , Kehlenbach RH , Schirmer EC , Kehlenbach A , Fan F , Clurman BE , Arnheim N , Gerace L (2000) Nup50, a nucleoplasmically oriented nucleoporin with a role in nuclear protein export. Mol Cell Biol 20:5619–5630. 10891499 10.1128/mcb.20.15.5619-5630.2000 PMC86026

[71] Makise M , Mackay DR , Elgort S , Shankaran SS , Adam SA , Ullman KS (2012) The Nup153‐Nup50 protein interface and its role in nuclear import. J Biol Chem 287:38515–38522. 23007389 10.1074/jbc.M112.378893 PMC3493896

[72] Walther TC , Fornerod M , Pickersgill H , Goldberg M , Allen TD , Mattaj IW (2001) The nucleoporin Nup153 is required for nuclear pore basket formation, nuclear pore complex anchoring and import of a subset of nuclear proteins. EMBO J 20:5703–5714. 11598013 10.1093/emboj/20.20.5703 PMC125666

[73] Greber UF , Senior A , Gerace L (1990) A major glycoprotein of the nuclear pore complex is a membrane‐spanning polypeptide with a large lumenal domain and a small cytoplasmic tail. EMBO J 9:1495–1502. 2184032 10.1002/j.1460-2075.1990.tb08267.x PMC551841

[74] Wozniak RW , Blobel G , Rout MP (1994) POM152 is an integral protein of the pore membrane domain of the yeast nuclear envelope. J Cell Biol 125:31–42. 8138573 10.1083/jcb.125.1.31 PMC2120016

[75] Rasala BA , Orjalo AV , Shen Z , Briggs S , Forbes DJ (2006) ELYS is a dual nucleoporin/kinetochore protein required for nuclear pore assembly and proper cell division. Proc Natl Acad Sci U S A 103:17801–17806. 17098863 10.1073/pnas.0608484103 PMC1635652

[76] Bilokapic S , Schwartz TU (2012) Molecular basis for Nup37 and ELY5/ELYS recruitment to the nuclear pore complex. Proc Natl Acad Sci U S A 109:15241–15246. 22955883 10.1073/pnas.1205151109 PMC3458321

[77] Rasala BA , Ramos C , Harel A , Forbes DJ (2008) Capture of AT‐rich chromatin by ELYS recruits POM121 and NDC1 to initiate nuclear pore assembly. Mol Biol Cell 19:3982–3996. 18596237 10.1091/mbc.E08-01-0012 PMC2526682

[78] Lobley A , Sadowski MI , Jones DT (2009) pGenTHREADER and pDomTHREADER: new methods for improved protein fold recognition and superfamily discrimination. Bioinformatics 25:1761–1767. 19429599 10.1093/bioinformatics/btp302

[79] Creevey CJ , Mcinerney JO (2017) Clann: investigating phylogenetic information through supertree analyses. Bioinformatics 21:390–392. 10.1093/bioinformatics/bti020 15374874

[80] Zhong Q , Pevzner SJ , Hao T , Wang Y , Mosca R , Taipale M , Tas M , Fan C , Yang X , Haley P , Murray R , Mer F , Gebreab F , Tam S , Macwilliams A , Dricot A , Reichert P , Santhanam B , Ghamsari L , Calderwood MA , Rolland T , Charloteaux B , Lindquist S , Barabasi AL , Hill DE , Aloy P , Cusick ME , Xia Y , Roth FP , Vidal M (2016) An inter‐species protein–protein interaction network across vast evolutionary distance. Mol Syst Biol 12:1–19. 10.15252/msb.20156484 PMC4848758 27107014

[81] Mosalaganti S , Kosinski J , Albert S , Schaffer M , Strenkert D , Salomé PA , Merchant SS , Plitzko JM , Baumeister W , Engel BD , Beck M (2018) In situ architecture of the algal nuclear pore complex. Nat Commun 9:2361. 29915221 10.1038/s41467-018-04739-y PMC6006428

[82] Kim SJ , Fernandez‐Martinez J , Nudelman I , Shi Y , Zhang W , Raveh B , Herricks T , Slaughter BD , Hogan JA , Upla P , Chemmama IE , Pellarin R , Echeverria I , Shivaraju M , Chaudhury AS , Wang J , Williams R , Unruh JR , Greenberg CH , Jacobs EY , Yu Z , de la Cruz MJ , Mironska R , Stokes DL , Aitchison JD , Jarrold MF , Gerton JL , Ludtke SJ , Akey CW , Chait BT , Sali A , Rout MP (2018) Integrative structure and functional anatomy of a nuclear pore complex. Nature 555:475–482. 29539637 10.1038/nature26003 PMC6022767

[83] Jones DT (1999) Protein secondary structure prediction based on position‐specific scoring matrices. J Mol Biol 292:195–202. 10493868 10.1006/jmbi.1999.3091

[84] Liu W , Xie Y , Ma J , Luo X , Nie P , Zuo Z , Lahrmann U , Zhao Q , Zheng Y , Zhao Y , Xue Y , Ren J (2015) IBS: an illustrator for the presentation and visualization of biological sequences. Bioinformatics 31:3359–3361. 26069263 10.1093/bioinformatics/btv362 PMC4595897

[85] Pei J , Kim BH , Grishin NV (2008) PROMALS3D: a tool for multiple protein sequence and structure alignments. Nucleic Acids Res 36:2295–2300. 18287115 10.1093/nar/gkn072 PMC2367709

[86] Sukumaran J , Holder MT (2017) DendroPy: a python library for phylogenetic computing. Bioinformatics 26:1569–1571. 10.1093/bioinformatics/btq228 20421198

[87] Xia X (2013) DAMBE5: a comprehensive software package for data analysis in molecular biology and evolution. Mol Biol Evol 30:1720–1728. 23564938 10.1093/molbev/mst064 PMC3684854

[88] Yang Z (2017) PAML 4: phylogenetic analysis by maximum likelihood. Mol Biol Evol 24:1586–1591. 10.1093/molbev/msm088 17483113

[89] Xu B , Yang Z (2013) PAML X: a graphical user interface for PAML. Mol Biol Evol 30:2723–2724. 24105918 10.1093/molbev/mst179

[90] Pettersen EF , Goddard TD , Huang CC , Couch GS , Greenblatt DM , Meng EC , Ferrin TE (2004) UCSF chimera – a visualization system for exploratory research and analysis. J Comput Chem 25:1605–1612. 15264254 10.1002/jcc.20084

