# Supplementary material for: Evolutionary divergence of the nuclear pore complex from fungi to metazoans
Source: Protein Sci. 2018 Dec 24;28(3):571–86. doi: 10.1002/pro.3558 (PMC6371224; doi:10.1002/pro.3558)
Supplement: Supplementary file 1 — Appendix S1: Supplementary Information. [file PRO-28-571-s001.docx]

**Supplementary data**

**Evolutionary divergence of the nuclear pore complex from fungi to metazoans**

**Kriti Chopra^1^, Shrankhla Bawaria^1^, and Radha Chauhan^1*^**

**Author details**

^1^ National Center for Cell Science, S.P Pune University, Pune-411007, Maharashtra, India

Email Addresses: Kriti Chopra: [kriti@nccs.res.in](mailto:kriti@nccs.res.in)

Shrankhla Bawaria: [shrankhlabawaria@gmail.com](mailto:shrankhlabawaria@gmail.com)

Radha Chauhan: [radha.chauhan@nccs.res.in](mailto:radha.chauhan@nccs.res.in)

***Corresponding Author**:

Dr. Radha Chauhan, Scientist ‘E’, National Centre for Cell Science, S.P. Pune University Campus, Ganeshkhind, Pune 411007, Maharashtra, India.

Email: [radha.chauhan@nccs.res.in](mailto:radha.chauhan@nccs.res.in)

Phone: +91-20-25708255

**Running title: Divergent evolution of the Nuclear Pore Complex**

**Table S1: Details of compositional difference among species from previous reports**

| **Species Name** | **Number of Nups reported** | **Homologous Nups which are Absent** | **Additional Nups reported** | **Reference Number** |
| --- | --- | --- | --- | --- |
| *Aspergillus nidulans* | **26** | scNup60, scNup1(nuclear basket) scPOM34 and scNup53(membrane binding) |  | 14 |
| *Schizosaccharomyces pombe* | **31** | None | Two paralogues of Nup133 are reported | 15 |
| *Chaetomium thermophilum* | **29** | scNup1 and scNup60 (nuclear basket) | Nup152, Nup56, and POM33 | 16 |
| *Arabidopsis thaliana* | **27** | hNup358, Nup37(cytoplasmic); hNup153(nuclear basket); hPOM121 and hNDC1(Pore membrane); hNup188(adaptor) |  | **17** |
| *Caenorhabditis elegans* | **20** | hALADIN, hCG1, hNup37, hNup43, hNup358, hNup88 (cytoplasmic); hNup188 (adaptor) and hPOM121(pore membrane) |  | **18** |
| *Trypanosoma brucei* | **27** | hNup358, hCG1, hNup214, and Seh1 |  | **19** |
| *Tetrahymena thermophila* | **28** | hNup358, hCG1, Gle1, hNup43, hNup37, ELYS and hALADIN (cytoplasmic side) and hNup35(adaptor ring) |  | **20** |

**Table S2: Details of crystal structures available from different species**

| **Species** | **Name of Protein** | **PDB ID** | **Reference Number** |
| --- | --- | --- | --- |
| ***H. sapiens*** | **Nup98** | **1KO6, 2Q5X, 3MMY** | **27,28,29** |
|  | **Nup358** | **1Z5S, 4I9Y, 3UIP, 4GA0** | **30,31,32,33** |
|  | **Nup50** | **3TJ3** | **34** |
|  | **TPR** | **1W3B** | **35** |
|  | **Nup214** | **3FMO, 2OIT, 3FHC** | **36, 37,38** |
|  | **Nup133** | **1XKS** | **39** |
|  | **Nup43** | **4I79** | **40** |
|  | **Nup133-Nup107** | **3CQC, 3I4R** | **48,49** |
| ***M. musculus*** | **Nup50** | **2C1M** | **41** |
|  | **Nup35** | **1WWH** | **42** |
|  | **ELYS** | **4I0O** | **43** |
| ***R. norvegius*** | **Nup62** | **5H1X** | **44** |
|  | **Nup54** | **4J3H** | **45** |
|  | **Nup58** | **2OSZ** | **46** |
|  | **Nup54-Nup62** | **3T97** | **50** |
|  | **Nup54-Nup58** | **3T98** | **50** |
| ***X. leavis*** | **Nup54** | **5C2U** | **47** |
|  | **Nup54-Nup58-Nup62** | **5C3L** | **47** |
| ***S. cerevisiae*** | **Nup192** | **4IFQ** | **51** |
|  | **Nup159** | **1XIP** | **52** |
|  | **Nic96** | **2QX5, 2RFO** | **53,54** |
|  | **Nup120** | **3HXR, 3F7F** | **55,56** |
|  | **Nup170** | **3I5P, 3I5Q** | **48** |
|  | **Nup145** | **3KEP** | **57** |
|  | **Nup133** | **3KFO** | **58** |
|  | **Nup157** | **4MHC** | **59** |
|  | **Nup82-Nup116-Nup159** | **3PBP** | **61** |
|  | **Nup85-Seh1** | **3F3F** | **62,63** |
|  | **Sec13-Nup145C** | **3BG0** | **66** |
|  | **Nup84-Nup145C-Sec13** | **3JRO, 3IKO** | **64,65** |
|  | **Nup84-Nup145C-Sec13-Nup120** | **4XMN** | **67** |
| ***C. thermophilum*** | **Nup170** | **5HB1** | **60** |
|  | **Nic96** | **5HB2** | **60** |
|  | **Nup192** | **5HB4** | **60** |
|  | **Nup145N** | **5HB5** | **60** |
|  | **Nup53** | **5HB8** | **60** |
|  | **Nup57** | **5CWT** | **6** |
|  | **Nup188** | **5CWU** | **6** |
|  | **Nup170-Nup53** | **5HAX** | **60** |
|  | **Nup170-Nup145** | **5HB0** | **60** |
|  | **Nic96-Nup53** | **5HB3** | **60** |
|  | **Nup82-Nup159-Nup145N** | **5CWW** | **6** |
|  | **Nsp1-Nup57-Nup49-Nic96** | **5CWS** | **6** |

**Methodology details**

**Homology search**

*H. sapiens* Nup sequences were used as a query to fetch sequences from other organisms using online server jackhmmer, using Uniport database reference proteome. Jackhmmer is an iterative search tool against the protein database. The first iteration is a phmmer search against the database selected. The next iteration uses the profile built from the results of the first iteration to search for distant homologs. For every subsequent iteration, a new profile is built based on the results of the previous iteration. The iterations on the server were run till the positive hit of the same protein from lower eukaryotic organism was obtained *(Saccharomyces cerevisiae* S288c/ *Cheatomium thermophilum* DSM1495). Sequence dataset for each nucleoporin was downloaded from the iteration where the search was stopped.

For Nups present exclusively in higher or lower eukaryotes, the jackhmmer search was performed only once. For Nups containing FG repeat regions or unstructured regions (Nup58, Nup54, Nup62, Nup42, Nup50, Nup98, Nup35, Nup214, and Nup358), jackhmmer search was performed after removing these low complexity region sequences. Details of all these searches are listed in Table S3(A-F). The e-value cutoff was taken as 4e-04. The Uniport id of nucleoporins from *H. sapiens*, *S. cerevisiae,* and *C. thermophilum* are listed in Table S4

Additionally, to analyze the % identity and % similarity for homologs of *H. sapiens* with *S. cerevisiae* and *C. thermophilum* Nups, Needleman-Wunsch (global) alignments were generated using EMBOSS Needle program. The values obtained from this analysis are listed in Table S5

**Multiple Sequence Alignments**

The multiple sequence alignments were created using the online server PROMALS3D^1^, which is an HMM and secondary structure-based multiple sequence alignment tool. The alignments were edited in Jalview desktop applet^2^ and were analyzed and modified with respect to the secondary structure prediction of the human nucleoporin sequence. For making reference secondary structure for the alignment, Jnet prediction server^3^ was used through Jalview Desktop applet. Since two different tools were used to predict the secondary structures, a consensus was analyzed between prediction from psipred and jnet while editing the alignments.

The FG repeat regions and unstructured regions were removed taking *H. sapiens* sequences as a reference in the multiple sequence alignment. Since the number of sequences for the nucleoporins was large, a representative dataset was extracted from the PROMALS3D output. PROMALS3D workflow is defined such that it aligns the similar sequences as an initial filter and selects representative sequences. These representative sequences are then used for PSI-BLAST and PSIPRED analysis. Based on the profile-profile HMM and sequence-structure alignments, multiple sequence alignments for the representative sequences are generated and then merged with the input sequences creating the complete alignment. These representative sequences were extracted to create a smaller dataset for processing the phylogenetic analysis Since the number of input sequences varied for each Nup owing to the compositional differences across species, the number of representative sequences were also different. However, the maximum limit of representative sequences for PROMALS3D and hence the representative dataset did not exceed 80 for each Nup.

**Domain Prediction**

Domains for all nucleoporins from *H. sapiens*, *S. cerevisiae,* and *C. thermophilum* were analyzed using hmmscan on the HMMER online server^4^. The reference database used for performing this search was PFAM. The e-value cutoff was taken as 0.01. Representative images of the domains analyzed were created in IBS illustrator^5^. The details of e-values obtained from HMMSCAN are listed in Table S6.

**Table S3A: HMM search results when *S. cerevisiae* is used as a query and *H. sapiens* and *C. thermophilum* as subjects**

| **Query** | **Subject** | **Iteration/e-value** | **Subject** | **Iteration/e-value** |
| --- | --- | --- | --- | --- |
| **(*S. cerevisiae*)** | **(*H. sapiens*)** |  | **(*C. thermophilum*)** |  |
| Nup145 | Nup98-96 | 1/ 3.2e-18; 0.13 | Nup145 | 1/ 5.5e-20 |
| Nup116 |  | 1/ 0.00027; 3.2e-12 |  | 1/ 3e-08; 7.7 |
| Nup100 |  | 1/ 0.00024 |  | 1/ 0.017; 1.9e-15 |
| Nup57 | Nup54 | 2/ 5.9e-34 | Nup57 | 1/ 8.3e-26 |
| Nup49 | Nup58 | 2/ 1.2e-12 | Nup49 | 1/ 2.8e-15 |
| Nsp1 | Nup62 | 1/ 2.6e-17 | Nsp1 | 1/ 3.7e-29 |
| Nup188 | Nup188 | 2/ 6.8e-14 | Nup188 | 1/ 2.1e-14 |
| Nup192 | Nup205 | 2/ 2.3e-114 | Nup192 | 1/ 0.00016; 2.5e-34 |
| Nup157 | Nup155 | 1/ 6.7e-19;1.6e-15 | Nup170 | 1/ 7.1e-96 |
| Nup170 |  | 1/ 4.4e-25;2.8e-26 | Nup170 | 1/ 9.3e-34;0.1;1.8e-70 |
| Nup53 | Nup35 | 2/ 0.0016 | Nup53 | 3/ 4.4e-09 |
| Nup59 |  | 2/ 0.21 |  | 4/ 4.9e-06 |
| Nic96 | Nup93 | 1/ 6e-54 | Nic96 | 1/ 3.3; 1.5e-77; 6.2e-09 |
| Nup82 | Nup88 | 2/ 6e-5; 0.0082 | Nup82 | 2/ 1.6e-16; 0.0003 |
| Nup133 | Nup133 | 2/ 1.9e-34 | Nup133 | 1/ 0.00024 |
| Nup84 | Nup107 | 1/ 3.2e-11 | Nup84 | 1/ 0.00042 |
| Nup120 | Nup160 | 3/ 1.8e-28; 1.2e-09 | Nup120 | 2/ 3.6-37 |
| Nup85 | Nup75 | 2/ 6.7e-17 | Nup85 | 2/ 1.3e-07 |
| Sec13 | Sec13 | 1/ 3.4e-92 | Sec13 | 1/ 5.9e-110 |
| Seh1 | Seh1 | 1/ 2.5e-40; 2.3e-13 | Seh1 | 1/ 3.2e-08; 6.1e-05 |
| Nup2 | Nup50 | 2/ 0.0047; 4.7e-11;  1.50e-27 | Nup56 | 2/ 6.8e-12; 7.3e-06 |
|  |  |  | Nup152 | 2/5.6e- 39 |
| Nup1 | Nup153 | 3/ 5.6e-17 | Not found | Search |
| Mlp1 | Tpr | 1/ 4.4e-19 | Mlp1 | 1/ 0.0042; 1e-50 |
| Mlp2 | Tpr | 2/7.6e-114 | Mlp1 | 1/3e-10 |
| Nup159 | Nup214 | 2/ 1.2e-14 | Nup159 | 2/ 4.9e-33; 4.5e-05 |
| Nup42 | Nup42/CG1 | 3/ 4.5e-05 | Amo1 | 2/ 8.9e-07 |
| Pom33 | TMEM33 | 2/ 2.8e-26 | Pom33 | 1/ 1.5e-27 |
| Pom152 | Not found Search Converged | | Pom152 | 1/ 4.7e-156 |
| Pom34 | Not found Search Converged | | Pom34 | 2/ 0.0008 |
| NDC1 | NDC1 | 2/ 7.3e-07 | NDC1 | 2/ 7.3e-33 |
| Gle1 | Gle1 | 1/ 1.9e-06 | Gle1 | 1/ 4.4e-14 |
| Gle2 | Rae1 | 1/ 3.7e-85 | Gle2 | 1/ 4e-113 |

**Table S3B: HMM search results when *C. thermophilum* is used as a query and *H. sapiens* and *S. cerevisiae* as subjects**

| **Query** | **Subject** | **Iteration/e-value** | **Subject** | **Iteration/e-value** |
| --- | --- | --- | --- | --- |
| **(*C. thermophilum*)** | **(*H. sapiens*)** |  | **(*S. cerevisiae*)** |  |
| Nup145 | Nup98-96 | 1/ 7.7e-19; 0.23; 4.9e-12 | Nup145 | 1/ 7.5e-20 |
|  |  |  | Nup116 | 1/ 3e-13; 1.7e-18 |
|  |  |  | Nup100 | 1/ 0.033; 1.6e-15 |
| Nup57 | Nup54 | 1/ 1.7e-09 | Nup57 | 1/ 1.4e-22 |
| Nup49 | Nup58 | 2/ 4.6e-12 | Nup49 | 1/ 4.5e-11 |
| Nsp1 | Nup62 | 1/ 5e-20 | Nsp1 | 1/ 4.7e-24 |
| Nup188 | Nup188 | 3/ 5.3e-177 | Nup188 | 1/ 6.3e-16 |
| Nup192 | Nup205 | 2/ 1.4e-189 | Nup192 | 1/ 0.00018; 2.4e-33 |
| Nup170 | Nup155 | 1/ 3.9e-33; 1.4e-38 | Nup157 | 1/ 9.6e-96 |
| Nup170 |  |  | Nup170 | 1/ 4.4e-34; 0.04; 1.1e-69 |
| Nup53 | Nup35 | 6/ 0.016 | Nup53 | 3/ 6e-25 |
|  |  |  | Nup59/ASM4 | 3/ 1.4e-16 |
| Nic96 | Nup93 | 1/ 3.2e-38 | Nic96 | 1/ 0.25;3.2e-91 |
| Nup82 | Nup88 | 2/ 0.0012; 9.1e-15 | Nup82 | 2/ 0.00042; 0.0028 |
| Nup133 | Nup133 | 2/ 8.5e-06; 3.7e-35 | Nup133 | 1/ 0.00025 |
| Nup84 | Nup107 | 1/ 6.5e-10 | Nup84 | 1/ 3.9e-05 |
| Nup120 | Nup160 | 1/ 0.0012; 2.2e-08 | Nup120 | 2/ 1.3e-24; 0.86 |
| Nup85 | Nup75 | 2/ 0.013 | Nup85 | 2/ 0.0061 |
| Sec13 | Sec13 | 1/ 5.6e-99 | Sec13 | 1/ 1.3e-110 |
| Seh1 | Seh1 | 1/ 2.3e-05; 0.55 | Seh1 | 1/ 1.4e-08; 4.2e-05 |
| ELYS | ELYS | 2/ 7.7e-16 | Not found | Search converged |
| Nup56 | Nup50 | 3/ 3e-19 | Nup2 | 2/ 1.1e-08 |
| Nup152 | Nup50 | 2/ 4.1e-08 | Nup2 | 2/ 1.7e-29 |
| Mlp1 | Tpr | 1/ 4.4e-61 | Mlp1 | 1/ 0.027; 1.2e-47 |
|  |  |  | Mlp2 | 1/5.7e-07 |
| Nup159 | Nup214 | 2/ 2.7e-140 | Nup159 | 2/ 1.6e-29 |
| Amo1 | Nup42/CG1 | 2/ 4.4e-25 | Nup42 | 2/ 6.0e-07 |
| Pom33 | TMEM33 | 2/ 1.5e-22 | Pom33 | 1/ 1.1e-27 |
| Pom152 | Not Found | Search converged | Pom152 | 1/ 1.9e-156 |
| Pom34 | Not Found | Search converged | Pom34 | 2/ 2.2e-07 |
| NDC1 | NDC1 | 2/ 2.8e-32 | NDC1 | 2/ 2.9e-30 |
| Gle1 | Gle1 | 2/1.1e-09 | Gle1 | 1/7.1e-14 |
| Gle2 | Rae1 | 1/ 2.2e-86 | Gle2 | 1/ 9.2e-113 |

**Table S3C: HMM search results when *H. sapiens* is used as query and *S. cerevisiae* and *C. thermophilum* as subjects**

| **Query** | **Subject** | **Iteration/e-value** | **Subject** | **Iteration/e-value** |
| --- | --- | --- | --- | --- |
| **(*H. sapiens*)** | **(*S. cerevisiae*)** |  | **(*C. thermophilum*)** |  |
| Nup98-96 | Nup145 | 1/ 8.4e-18; 0.055 | Nup145 | 1/ 2e-22; 0.27; 6.9e-12 |
|  | Nup116 | 1/ 6.5e-16 |  |  |
|  | Nup100 | 2/ 2.1e-77 |  |  |
| Nup54 | Nup57 | 2/ 5.6e-30 | Nup57 | 2/ 5e-46 |
| Nup58 | Nup49 | 2/ 1.6e-14 | Nup49 | 2/ 2.5e-17 |
| Nup62 | Nsp1 | 1/ 2.3e-17 | Nsp1 | 1/ 3.9e-22 |
| Nup188 | Nup188 | 3/ 5.1e-59 | Nup188 | 2/ 3.4e-07 |
| Nup205 | Nup192 | 2/ 2e-86 | Nup192 | 2/ 2.7e-208 |
| Nup155 | Nup157 | 1/1.1e-19; 5.6e-15 |  |  |
|  | Nup170 | 1/ 1.8e-25; 1.8e-25 | Nup170 | 1/ 1.1e-32;3.6e-39 |
| Nup35 | Nup53 | 2/ 7.5e-14 | Nup53 | Not found |
|  | Nup59 | 2/ 0.086;0.0029 |  |  |
| Nup93 | Nic96 | 1/ 6.8e-53 | Nic96 | 1/ 6.2e-38 |
| Nup88 | Nup82 | 2/ 1.2e-05;1.2 | Nup82 | 2/ 2e-05; 2.6e-20 |
| Nup133 | Nup133 | 2/ 5.1e-07;1.9e-19 | Nup133 | 2/ 3.8e-11;6.2e-42 |
| Nup107 | Nup84 | 2/ 5.2e-116 | Nup84 | 1/ 5e-10 |
| Nup160 | Nup120 | 4/ 5e-25;0.00052 | Nup120 | 1/ 0.0005;3.1e-10 |
| Nup75 | Nup85 | 2/ 1e-28 | Nup85 | 2/ 0.0017;0.33 |
| Sec13 | Sec13 | 1/ 4.1e-93 | Sec13 | 1/ 1.7e-99 |
| Seh1 | Seh1 | 1/ 1.2e-40; 1.1e-13 | Seh1 | 1/ 3.4e-05 |
| ELYS | Not found Search converged | | ELYS | 2/ 2.2e-26 |
| Nup50 | Nup2 | 2/ 2.3e-06 | Nup56 | 3/ 3e-24 |
|  |  |  | Nup152 | 2/ 1.3e-07 |
| Nup153 | Nup1 | 2/ 9.8-06 | Not found Search converged | |
| Tpr | Mlp1 | 1/ 1.9e-14 | Mlp1 | 1/ 1.2e-58 |
|  | Mlp2 | Not found |  |  |
| Nup214 | Nup159 | 2/ 9.8e-18 | Nup159 | 2/ 3.3e-126 |
| Nup42/CG1 | Nup42 | 3/ 2.2e-08 | Amo1 | 2/0.00019;9.8e-30 |
| TMEM33 | Pom33 | 2/ 1.2e-38 | Pom33 | 2/ 1.1e-43 |
| NDC1 | NDC1 | 2/9.8e-11 | NDC1 | 2/ 2.3e-54 |
| Gle1 | Gle1 | 1/ 6.5e-08 | Gle1 | 1/ 4.4e-14 |
| Rae1 | Gle2 | 1/ 2.6e-85 | Gle2 | 1/ 6e-87 |

**Table3D: HMM search results when only structured regions of FG containing Nups of *S. cerevisiae* are used as a query and *H. sapiens* and *C. thermophilum* as subjects**

| **Query** | **Subject** | **Iteration/e-value** | **Subject** | **Iteration/e-value** |
| --- | --- | --- | --- | --- |
| **(*S. cerevisiae*)** | **(*H. sapiens*)** |  | **(*C. thermophilum*)** |  |
| Nup145 | Nup98-96 | 1/ 9.2e-19 | Nup145 | 1/ 2.2e-19 |
| Nup116 |  | 1/ 2.8e-05 | Nup145 | 1/ 1.2e-16 |
| Nup100 |  | 1/ 2.8e-05 | Nup145 | 1/ 1.2e-16 |
| Nup57 | Nup54 | 2/6e-35 | Nup57 | 1/1.6e-25 |
| Nup49 | Nup58 | 3/ 4.2e-16 | Nup49 | 2/6.8e-18 |
| Nsp1 | Nup62 | 1/7.3e-18 | Nsp1 | 1/3.2e-28 |
| Nup53 | Nup35 | 2/ 0.8 | Nup53 | 3/ 1.1e-07 |
| Nup59 |  | 5/ 1.8e-07 |  |  |
| Nup2 | Nup50 | 2/9.2e-11 | Nup56 | 2/ 0.015 & 1.1e-05 |
|  |  |  | Nup152 | 2/2.4e-18 |
| Nup159 | Nup214 | 2/ 6.3e-16 | Nup159 | 2/ 1.2e-36 |
| Nup42 | >90% sequence is unstructured (FxFG), cannot be used as a query sequence | | | |

**Table S3E: HMM search results when only structured regions of FG containing Nups of *C. thermophilum* are used as a query and *H. sapiens* and *S. cerevisiae* as subjects**

| **Query** | **Subject** | **Iteration/e-value** | **Subject** | **Iteration/e-value** |
| --- | --- | --- | --- | --- |
| **(*C. thermophilum*)** | **(*H. sapiens*)** |  | **(*S. cerevisiae*)** |  |
| Nup145 | Nup98-96 | 1/ 9.1e-20 | Nup145 | 1/ 1.1e-20 |
| Nup145 |  |  | Nup116 | 1/ 9.3e-20 |
| Nup145 |  |  | Nup100 | 1/ 6.4e-17 |
| Nup57 | Nup54 | 1/1.2e-09 | Nup57 | 1/1.2e-16 |
| Nup49 | Nup58 | 4/0.00054 & 4.4 | Nup49 | 2/1.1e-07 |
| Nsp1 | Nup62 | 1/1.2e-25 | Nsp1 | 1/5.2e-28 |
| Nup53 | Nup35 | Not found | Nup53 | 3/ 3.1e-07 |
|  |  |  | Nup59 | 3/ 4.3e-12 |
| Nup56 | Nup50 | 3/2.4e-09 | Nup2 | 2/1.5e-19 |
| Nup152 |  | 2/5.7e-08 | Nup2 | 2/0.022 |
| Nup159 | Nup214 | 2/ 9.9e-90 | Nup159 | Not found |
| Nup42 | Nup42 | 2/ 1.4e-05 | Nup42 | Not found |

**Table S3F: HMM search results when only structured regions of FG containing Nups of *H. sapiens* are used as query and *S. cerevisiae* and *C. thermophilum* as subjects**

| **Query** | **Subject** | **Iteration/e-value** | **Subject** | **Iteration/e-value** |
| --- | --- | --- | --- | --- |
| **(*H. sapiens*)** | **(*S. cerevisiae*)** |  | **(*C. thermophilum*)** |  |
| Nup98-96 | Nup145 | 1/ 3.9e-18;0.027;1.6 | Nup145 | 1/ 6.1e-19;0.13;3.2e-12 |
|  | Nup116 | 1/ 4.1e-08 |  |  |
|  | Nup100 | 1/ 2.8e-05 |  |  |
| Nup54 | Nup57 | 2/2.4e-30 | Nup57 | 1/8.6e-11 |
| Nup58 | Nup49 | 2/2.8e-09 | Nup49 | 2/5.5e-08 |
| Nup62 | Nsp1 | 1/5.5e-18 | Nsp1 | 1/1.1e-26 |
| Nup35 | Nup53 | 2/ 3.6e-08 | Nup53 | Not found |
|  | Nup59 | 2/ 0.058 |  |  |
| Nup50 | Nup2 | 2/1.1e-13 | Nup56 | 3/2.5e-16 |
|  |  |  | Nup152 | 2/8.2e-06 |
| Nup214 | Nup159 | Not found | Nup159 | 1/ 4.7e-15 |
| Nup42 | Nup42 | Not found | Nup42 | 2/ 4.8e-05 |

**Table S4: Nomenclature Translation Table (List of Nucleoporins of *H. sapiens* NPC along with their *S. cerevisiae* and *C. thermophilum* homologs)**

| *H. sapiens* | UniProt ID | *S. cerevisiae* | UniProt ID | *C. thermophilum* | UniProt ID |
| --- | --- | --- | --- | --- | --- |
| Nup98-96 | P52948 | Nup145 | P49687 | Nup145 | G0SAK3 |
|  |  | Nup116 | Q02630 |  |  |
|  |  | Nup100 | Q02629 |  |  |
| Nup54 | Q7Z3B4 | Nup57 | P48837 | Nup57 | G0S0R2 |
| Nup58 | Q9BVL2 | Nup49 | Q02199 | Nup49 | G0S4X2 |
| Nup62 | P37198 | NSP1 | P14907 | NSP1 | G0SBQ3 |
| Nup188 | Q5SRE5 | Nup188 | P52593 | Nup188 | G0SFH5 |
| Nup205 | Q92621 | Nup192 | P47054 | Nup192 | G0S4T0 |
| Nup155 | O75694 | Nup157 | P40064 |  |  |
|  |  | Nup170 | P38181 | Nup170 | G0S7B6 |
| Nup35 | Q8NFH5 | Nup53 | Q03790 | Nup53 | G0S156 |
|  |  | Nup59 | Q05166 |  |  |
| Nup93 | Q8N1F7 | Nic96 | P34077 | Nic96 | G0S024 |
| Nup88 | Q99567 | Nup82 | P40368 | Nup82 | G0S4F3 |
| Nup133 | Q8WUM0 | Nup133 | P36161 | Nup133 | G0S9A7 |
| Nup107 | P57740 | Nup84 | P52891 | Nup84 | G0SER9 |
| Nup160 | Q12769 | Nup120 | P35729 | Nup120 | G0S0E7 |
| Nup75 | Q9BW27 | Nup85 | P46673 | Nup85 | G0SDQ4 |
| Sec13 | P55735 | Sec13 | Q04491 | Sec13 | G0SA60 |
| Seh1 | Q96EE3 | Seh1 | P53011 | Seh1 | G0S450 |
| ELYS | Q8WYP5 |  |  | ELYS | G0S2G1 |
| Nup43 | Q8NFH3 |  |  |  |  |
| Nup37 | Q8NFH4 |  |  |  |  |
| ALADIN | Q9NRG9 |  |  |  |  |
| Nup50 | Q9UKX7 | Nup2 | P32499 | Nup56 | G0S8I1 |
|  |  |  |  | Nup152 | G0SDP9 |
| Nup153 | P49790 | Nup1 | P20676 |  |  |
| TPR | P12270 | MLP1 | Q02455 | MLP1 | G0SA56 |
|  |  | MLP2 | P40457 |  |  |
| Nup214 | P35658 | Nup159 | P40477 | Nup159 | G0SBS8 |
| CG1 | O15504 | Nup42 | P49686 | Nup42 | G0S381 |
| Nup358 | P49792 |  |  |  |  |
| GP210 | Q8TEM1 |  |  |  |  |
|  |  | POM152 | P39685 | Pom152 | G0SB44 |
| Pom121 | Q96HA1 |  |  |  |  |
| TMEM33 | P57088 | POM33 | Q12164 | POM33 | G0S6T0 |
| NDC1 | Q9BTX1 | NDC1 | P32500 | NDC1 | GOS235 |
|  |  | POM34 | Q12445 | POM34 | G0S7R3 |
| GLE1 | Q53GS7 | GLE1 | Q12315 | GLE1 | G0S7F3 |
| RAE1 | P78406 | GLE2 | P40066 | GLE2 | G0SEA3 |

*H. sapiens* NPC is composed of nearly 30 nucleoporins embedded in the complex. This table represents all the 30 nucleoporins along with RNA export proteins from *H. sapiens* along with their homologs in *S. cerevisiae* and *C. thermophilum*. UniProt accession ID is mentioned along with each nucleoporin from these three species The Nups that are unique to a particular species are colored in red.

**Table S5: Percentage identity and percentage similarity for all orthologous pairs of *H. sapiens- S. cerevisiae* Nups and *H. sapiens-C. thermophilum* Nups**

| *H. sapiens* | *S. cerevisiae* | %identity | %similarity | *H. sapiens* | *C. thermophilum* | %identity | %similarity |
| --- | --- | --- | --- | --- | --- | --- | --- |
| Nup62 | Nsp1 | 18.4 | 28.1 | Nup62 | Nsp1 | 31.5 | 43.4 |
| Nup54 | Nup57 | 22.1 | 34.1 | Nup54 | Nup57 | 17.5 | 27.9 |
| Nup58 | Nup49 | 17.3 | 28.5 | Nup58 | Nup49 | 19.2 | 29.1 |
| Nup98 | Nup145 | 19.2 | 31.1 | Nup98 | Nup145 | 26.3 | 38.1 |
|  | Nup116 | 14 | 19.9 |  |  |  |  |
|  | Nup100 | 14.8 | 20.9 |  |  |  |  |
| Nup188 | Nup188 | 17.9 | 32.6 | Nup188 | Nup188 | 15.9 | 26.3 |
| Nup205 | Nup192 | 17.1 | 31.2 | Nup205 | Nup192 | 17.7 | 31.5 |
| Nup155 | Nup157 | 19.8 | 35.4 | Nup155 | Nup170 | 22.5 | 38.6 |
| Nup155 | Nup170 | 19.9 | 35.5 |  |  |  |  |
| Nup35 | Nup53 | 19.4 | 32.7 | Nup35 | Nup53 | 12.9 | 22.2 |
|  | Asm4 | 12.4 | 21.6 |  |  |  |  |
| Nup93 | Nic96 | 22.8 | 44.8 | Nup93 | Nic96 | 19.1 | 34.8 |
| Nup88 | Nup82 | 17.4 | 32.5 | Nup88 | Nup82 | 18.2 | 33.7 |
| Nup133 | Nup133 | 17.1 | 30.8 | Nup133 | Nup133 | 18.5 | 33.3 |
| Nup107 | Nup84 | 17.4 | 33.9 | Nup107 | Nup84 | 17.9 | 33.2 |
| Nup160 | Nup120 | 12.8 | 23.9 | Nup160 | Nup120 | 18.5 | 31.8 |
| Nup75 | Nup85 | 16.8 | 31.5 | Nup75 | Nup85 | 12.2 | 22.7 |
| Sec13 | Sec13 | 48 | 62.8 | Sec13 | Sec13 | 47.6 | 63 |
| Seh1 | Seh1 | 31.3 | 49.9 | Seh1 | Seh1 | 17.3 | 28.3 |
|  |  |  |  | ELYS | ELYS | 3 | 5.4 |
| Nup50 | Nup2 | 16.5 | 25.8 | Nup50 | Nup56 | 19.1 | 32.3 |
|  |  |  |  |  | Nup152 | 9.9 | 13.3 |
| Nup153 | Nup1 | 17.6 | 26.8 |  |  |  |  |
| TPR | MLP1 | 17.9 | 35.1 | TPR | MLP1 | 20.5 | 36.9 |
|  | MLP2 | 16.8 | 32.9 |  |  |  |  |
| Nup214 | Nup159 | 8.9 | 12.8 | Nup214 | Nup159 | 14.5 | 24.5 |
| CG1 | Nup42 | 17.1 | 25.9 | CG1 | Nup42 | 18.4 | 27 |
| TMEM33 | POM33 | 23.5 | 39.4 | TMEM33 | POM33 | 17.3 | 35.2 |
| NDC1 | NDC1 | 18.6 | 32.2 | NDC1 | NDC1 | 18.3 | 33.3 |
| GLE1 | GLE1 | 18.7 | 33.2 | GLE1 | GLE1 | 17.4 | 29.6 |
| RAE1 | GLE2 | 40.7 | 56.7 | RAE1 | GLE2 | 43.9 | 57.9 |

**Table S6: Pfam domain results for all Nups from *H. sapiens, S. cerevisiae,* and *C. thermophilum***

| ***H. sapiens*** | | | ***S. cerevisiae*** | | | ***C. thermophilum*** | | |
| --- | --- | --- | --- | --- | --- | --- | --- | --- |
| **Protein** | **Pfam ID** | **e-value** | **Protein** | **Pfam ID** | **e-value** | **Protein** | **Pfam ID** | **e-value** |
| Nup98 | PF04096.13 | 1.50E-40 | Nup145 | PF04096.13 | 3.60E-43 | Nup145 | PF04096.13 | 8.20E-58 |
|  | PF12110.7 | 4.10E-99 |  | PF12110.7 | 6.70E-37 |  | PF12110.7 | 1.30E-42 |
|  |  |  | Nup116 | PF04096.13 | 6.80E-42 |  |  |  |
|  |  |  | Nup100 | PF04096.13 | 3.10E-37 |  |  |  |
| Nup54 | PF13874.5 | 9.50E-45 | Nup57 | PF13874.5 | 4.40E-36 | Nup57 | PF13874.5 | 6.00E-41 |
| Nup58 | PF15967.4 | 3.60E-307 | Nup49 | PF13634.5 | 1.80E-09 | Nup49 | PF13634.5 | 4.20E-13 |
| Nup62 | PF05064.12 | 1.40E-37 | Nsp1 | PF05064.12 | 2.30E-41 | Nsp1 | PF05064.12 | 3.60E-41 |
| Nup188 | PF10487.8 | 5.30E-216 | Nup188 | PF10487.8 | 7.80E-264 | Nup188 | PF10487.8 | 3.30E-28 |
| Nup205 | PF11894.7 | 0.00E+00 | Nup192 | PF11894.7 | 0.00E+00 | Nup192 | PF11894.7 | 0.00E+00 |
| Nup155 | PF08801.10 | 7.30E-107 | Nup157 | PF08801.10 | 4.10E-119 | Nup170 | PF08801.10 | 2.10E-102 |
|  | PF03177.13 | 2.50E-20 |  | PF03177.13 | 1.90E-85 |  | PF03177.13 | 3.40E-50 |
|  |  |  | Nup170 | PF08801.10 | 2.20E-139 |  |  |  |
|  |  |  |  | PF03177.13 | 2.40E-121 |  |  |  |
| Nup35 | PF05172.12 | 2.50E-26 | Nup53 | PF05172.12 | 5.00E-39 | Nup53 | PF05172.12 | 1.80E-03 |
|  |  |  | Nup59 | PF05172.12 | 3.70E-42 |  |  |  |
| Nup93 | PF04097.13 | 3.70E-196 | Nic96 | PF04097.13 | 4.70E-199 | Nic96 | PF04097.13 | 6.20E-229 |
| Nup88 | PF10168.8 | 2.4e-310 | Nup82 | No domain found |  | Nup82 | No domain found |  |
| Nup133 | PF03177.13 | 7.10E-23 | Nup133 | PF08801.10 | 1.70E-76 | Nup133 | PF08801.10 | 3.00E-91 |
|  |  |  |  |  |  |  | PF03177.13 | 1.10E-174 |
| Nup107 | PF04121.12 | 2.80E-215 | Nup84 | PF04121.12 | 6.60E-177 | Nup84 | PF04121.12 | 7.70E-193 |
| Nup160 | PF11715.7 | 2.90E-122 | Nup120 | No domain found |  | Nup120 | PF11715.7 | 1.20E-164 |
| Nup75 | PF07575.12 | 2.10E-178 | Nup85 | PF07575.12 | 3.00E-189 | Nup85 | PF07575.12 | 2.10E-07 |
| Sec13 | PF00400.31 | 6.50E-04 | Sec13 | PF00400.31 | 3.10E-05 | Sec13 | PF00400.31 | 1.60E-05 |
| Seh1 | PF00400.31 | 3.10E-04 | Seh1 | PF00400.31 | 6.30E-05 | Seh1 | PF00400.31 | 6.00E-04 |
| ELYS | PF16687.4 | 1.40E-307 |  |  |  | ELYS | PF13934.5 | 1.10E-69 |
|  | PF13934.5 | 5.60E-54 |  |  |  |  |  |  |
| Nup43 | PF00400.31 | 3.90E-03 |  |  |  |  |  |  |
| Nup37 | PF00400.31 | 6.80E-05 |  |  |  |  |  |  |
| Aladin | PF00400.31 | 2.00E-04 |  |  |  |  |  |  |
| Nup50 | PF08911.10 | 5.60E-20 | Nup2 | PF08911.10 | 4.80E-14 | Nup56 | PF00638.17 | 5.20E-05 |
|  | PF00638.17 | 2.60E-11 |  | PF00638.17 | 4.50E-44 | Nup152 | PF00638.17 | 3.30E-13 |
| Nup153 | PF08604.9 | 2.30E-235 | Nup1 | No domain found |  |  |  |  |
| TPR | PF07926.11 | 2.60E-34 | MLP1 | PF07926.11 | 5.00E-38 | MLP1 | PF07926.11 | 5.00E-38 |
| Nup214 | No domain found |  | Nup159 | PF16755.3 | 6.00E-171 | Nup159 | No domain found |  |
| CG1 | No domain found |  | Nup42 | No domain found |  | Nup42 | PF00642.23 | 1.40E-06 |
| Nup358 | PF006381.17 | 6.20E-52 |  |  |  |  |  |  |
|  | PF00641.17 | 6.60E-12 |  |  |  |  |  |  |
|  | PF12185.7 | 5.60E-27 |  |  |  |  |  |  |
|  | PF00160.20 | 1.80E-40 |  |  |  |  |  |  |
| GP210 | PF023681.17 | 5.00E-16 |  |  |  |  |  |  |
|  |  |  | Pom152 | No domain found |  | Pom152 | No domain found |  |
| POM121 | PF15229.5 | 1.60E-109 |  |  |  |  |  |  |
|  |  |  | Pom34 | PF08058.10 | 2.60E-36 | Pom34 | PF08058.10 | 6.90E-26 |
| TMEM33 | PF03661.12 | 1.20E-95 | Pom33 | PF03661.12 | 2.90E-24 | Pom33 | PF03661.12 | 3.20E-30 |
| NDC1 | PF09531.9 | 2.00E-135 | NDC1 | PF09531.9 | 3.10E-107 | NDC1 | PF09531.9 | 2.30E-187 |
| GLE1 | PF07817.12 | 5.40E-88 | Gle1 | PF07817.12 | 7.30E-87 | Gle1 | PF07817.12 | 1.40E-58 |
| RAE1 | PF00400.31 | 4.50E-04 | Gle2 | PF00400.31 | 1.60E-05 | Gle2 | PF00400.31 | 0.0074 |

**
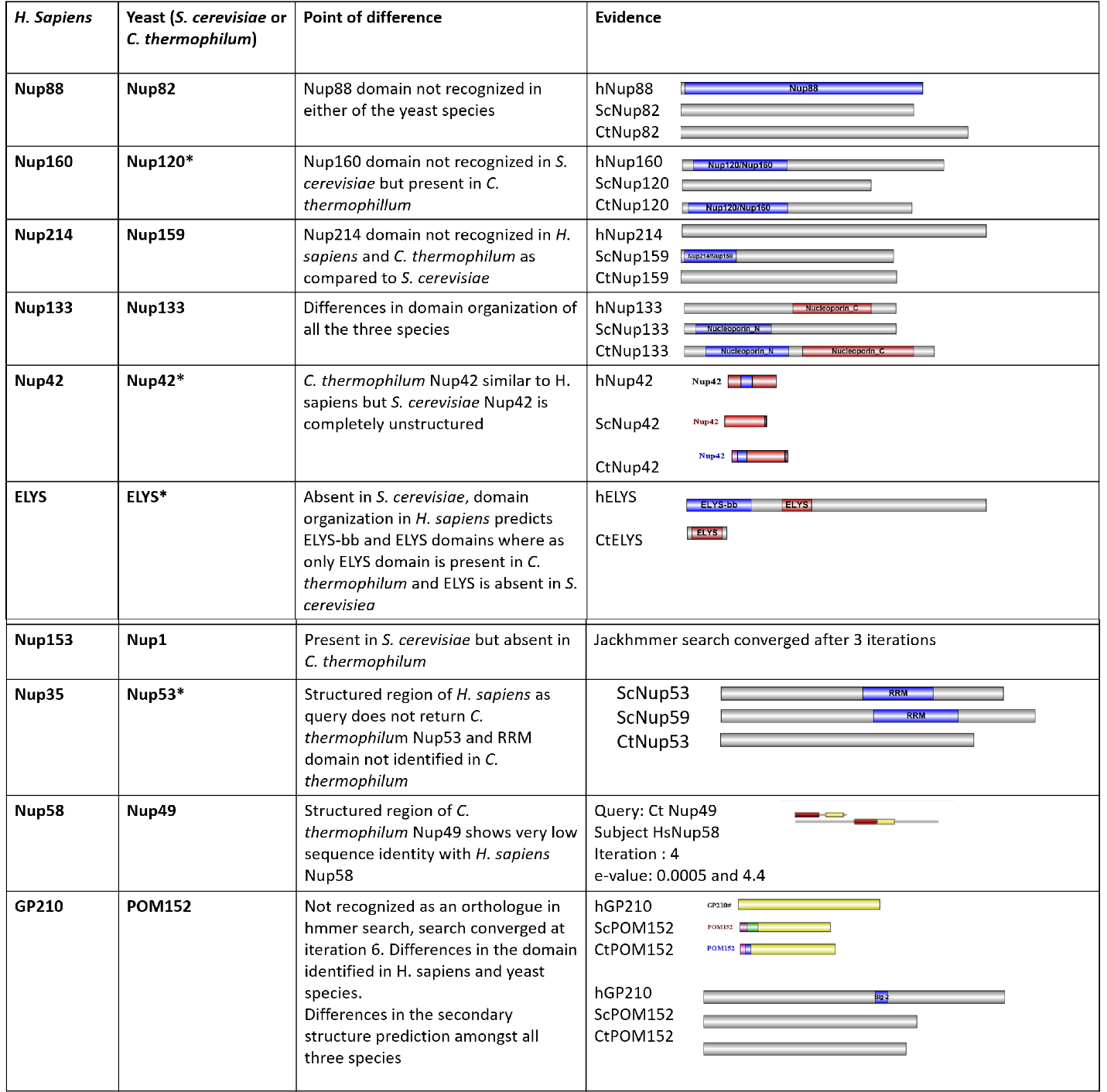
 Figure S1: Difference observed at sequence/ secondary structure/ domain organization level between *H. sapiens* and yeast species (*S. cerevisiae* and *C. thermophilum*) as well as between the yeast species**

There are 10 specific instances of dissimilarity at sequence/ secondary structure or domain organization level between nucleoporins of human and yeast species and 4 (marked with *) showed dissimilarity between the fungal species. The schematic representations for Nup88, Nup160, Nup214, Nup133, ELYS, yNup53, and Gp210 represent their domain organization. Secondary structure prediction is depicted for Nup42 and Gp210. Hmmer hit position details are shown for Nup58

**
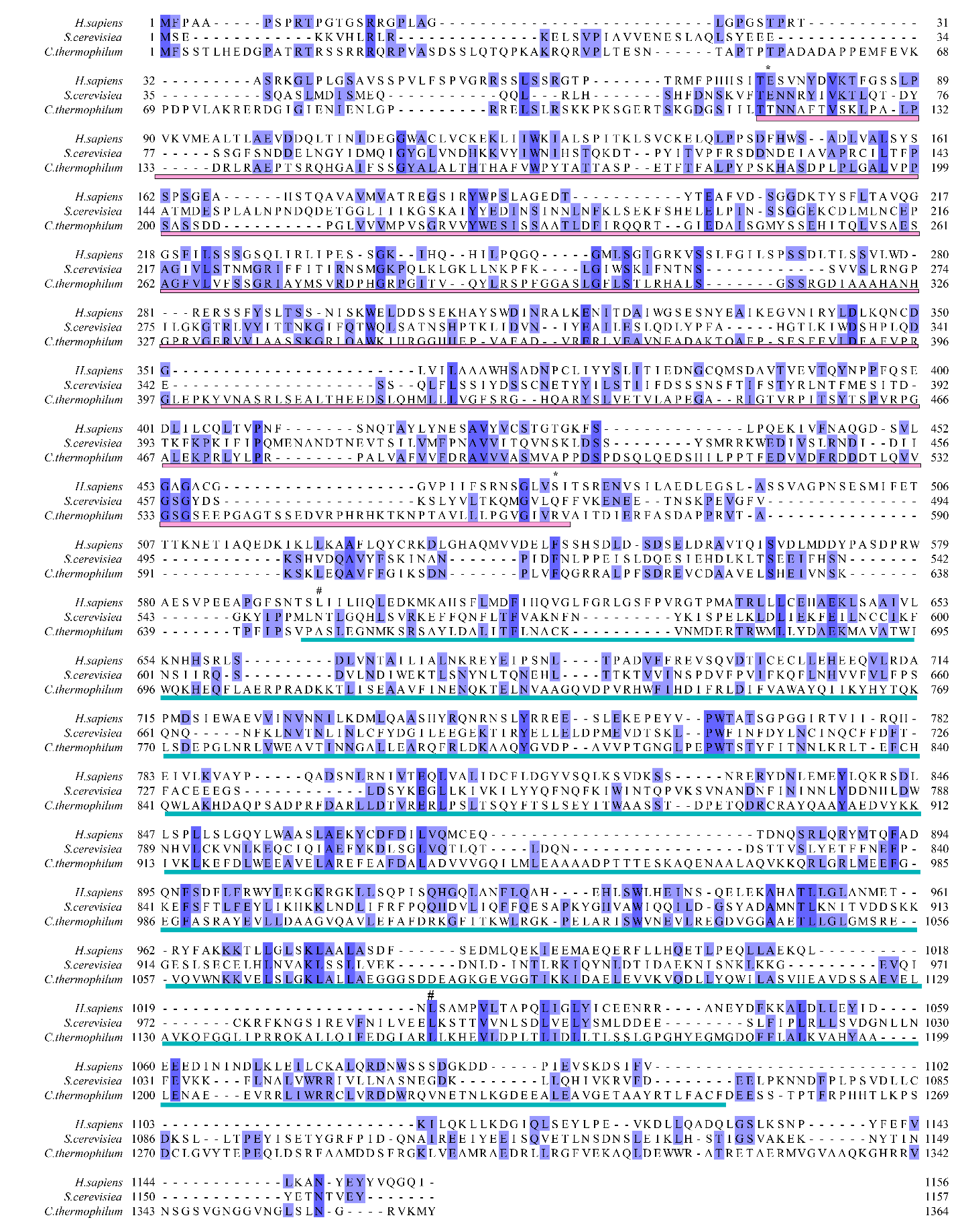
**

**Figure S2: Structure-guided multiple sequence alignment of representative species from the three classes of Nup133**

A structure-guided multiple sequence alignment of *H. sapiens* representative of containing only Nucleoporin_C domain, *S. cerevisiae* representative of containing only Nucleoporin_N domain and *C. thermophilum* representative of containing both domains. The red bar represents the predicted Nucleoporin_N domain in *C. thermophilum* and * bounds represent predicted Nulceoporin_N domain in *S. cerevisiae*. The cyan bar represents the Nucleoporin_C domain predicted in *C. thermophilum* and # bound represent predicted Nucleoporin_C domain in *H. sapiens*

**Nup133 evolutionary pressure analysis**

The total dataset was divided three categories viz sequences having only Nucleoporin_C domain (*H. sapiens* was kept as reference), sequences having only Nucleoporin_N domain *(S. cerevisiae* was kept as reference) and sequences having both Nucleoporin_N and Nucleoporin_C domain (*C. thermophilum* was kept as reference). 25 sequences formed group 1 which predicted to have only Nucleoporin_C domain with *H. sapiens* as a representative sequence. 16 sequences formed group 2 which predicted to have only Nucleopotin_N domain with *S. cerevisiae* as a representative sequence. 36 sequences formed group 3 which predicted to have both the domains with *C. thermophilum* as a representative sequence. These three groups were analyzed separately to calculate the Bayes Empirical Bayes (BEB) probability with the PAML software package^6^ of a residue being under purifying (dN/dS <1), neutral (dN/dS=1) or positive (dN/dS>1) selection pressure.

On comparing the Nucleoporin_N domain predicted for *S. cerevisiae* and *C. thermophilum* and the corresponding region in *H. sapiens* (Figure S3), it was observed that 14% residues are under purifying selection in *S. cerevisiae* as compared to 7% in *C. thermophium* and 5% in *H. sapiens* (Figure S3) Interestingly, about 55% residues in *C. thermophilum* and 29% residues in *H. sapiens* of Nucleoporin_N domain are under positive selection pressure. Thus, it is evident that Nucleoporin_N domain is most conserved for group 2 species (*S. cerevisiae* as representative), under positive selection in group 3 (*C. thermophilum* as representative) and loss of domain is observed for group 1 (*H. sapiens* as representative).

Similarly, on comparing Nucleoporin_C domain predicted for *H. sapiens* and *C. thermophilum* and the corresponding region in *S. cerevisiae* (Figure S3), it was observed that 11% residues are under purifying selection pressure in *H. sapiens* as compared to 14% residues in *S. cerevisiae* and 9% in *C. thermophilum* (Figure S3). Additionally, about 52% residues in *C. thermophilum* and 27% residues in *S. cerevisiae* of Nucleoporin_C domain are under positive selection pressure.

**
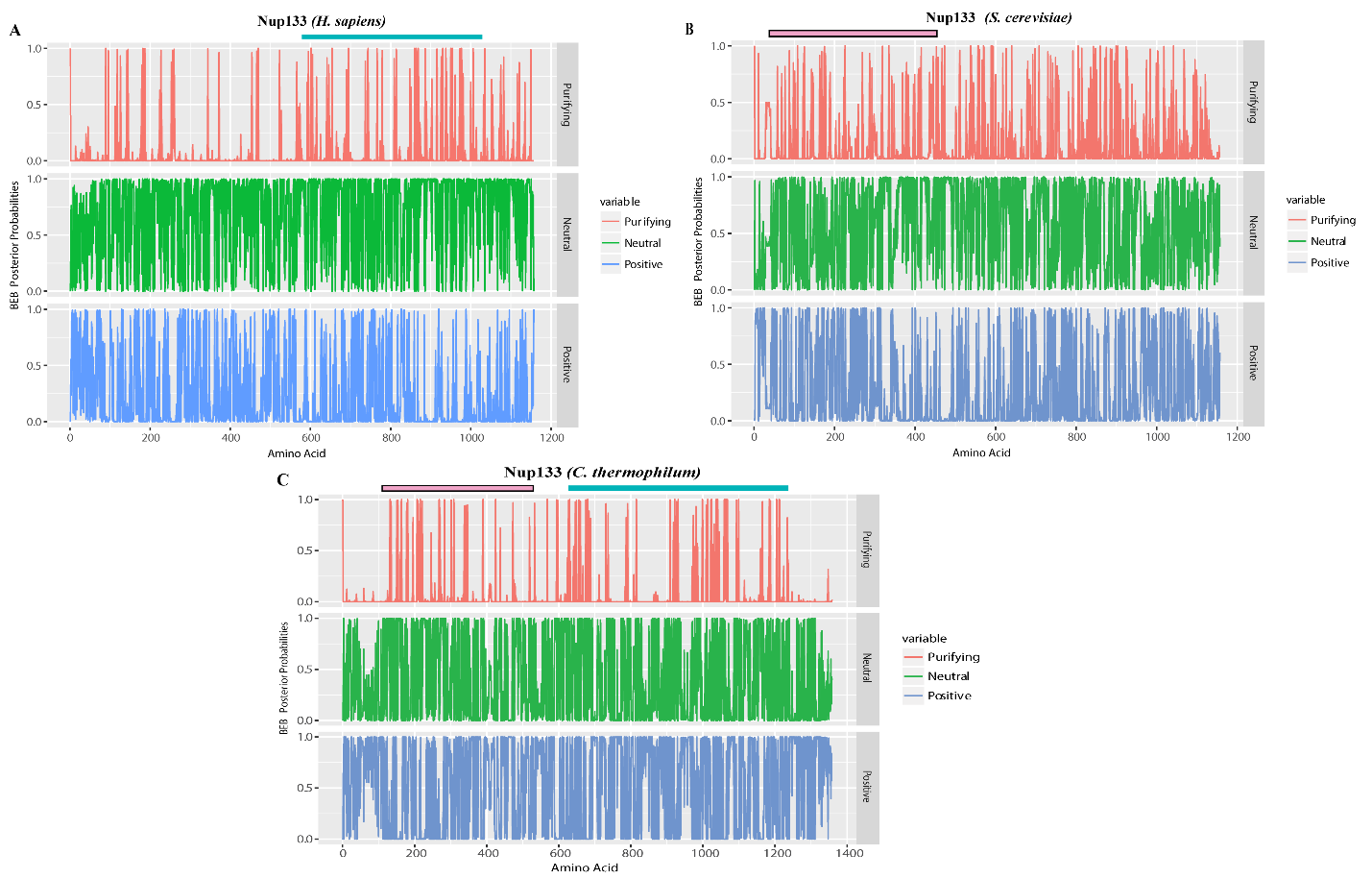
 Figure S3: Evolutionary pressure analysis on the three classes of Nup133**

BEB probability obtained after dN/dS (ω) calculation is depicted for the three classes of Nup133. A) *H. sapiens* Nup133 was taken as representative sequence for the class of species predicted to have only Nucleoporin_C domain.B) *S. cerevisiae* Nup133 was taken as representative sequence for the class of species predicted to have only Nucleoporin_N domain. C) *C. thermophilum* as one for those predicted to have both the domains. The BEB of residue positions under purifying selection pressure (ω<1) are colored as red, neutral selection pressure (ω=1) positions are colored as green and positive selection pressure (ω>1) are colored as blue. The pink bar represents the predicted region of the Nulceoporin_N domain in *C. thermophilum* (118-571) and in *S. cerevisiae* (62-476). The cyan bar represents the predicted region of the Nucleoporin_C domain in *C. thermophilum* (646-1252) and in *H. sapiens* (594-1020).

**
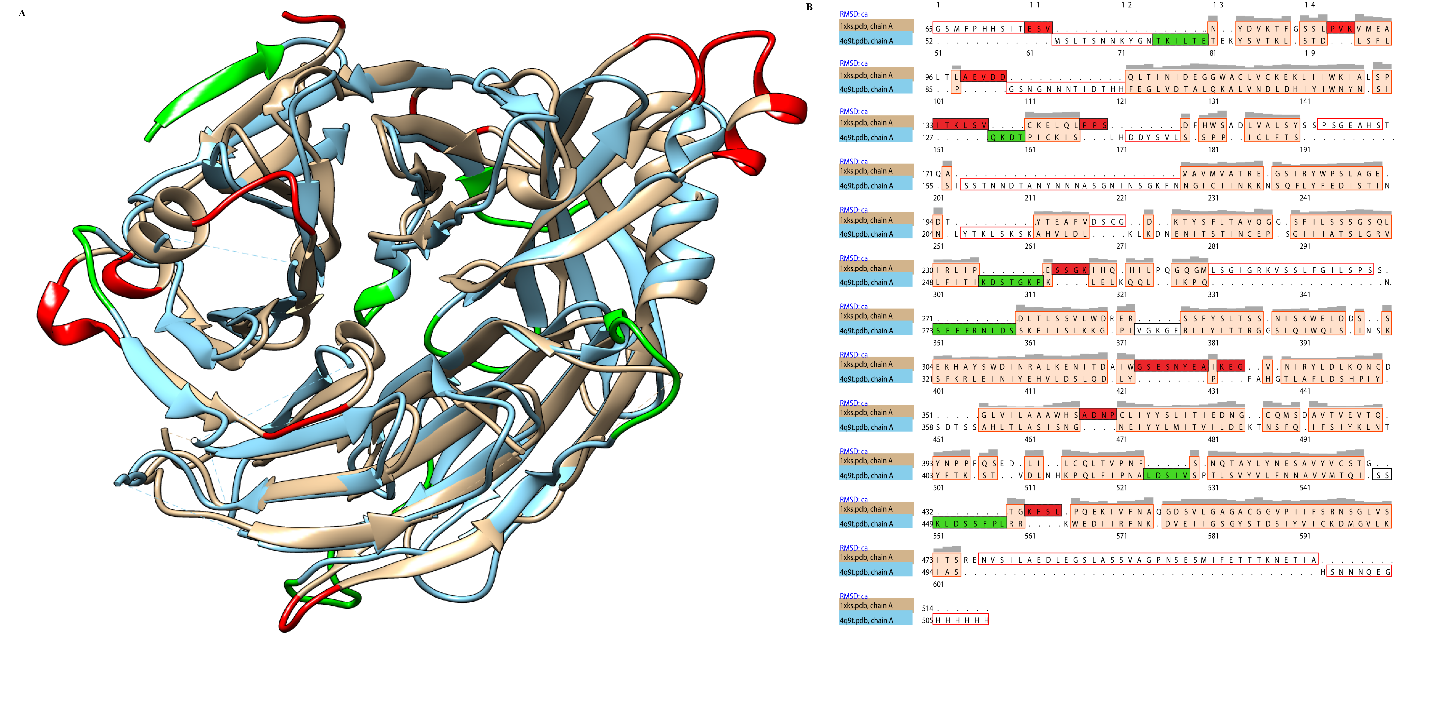
**

**Figure S4: Superimposition of the N-terminal domain of Nup133 from known crystal structures**

A. Superimposition of *H. sapiens* Nup133 N-terminal domain (PDB ID: 1XKS) shown in tan with *V. polyspora* Nup133N-terminal domain (PDB ID: 4Q9T) shown in cyan. The regions of 1XKS which do not superimpose are colored red and those of 4Q9T are colored in green.

B. Structure-guided alignment of the 1XKS and 4Q9T. The residues which do not superimpose in the structure are also colored in red and green respectively. There are 303 fully populated columns as opposed to 601 total columns amounting to 50% structural similarity.


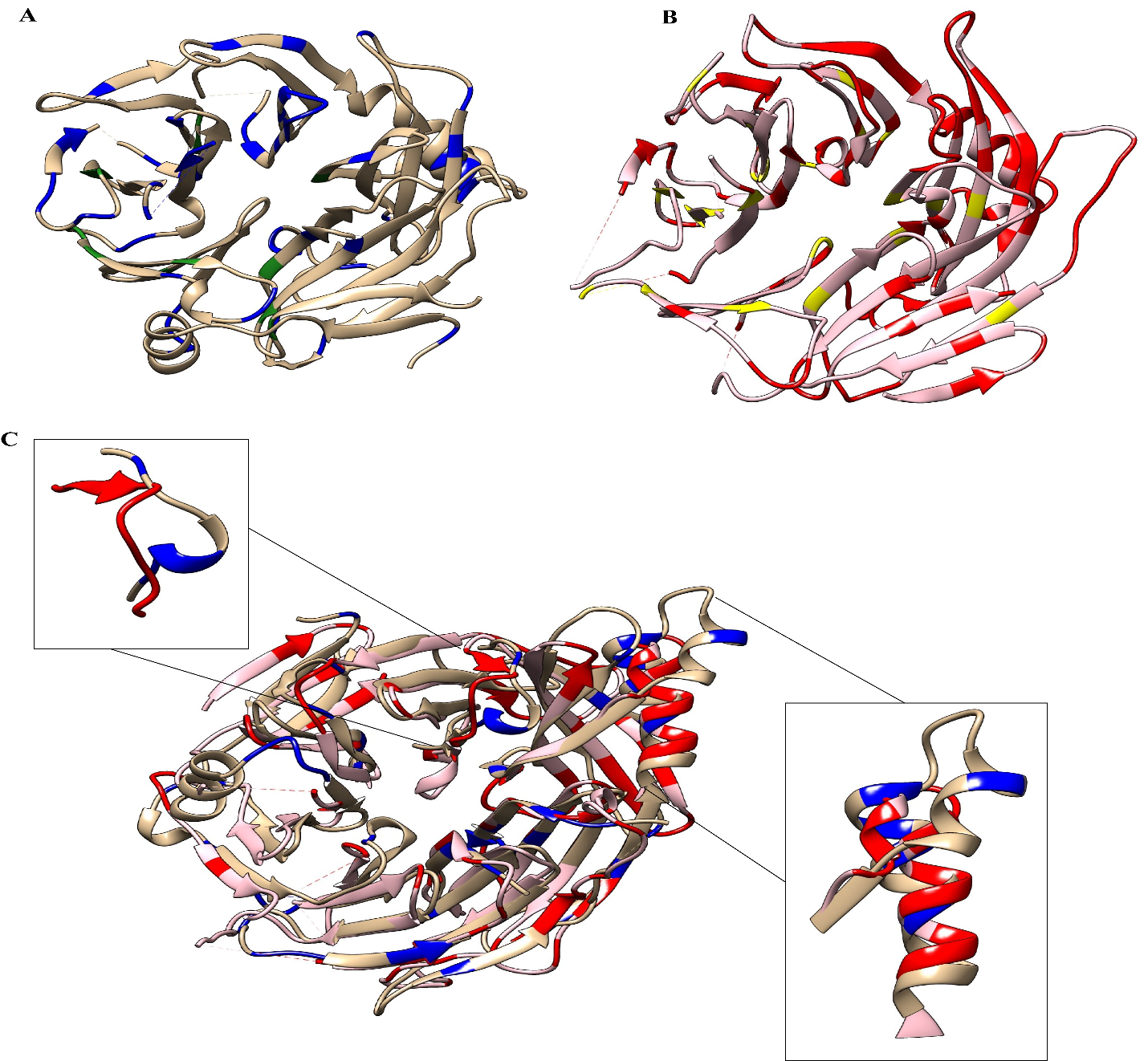


**Figure S5: Residues showing positive and purifying selection pressure in Nup133 N terminal domain**

A. *H. sapiens* Nup133 β propeller structure (PDB ID: 1XKS). The residues showing purifying selection pressure are marked in green and those showing positive selection pressure are marked in blue. B. *V. polyspora* Nup133N-terminal domain (PDB ID: 4Q9T). The residues showing purifying selection pressure are marked in yellow and those showing positive selection pressure are marked in red. C. Superimposition of 1XKS and 4Q9T depicting residues under positive selection pressure (blue for 1XKS and red for 4Q9T). Various positions which do not show structural similarity and do not superimpose are observed to be under positive selection pressure in continuous stretches.

**
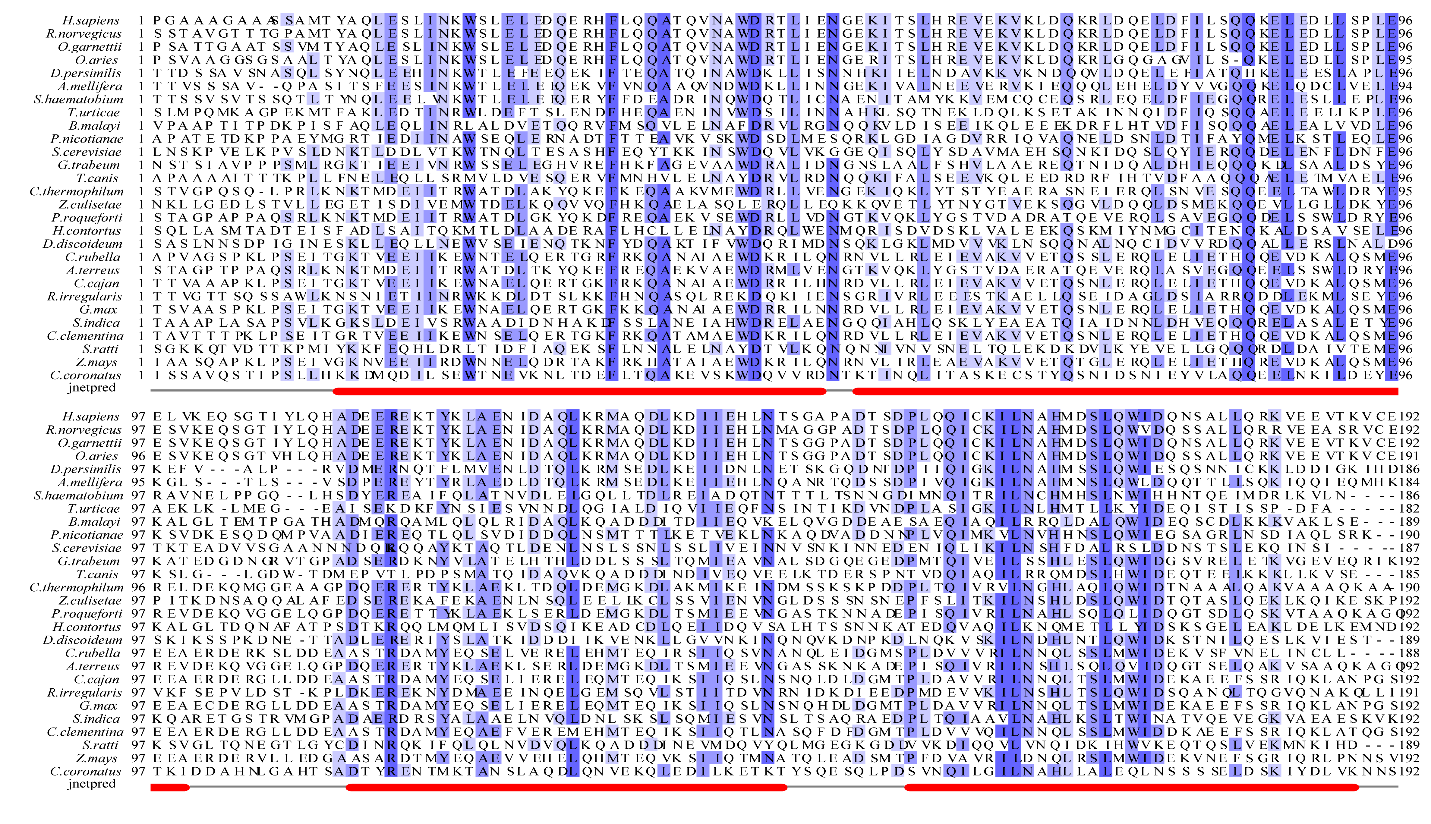
 Figure S6: Structure-guided alignment of Nup62 of the central channel**

A structure-guided alignment of 28 representative species from distant taxa. The alignment shown here contains only the α helical region of Nup62. The secondary structure prediction is shown below the alignment as obtained from JNET prediction server.

**
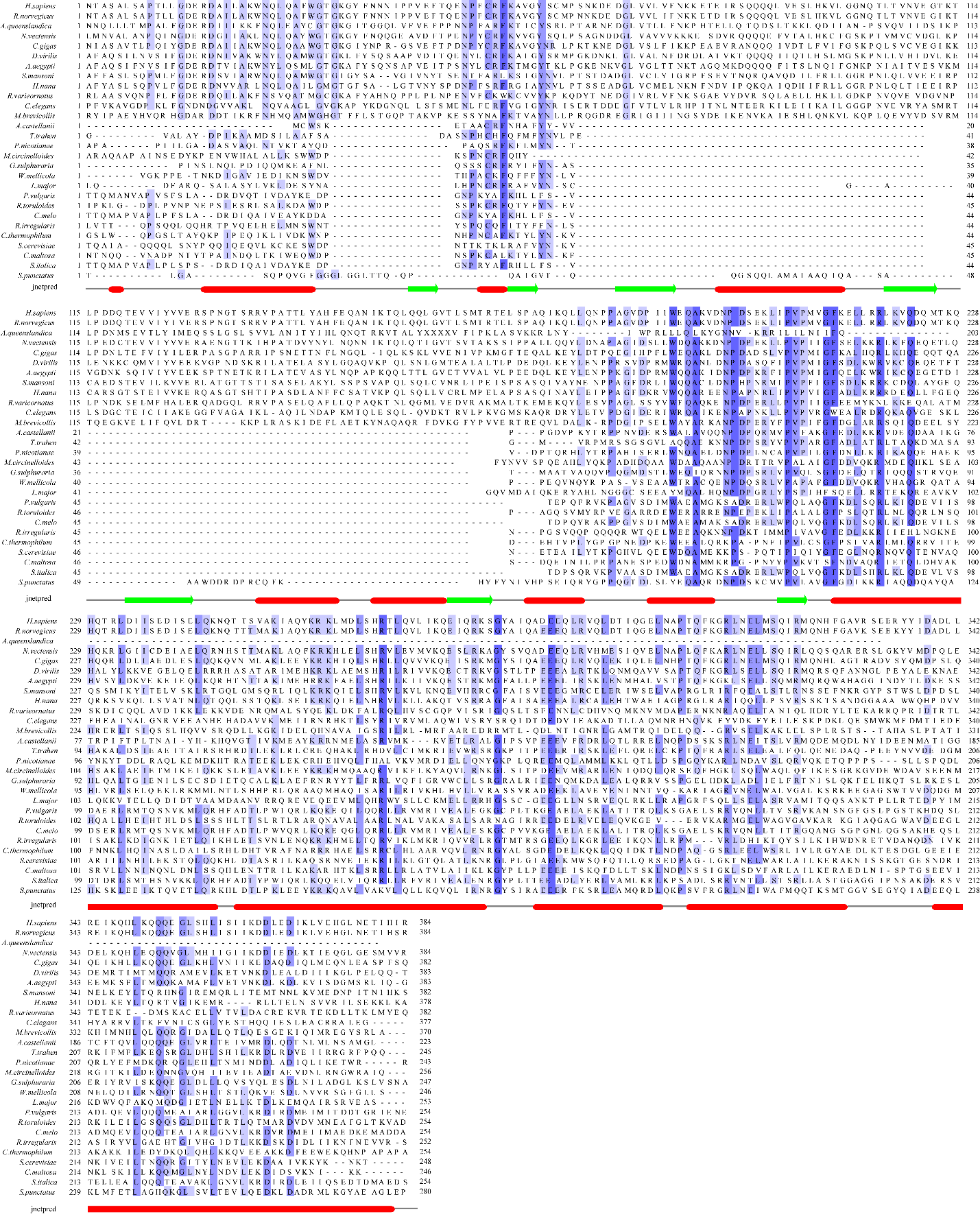
**

**Figure S7:** **A structure-guided alignment of Nup54 of the central channel**

A structure-guided alignment of 28 representative species from distant taxa. The alignment shown here contains only the structured regions of Nup54. The secondary structure prediction is shown below the alignment as obtained from JNET prediction server.


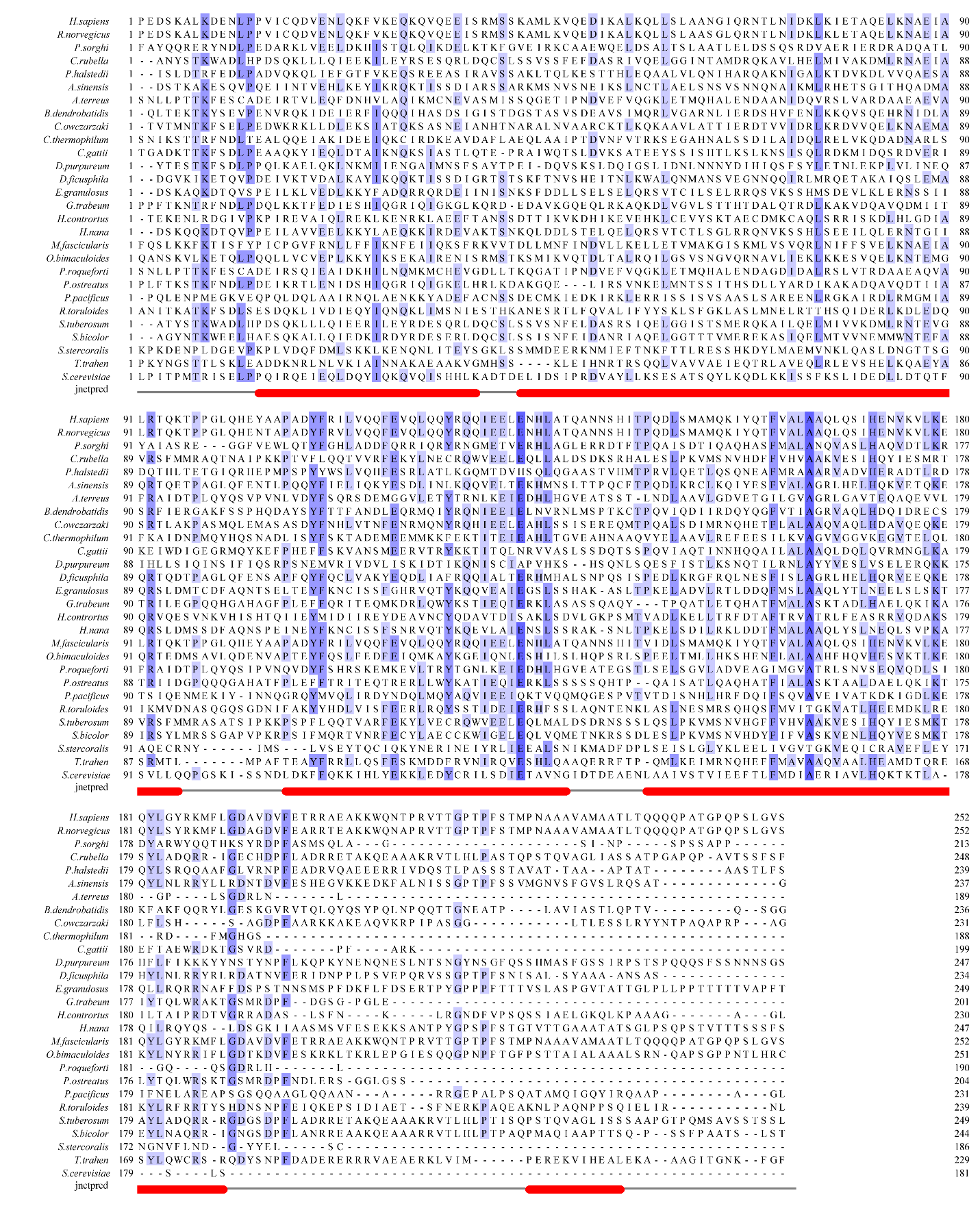


**Figure S8: A structure-guided alignment of Nup58 of the central channel**

A structure-guided alignment of 26 representative species from distant taxa. The alignment shown here contains only the α helical regions of Nup58. The secondary structure prediction is shown below the alignment as obtained from JNET prediction server.

**Evolutionary pressure analysis of central channel Nups**

The central channel proteins dataset was divided into two major classes viz. fungi and metazoans. In all six datasets were processed for evolutionary analysis, Nup62/ Nsp1, Nup54/ Nup57, Nup58/ Nup49. The amino acid sequences for each dataset were aligned separately using the PROMALS3D server. 25 sequences formed group 1 for Nsp1 (fungal homolog of Nup62) where *C. thermophilum* was taken as a representative sequence and 36 sequences formed group 2 for Nup62 where *H. sapiens* was taken as a representative sequence. BEB probability calculations were run on these two groups. For the structured region of Nsp1 (56), it was observed that 97% residues are under purifying section pressure and the remaining 3% under neutral selection pressure (Fig 5A). Similarly, for the structured region of Nup62 (323-522), it was observed that 94% residues are under purifying selection pressure and the remaining 6% under neutral selection pressure (Fig 5B) thus implying conservation of Nsp1/Nup62 across species.

25 sequences formed group 1 for Nup57 (fungal homolog of Nup54) with *C. thermophilum* as representative and 52 sequences formed group 2 for Nup54 with *H. sapiens* as representative. The length of both these homologous proteins is different due to the presence of an extended α/β region in the metazoan group (of about 143 amino acids) as compared to a smaller α/β region (of about 66 amino acids) in fungi. The BEB probability for the structured region of Nup57 (74-325) showed that 99% residues are under purifying selection pressure and only 1% under neutral selection pressure (Fig 5C). For the structured region of Nup54(190-507), 94% residues are under purifying selection pressure and remaining 6% under neutral selection pressure (Fig 5D) thus implying that the α helical domain is conserved across species and the additional α/β region of Nup57 is also conserved in the corresponding orthologs.

27 sequences formed group 1 for Nup49 (fungal homolog of Nup58) with *C. thermophilum* as representative and 68 sequences formed group 2 for Nup58 with *H. sapiens* as representative. For the structured region of Nup49 (245-470), only 56% residues are under purifying selection pressure and the remaining 44% under neutral selection pressure (Fig 5E). Contrary to this, the structured region of Nup58 (249-475), 88% residues are under purifying selection pressure and remaining 12% under neutral selection pressure (Fig 5F). Additionally, if we analyze the FG domain present only in the metazoan Nup58 at the C terminal, 84% of the low complexity regions, which are usually not conserved for other CTC proteins, are also under purifying selection pressure indicating divergent evolution of Nup58 of metazoans from the ancestral fungal Nup49.

**Phylogenetic tree of Nup58**

Figure S7 shows the phylogenetic spread of the Nup58 and its homologs across different phyla. The domains present in all these representative species considered in the phylogenetic tree were analyzed using HMMSCAN which led to the identification of three different domain organizations. Nucleoporin_FG2 domain (described as a family of chordate nucleoporins (PF15967)) was identified as the major domain in Nup58, which included both structured and unstructured regions of this protein. Whereas for Nup49 of yeast species (*S. cerevisiae* and *C. thermophilum*) only FG-repeat region was determined to have a Nucleoporin_FG domain (represents the family of Nups having FG repeat regions (PF13634)). There were also a few species which were predicted to contain both the domains. This observation is also concurrent with our selection pressure analysis. It is evident that a high percentage of residues of Nup58 and its homologous sequences are under neutral selection pressure and thus account for variability of amino acids in the homologous sequences of Nup58 might lead to the prediction of different PFAM domains.

**
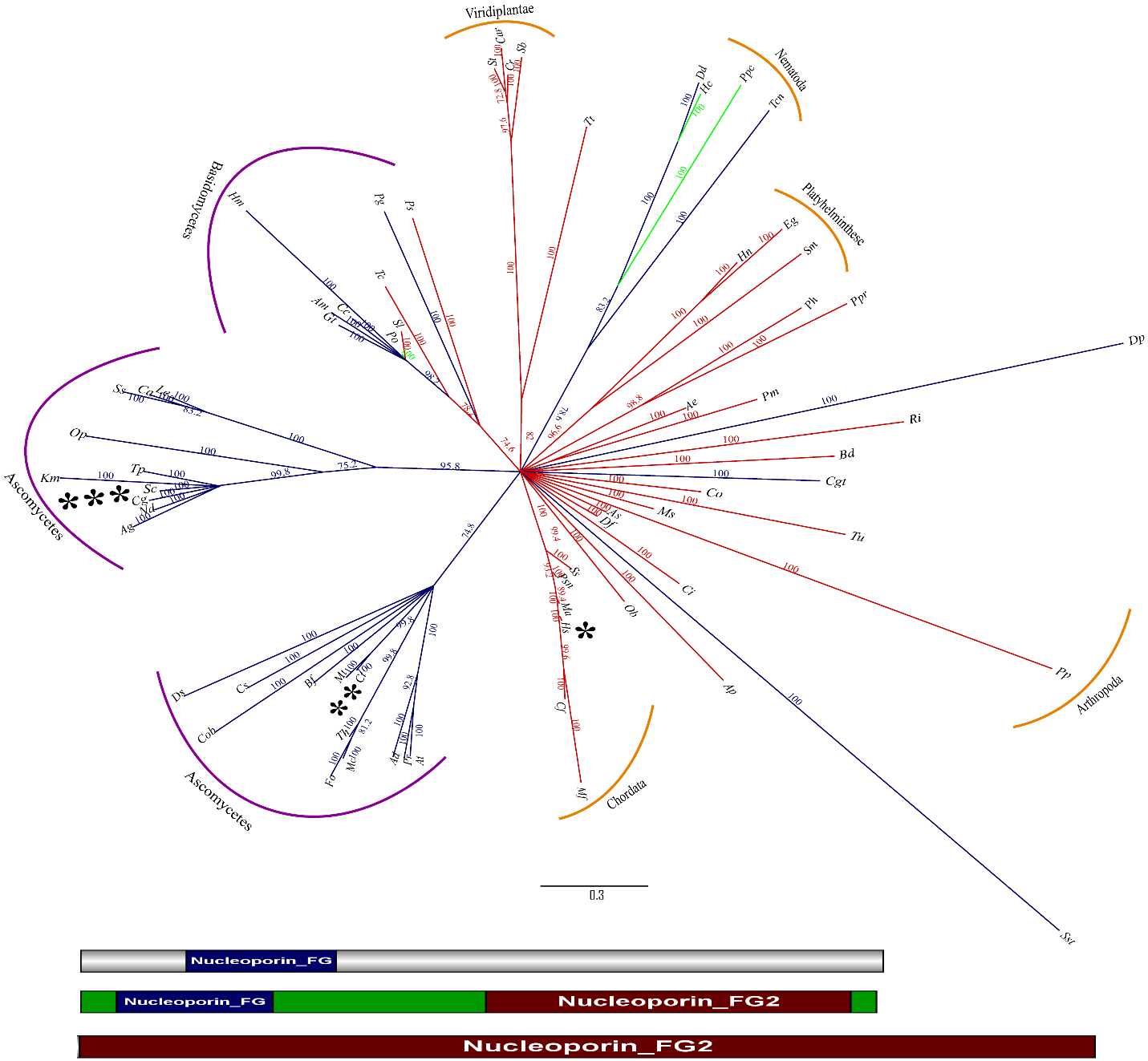
 Figure S9: Phylogenetic analysis and domain organization of Nup58 homologs**

Based on structure-guided multiple sequence alignment, representative homologs of Nup58 were subjected to phylogenetic analysis(neighbor-joining). In the unrooted tree, branch labels represent the percentage bootstrap values and the branch length are scaled to evolutionary distance. The branches are colored according to the type of domain present in different species, blue represents species predicted to have only Nucleoporin_FG domain, green predicted to have both Nucleoporin_FG2 and Nucleoporin_FG domain and red depicts that only Nucleoporin_FG2 was predicted for these group of species. The domain organization of the three classes of Nup58 is shown below the tree. The species names are abbreviated for the ease of representation and the detailed information is provided separately in the file S10 (* *H. sapiens*, ** *C. thermophilum,* and *** *S. cerevisiae*). The fungal species are grouped under purple color and the metazoan species under the orange color bars.

**Table S7: Details of templated used for threading the sequences of hNup58 and its fungal homologs (scNup49 and ctNup49).**

| \| **Protein** \| ***Species*** \| **Query Length** \| **Query Coverage** \| **Template PDB ID** \| **Template Description** \| **p-value** \| \| --- \| --- \| --- \| --- \| --- \| --- \| --- \| \| Nup58 \| *H. sapiens* \| 233 \| 100% \| 3R6N \| Desmoplakin from *H. sapiens* \| 4.00E-04 \| \| Nup49 \| *S. cerevisiae* \| 206 \| 100% \| 1HCI \| Alpha actinin 2 from *H. sapiens* \| 0.002 \| \| Nup49 \| *C. thermophilum* \| 230 \| 92% \| 1U5P \| Chicken brain alpha spectrin \| 9.00E-04 \| |  |  |  |  |  |  |
| --- | --- | --- | --- | --- | --- | --- | --- | --- | --- | --- | --- | --- | --- | --- | --- | --- | --- | --- | --- | --- | --- | --- | --- | --- | --- | --- | --- | --- | --- | --- | --- | --- | --- | --- |
|  |  |  |  |  |  |  |
|  |  |  |  |  |  |  |

**
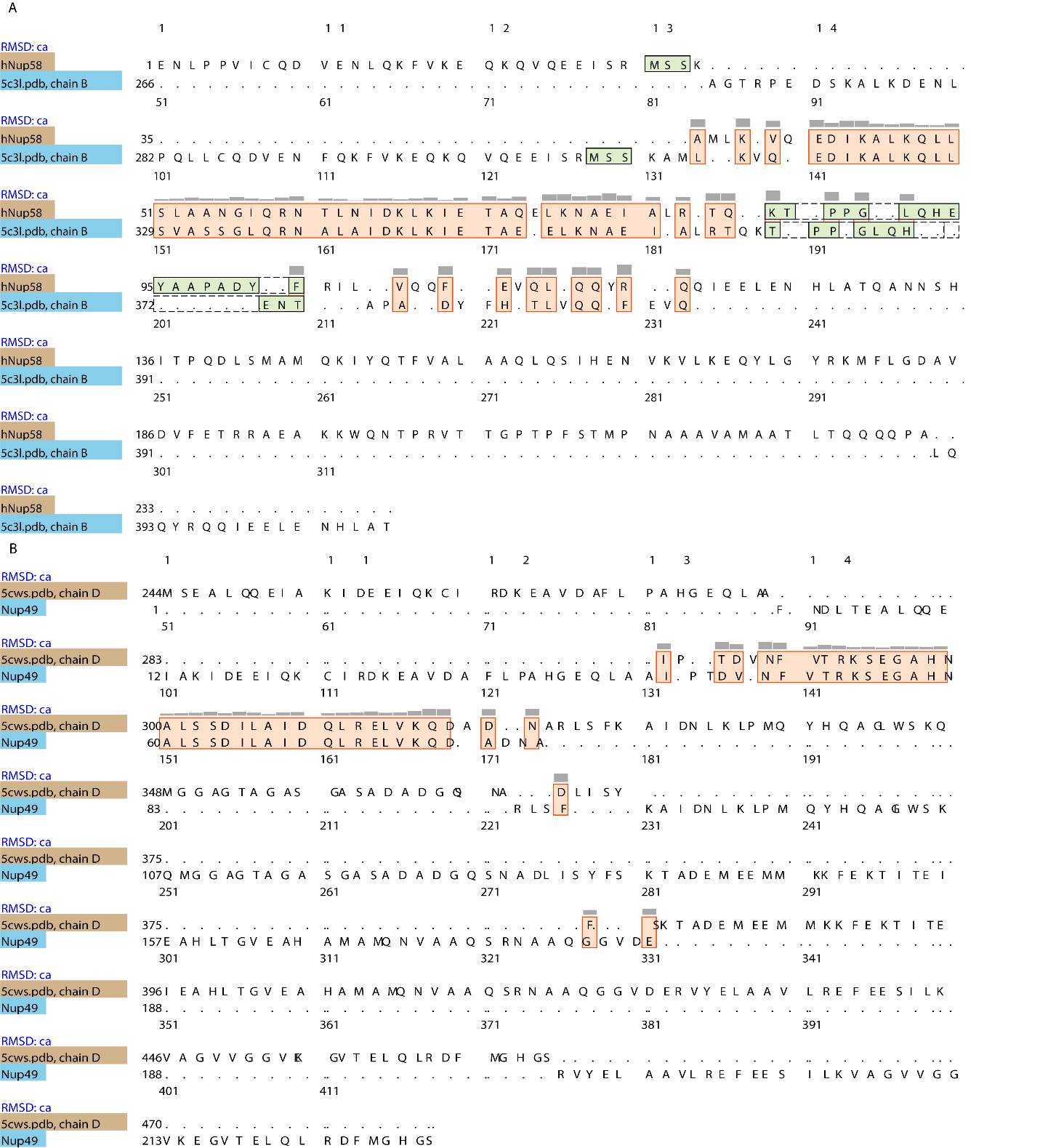
Figure S10: Multiple sequence alignment after structure superimposition**

A. Nup58 modeled structure was superimposed on the Nup58 chain of PDB ID: 5C3L. The residues that superimpose are shown in the red box and those present in loop 1 and 2 responsible for the conformation changes in the helices are highlighted in the green box. B. Modelled Nup49 structure of *C. thermophilum* was superimposed on the Nup49 chain of PDB ID 5CWS. The residues that superimpose are shown in the red box

**
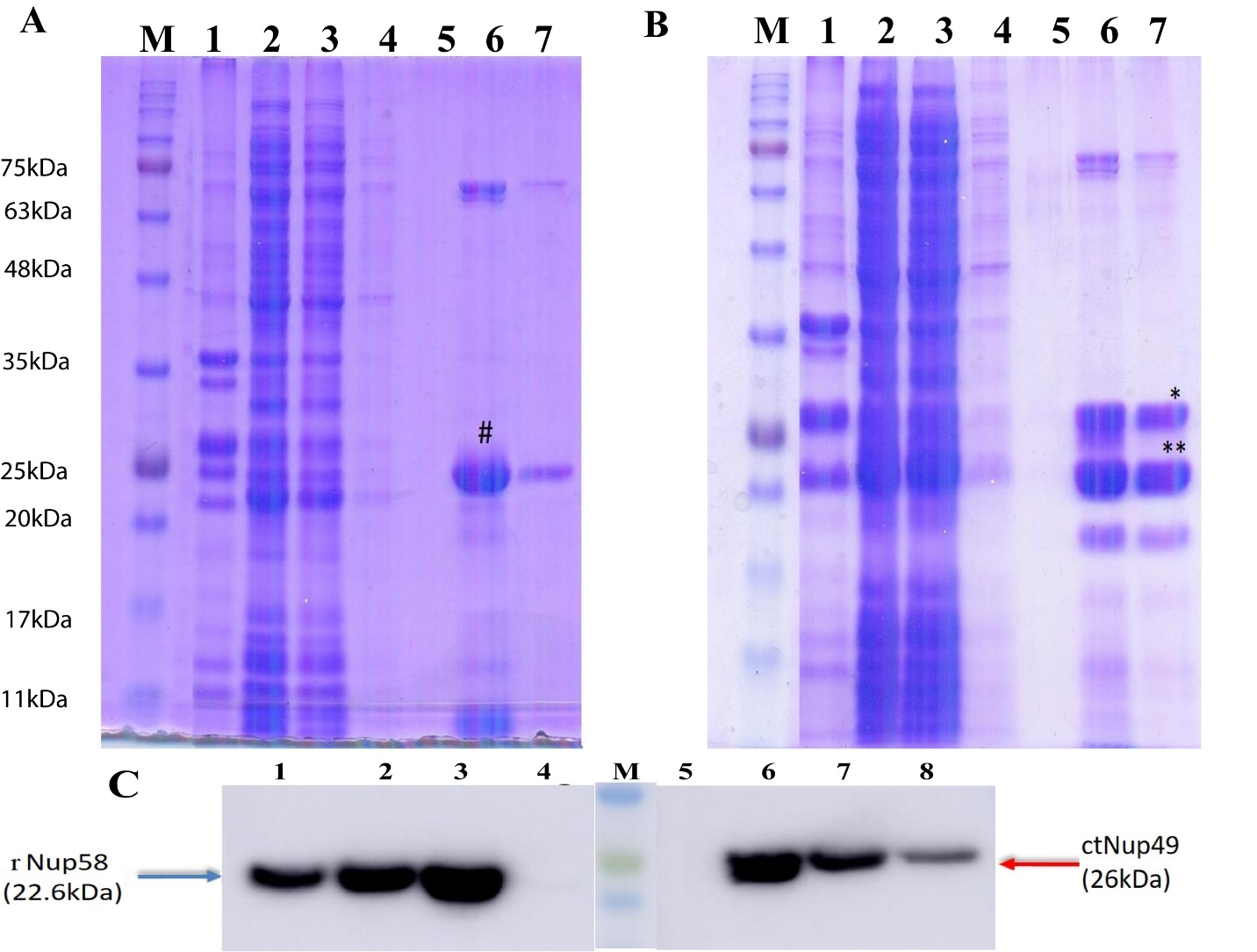
**

**Figure S11: Ni-NTA pull-down assay of Rat ternary complex compared with Chaetomium chimeric construct**

Ni-NTA affinity purification using His_6_-tag on Nup58 and Nup49 was performed to pull out the interacting partners in the central channel (Nup54 and Nup62) **A)** 12% SDS-PAGE scan after Ni-NTA purification of Chaetomium chimeric construct # ctNup49 (M: Ladder prestained Abcam (116028), Lane 1: Pellet, Lane 2: Supernatant, Lane 3: Flow-through, Lane 4: First wash, Lane 5: Last wash, Lane 6-7: Elutions ) **B)** 12% SDS-PAGE scan after Ni-NTA purification of Rat trimeric construct * rNup62, ** rNup58 and rNup54 run as a doublet band since they have nearly same molecular weights i.e. rNup58 is 22.6kDa with His_6_ tag and rNup54 is 21kDa **C**) Western blot analysis using anti-His antibody to validate expression of both ctNup49 and rNup58 with Anti-His antibody (Sigma) in 1:3000 ratio. Lane 1-3: Elution fractions (rat), Lane 4: Flow-through (rat) M: Ladder (pre-stained Abcam (116028)), Lane 5: Flow-through (ct) Lane 6-8: Elution fractions (ct) (ct: *C. thermophilum* r: *R. norvegicus*)

**Table S8: Details of abbreviation used for species name in Fig 3(ELYS phylogenetic tree)**

| **Species name** | **Abbreviation** | **Species name** | **Abbreviation** |
| --- | --- | --- | --- |
| *Aedes aegypti* | Aag | *Drosophila pseudoobscura* | Dp |
| *Agrilus planipennis* | Ap | *Drosophilla melanogaster* | Dml |
| *Amazona aestiva* | Aas | *Drosophilla willistoni* | Dw |
| *Anopheles merus* | Am | *Echinococcus multilocularis* | Em |
| *Anopheles sinensis* | As | *Exidia glandulosa* | Eg |
| *Arabis alphina* | Aal | *Exophiala dermatitidis* | Ed |
| *Arthroderma otae* | Ao | *Fusarium oxysporum* | Fo |
| *Aspergillus carbonarius* | Ac | *Gallus gallus* | Gg |
| *Aspergillus oryzae* | Aor | *Glossina morsitans* | Gm |
| *Atta cephalotes* | Atc | *Grosmannia clavigera* | Gc |
| *Beauveria bassiana* | Bb | *Homo sapiens* | Hs |
| *Blumeria graminis* | Bg | *Hypocrea virens* | Hv |
| *Bombyx mori* | Bmr | *Lentinula edodes* | Le |
| *Brugia malayi* | Bml | *Lepisosteus oculatus* | Lo |
| *Cajanus cajan* | Cc | *Lingula unguis* | Lu |
| *Canis lupus* | Cl | *Magnaporthe oryzae* | Mo |
| *Chaetomium thermophilum* | Ct | *Magnaporthiopsis poae* | Mp |
| *Chlorocebus sabaeus* | Cs | *Marchantia polymorpha* | Mpl |
| *Colletotrichum gramincola* | Cgr | *Marssonina brunnea* | Mb |
| *Colletotrichum gloeosporiodes* | Cgl | *Ricinus communis* | Rc |
| *Conidiobolus coronatus* | Ccr | *Poecilia formosa* | Pf |
| *Cricetulus griseus* | Cgs | *Pseudozyma antarctica* | Pa |
| *Cucumis melo* | Cm | *Pyrenophora tritici-repentis* | Pt |
| *Drosophila ficusphila* | Df | *Rosellina necatrix* | Rn |
| *Meloidogyne hapla* | Mh | *Stomoxys calcitrans* | Sc |
| *Mesocestoides corti* | Mc | *Strongyloides papillosus* | Spp |
| *Metarhizium anisopliae* | Ma | *Tetraodon nigroviridis* | Tn |
| *Nicotiana attenuata* | Na | *Thecamonas trahen* | Tt |
| *Onchocerca volvulus* | Ov | *Theobroma cacao* | Tc |
| *Opisthorchis viverrini* | Ovi | *Thielavia terrestris* | Ttr |
| *Oryctolagus cuniculus* | Oc | *Toxocara canis* | Tcn |
| *Oryza punctata* | Op | *Trametes pubescens* | Tp |
| *Phialocephala scopiformis* | Ps | *Tribolium castaneum* | Tcs |
| *Salpingoeca rosetta* | Sr | *Trichinella britovi* | Tb |
| *Schizosaccharomyces pombe* | Sp | *Trichinella spiralis* | Ts |
| *Setosphaeria turcica* | St | *Trichosporon asahii* | Ta |
| *Sorghum bicolor* | Sb | *Triticum aestivum* | Tas |
| *Stegodyphus mimosarum* | Sm |  |  |

**Table S9: Details of abbreviation used for species name in Fig 4(Nup133 phylogenetic tree)**

| **Species name** | **Abbreviation** | **Species name** | **Abbreviation** |
| --- | --- | --- | --- |
| *Absidia glauca* | Ag | *Glossina austeni* | Ga |
| *Agaricus bisporus* | Ab | *Glycine soja* | Gs |
| *Anopheles funestus* | Af | *Haemonchus contrortus* | Hc |
| *Apis mellifera* | Am | *Hebeloma cylindrosporum* | Hcy |
| *Arabidopsis thaliana* | At | *Homo sapiens* | Hs |
| *Arabis alphina* | Aa | *Hypocrea virens* | Hv |
| *Arthrobotrys oligospora* | Aol | *Ixodes scapularis* | Is |
| *Arthroderma otae* | Aot | *Jatropha curcas* | Jc |
| *Ascaris suum* | As | *Kluyveromyces marxianus* | Km |
| *Aspergillus nomius* | An | *Lachancea themotolerans* | Lt |
| *Aspergillus tubingensis* | Atb | *Lentinula edodes* | Le |
| *Batrachochytrium dendrobatidis* | Bd | *Magnaporthe oryzae* | Mo |
| *Brugia malayi* | Bm | *Malassezia sympodialis* | Ms |
| *Caenorhabditis tropicalis* | Ctr | *Marchantia polymorpha* | Mp |
| *Caenorhabditis elegans* | Ce | *Metacordyceps chlamydosporia* | Mc |
| *Calypte anna* | Ca | *Mixia osmundae* | Mos |
| *Candida albicans* | Cal | *Monodelphis domestica* | Md |
| *Candida maltosa* | Cm | *Myceliophthora thermophila* | Mt |
| *Ceratocystis fimbriata* | Cf | *Myotis davidii* | Mdv |
| *Chaetomium thermophilum* | Ct | *Myotis lucifugus* | Ml |
| *Clonorchis sinensis* | Cs | *Nicotiana sylvestris* | Ns |
| *Colletotrichum higginsianum* | Ch | *Ornithorhynchus anatinus* | Oa |
| *Crassostrea gigas* | Cg | *Ostreococcus lucimarinus* | Ol |
| *Cucumis melo* | Cml | *Penicillium expansum* | Pe |
| *Daphnia pulex* | Dp | *Phaseolu1s vulgaris* | Pv |
| *Dictyostelium discoideum* | Dd | *Phytophthora nicotianae* | Pn |
| *Dictyostelium fasciculatum* | Dfs | *Plasmopara halstedii* | Ph |
| *Drosophila ficusphila* | Df | *Pyrenophora teres* | Pt |
| *Drosophila persimilis* | Dp | *Rhizoctonia solani* | Rs |
| *Echinococcus multilocularis* | Em | *Rhodosporidium toruloides* | Rt |
| *Egretta garzetta* | Eg | *Saccharomyces arboricola* | Sa |
| *Enterobius vermicularis* | Ev | *Saccharomyces cerevisiae* | Sc |
| *Equus caballus* | Ec | *Schistosoma haematobium* | Sh |
| *Eremothecium cymbalariae* | Ec | *Sclerotinia sclerotiorum* | Ss |
| *Exidia glandulosa* | Egl | *Setaria italica* | Si |
| *Ficedula albicollis* | Fa | *Setosphaeria turcica* | St |
| *Sphaerulina musiva* | Sm | *Thelazia callipaeda* | Tcp |
| *Strongyloides venezuelensis* | Sv | *Tribolium castaneum* | Tct |
| *Strongyloides ratti* | Sr | *Wallemia mellicola* | Wm |
| *Talaromyces marneffei* | Tm | *Xiphophorus maculatus* | Xm |
| *Thanatephorus cucumeris* | Tc | *Zygosaccharomyces bailii* | Zb |

**Table S10: Details of abbreviation used for species name in Supplementary Figure 8 (Nup58 phylogenetic tree)**

| **Species name** | **Abbreviation** | **Species name** | **Abbreviation** |
| --- | --- | --- | --- |
| *Acromyrmex echinatior* | Ae | *Macaca fascicularis* | Mf |
| *Acyrthosiphon pisum* | Ap | *Megaselia scalaris* | Ms |
| *Ajellomyces dermatitidis* | Ad | *Mesocricetus auratus* | Ma |
| *Amanita muscaria* | Am | *Metacordyceps chlamydosporia* | Mc |
| *Anopheles sinensis* | As | *Myceliophthora thermophila* | Mt |
| *Ashbya gossypii* | Ag | *Naumovozyma dairenensis* | Nd |
| *Aspergillus terreus* | At | *Octopus bimaculoides* | Ob |
| *Batrachochytrium dendrobatidis* | Bd | *Ogataea parapolymorpha* | Op |
| *Botryotinia fuckeliana* | Bf | *Papilio machaon* | Pm |
| *Camelus ferus* | Cf | *Pelodiscus sinensis* | Psn |
| *Candida albicans* | Ca | *Penicillium roqueforti* | Pr |
| *Candida glabrata* | Cg | *Phlebotomus papatasi* | Pp |
| *Capsaspora owczarzaki* | Co | *Phytophthora parasitica* | Ppr |
| *Capsella rubella* | Cr | *Plasmopara halstedii* | Ph |
| *Chaetomium thermophilum* | Ct | *Pleurotus ostreatus* | Po |
| *Cicer arietinum* | Car | *Pristionchus pacificus* | Ppc |
| *Ciona intestinalis* | Ci | *Puccinia sorghi* | Ps |
| *Cochliobolus sativus* | Cs | *Puccinia graminis* | Pg |
| *Colletotrichum orbiculare* | Cob | *Rhizophagus irregularis* | Ri |
| *Coprinopsis cinerea* | Cc | *Saccharomyces cerevisiae* | Sc |
| *Cryptococcus gattii* | Cgt | *Salmo salar* | Ss |
| *Dictyostelium purpureum* | Dp | *Scheffersomyces stipitis* | Ss |
| *Dictyostelium discoideum* | Dd | *Schistosoma mansoni* | Sm |
| *Dothistroma septosporum* | Ds | *Serpula lacrymans* | Sl |
| *Drosophila ficusphila* | Df | *Solanum tuberosum* | St |
| *Echinococcus granulosus* | Eg | *Sorghum bicolor* | Sb |
| *Fusarium oxysporum* | Fo | *Strongyloides stercoralis* | Sst |
| *Gloeophyllum trabeum* | Gt | *Tetranychus urticae* | Tu |
| *Haemonchus contrortus* | Hc | *Tetrapisispora phaffii* | Tp |
| *Homo sapiens* | Hs | *Thanatephorus cucumeris* | Tc |
| *Hymenolepis nana* | Hn | *Thecamonas trahen* | Tt |
| *Hypsizygus marmoreus* | Hm | *Toxocara canis* | Tcn |
| *Kluyveromyces marxianus* | Km | *Trichoderma harzianum* | Th |
| *Lodderomyces elongisporus* | Le |  |  |

**Table S11: Details of species used to generate supertree (Fig 8)**

| **Uniprot**  **Mnemonic** | **Species Name** | **Classification** |
| --- | --- | --- |
| BABBI | Babesia bigemina | Alveolata |
| BABBO | Babesia bovis | Alveolata |
| BABMR | Babesia microti | Alveolata |
| CRYMR | Cryptosporidium muris | Alveolata |
| CRYPI | Cryptosporidium parvum | Alveolata |
| 9EIME | Eimeria necatrix | Alveolata |
| HAMHA | Hammondia hammondi | Alveolata |
| ICHMG | Ichthyophthirius multifiliis | Alveolata |
| NEOCL | Neospora caninum | Alveolata |
| 9SPIT | Oxytricha trifallax | Alveolata |
| PARTE | Paramecium tetraurelia | Alveolata |
| PERM5 | Perkinsus marinus | Alveolata |
| PLAF7 | Plasmodium falciparum | Alveolata |
| PLAVN | Plasmodium vinckei | Alveolata |
| PSEPJ | Pseudocohnilembus persalinus | Alveolata |
| 9CILI | Stentor coeruleus | Alveolata |
| STYLE | Stylonychia lemnae | Alveolata |
| SYMMI | Symbiodinium microadriaticum | Alveolata |
| TETTS | Tetrahymena thermophila | Alveolata |
| THEEQ | Theileria equi | Alveolata |
| THEPA | Theileria parva | Alveolata |
| TOXGV | Toxoplasma gondii | Alveolata |
| EIMAC | Eimeria acervulina | Apicomplexan |
| ACACA | Acanthamoeba castellanii | Amoebozoa |
| DICDI | Dictyostelium discoideum | Amoebozoa |
| DICFS | Dictyostelium fasciculatum | Amoebozoa |
| 9MYCE | Dictyostelium lacteum | Amoebozoa |
| DICPU | Dictyostelium purpureum | Amoebozoa |
| ENTHI | Entamoeba histolytica | Amoebozoa |
| ENTIV | Entamoeba invadens | Amoebozoa |
| POLPP | Polysphondylium pallidum | Amoebozoa |
| HELRO | Helobdella robusta | Annelida |
| THETB | Thecamonas trahens | Apusozoa |
| ACREC | Acromyrmex echinatior | Arthropoda |
| ACYPI | Acyrthosiphon pisum | Arthropoda |
| AEDAE | Aedes aegypti | Arthropoda |
| AEDAL | Aedes albopictus | Arthropoda |
| AGRPL | Agrilus planipennis | Arthropoda |
| 9ACAR | Amblyomma triste | Arthropoda |
| 9MUSC | Anastrepha obliqua | Arthropoda |
| ANOAL | Anopheles albimanus | Arthropoda |
| ANOAR | Anopheles arabiensis | Arthropoda |
| ANODA | Anopheles darlingi | Arthropoda |
| ANOFN | Anopheles funestus | Arthropoda |
| ANOGA | Anopheles gambiae | Arthropoda |
| ANOME | Anopheles merus | Arthropoda |
| 9DIPT | Anopheles minimus | Arthropoda |
| ANOQN | Anopheles quadriannulatus | Arthropoda |
| ANOSI | Anopheles sinensis | Arthropoda |
| ANOST | Anopheles stephensi | Arthropoda |
| APIME | Apis mellifera | Arthropoda |
| ATTCE | Atta cephalotes | Arthropoda |
| 9HYME | Atta colombica | Arthropoda |
| BOMMO | Bombyx mori | Arthropoda |
| CAMFO | Camponotus floridanus | Arthropoda |
| 9NEOP | Chilo auricilius | Arthropoda |
| CULQU | Culex quinquefasciatus | Arthropoda |
| DANPL | Danaus plexippus | Arthropoda |
| 9CRUS | Daphnia magna | Arthropoda |
| DAPPU | Daphnia pulex | Arthropoda |
| DENPD | Dendroctonus ponderosae | Arthropoda |
| DIACI | Diaphorina citri | Arthropoda |
| DROAN | Drosophila ananassae | Arthropoda |
| DROBS | Drosophila busckii | Arthropoda |
| DROFC | Drosophila ficusphila | Arthropoda |
| DROGR | Drosophila grimshawi | Arthropoda |
| DROME | Drosophila melanogaster | Arthropoda |
| DROMO | Drosophila mojavensis | Arthropoda |
| DROPE | Drosophila persimilis | Arthropoda |
| DROPS | Drosophila pseudoobscura | Arthropoda |
| DROSE | Drosophila sechellia | Arthropoda |
| DROSI | Drosophila simulans | Arthropoda |
| DROVI | Drosophila virilis | Arthropoda |
| DROWI | Drosophila willistoni | Arthropoda |
| GLOAU | Glossina austeni | Arthropoda |
| GLOFF | Glossina fuscipes | Arthropoda |
| GLOMM | Glossina morsitans | Arthropoda |
| GLOPL | Glossina pallidipes | Arthropoda |
| HARSA | Harpegnathos saltator | Arthropoda |
| IXOSC | Ixodes scapularis | Arthropoda |
| LASNI | Lasius niger | Arthropoda |
| LUCCU | Lucilia cuprina | Arthropoda |
| LUTLO | Lutzomyia longipalpis | Arthropoda |
| MEGSC | Megaselia scalaris | Arthropoda |
| MUSDO | Musca domestica | Arthropoda |
| NASVI | Nasonia vitripennis | Arthropoda |
| CERBI | Ooceraea biroi | Arthropoda |
| ORCCI | Orchesella cincta | Arthropoda |
| 9SCAR | Oryctes borbonicus | Arthropoda |
| PAPMA | Papilio machaon | Arthropoda |
| PAPXU | Papilio xuthus | Arthropoda |
| PEDHC | Pediculus humanus subsp. corporis | Arthropoda |
| PHLPP | Phlebotomus papatasi | Arthropoda |
| 9MICR | Pseudacanthops centralis | Arthropoda |
| RHOPR | Rhodnius prolixus | Arthropoda |
| SARSC | Sarcoptes scabiei | Arthropoda |
| SOLIN | Solenopsis invicta | Arthropoda |
| 9ARAC | Stegodyphus mimosarum | Arthropoda |
| STOCA | Stomoxys calcitrans | Arthropoda |
| STRMM | Strigamia maritima | Arthropoda |
| TETUR | Tetranychus urticae | Arthropoda |
| TRICA | Tribolium castaneum | Arthropoda |
| ZOONE | Zootermopsis nevadensis | Arthropoda |
| ACRC1 | Acremonium chrysogenum | Ascomycetes |
| AJECN | Ajellomyces capsulatus | Ascomycetes |
| AJEDR | Ajellomyces dermatitidis | Ascomycetes |
| ALTAL | Alternaria alternata | Ascomycetes |
| ARTOA | Arthrobotrys oligospora | Ascomycetes |
| ARTBC | Arthroderma benhamiae | Ascomycetes |
| ARTGP | Arthroderma gypseum | Ascomycetes |
| ARTOC | Arthroderma otae | Ascomycetes |
| ASHGO | Ashbya gossypii | Ascomycetes |
| ASPAC | Aspergillus aculeatus | Ascomycetes |
| ASPC5 | Aspergillus carbonarius | Ascomycetes |
| ASPCL | Aspergillus clavatus | Ascomycetes |
| ASPFN | Aspergillus flavus | Ascomycetes |
| ASPGL | Aspergillus glaucus | Ascomycetes |
| ASPKW | Aspergillus kawachii | Ascomycetes |
| EMENI | Aspergillus nidulans | Ascomycetes |
| ASPNG | Aspergillus niger | Ascomycetes |
| ASPNO | Aspergillus nomius | Ascomycetes |
| ASPOR | Aspergillus oryzae | Ascomycetes |
| ASPPU | Aspergillus parasiticus | Ascomycetes |
| ASPTN | Aspergillus terreus | Ascomycetes |
| ASPTU | Aspergillus tubingensis | Ascomycetes |
| ASPVE | Aspergillus versicolor | Ascomycetes |
| ASPWE | Aspergillus wentii | Ascomycetes |
| AURPU | Aureobasidium pullulans | Ascomycetes |
| BAUCO | Baudoinia compniacensis | Ascomycetes |
| BEABA | Beauveria bassiana | Ascomycetes |
| BLAGS | Blastomyces gilchristii | Ascomycetes |
| BLUGR | Blumeria graminis | Ascomycetes |
| BOTF1 | Botryotinia fuckeliana | Ascomycetes |
| BYSSN | Byssochlamys spectabilis | Ascomycetes |
| CANAL | Candida albicans | Ascomycetes |
| CANGA | Candida glabrata | Ascomycetes |
| CANMX | Candida maltosa | Ascomycetes |
| CANPC | Candida parapsilosis | Ascomycetes |
| CANTC | Candida tenuis | Ascomycetes |
| CANTT | Candida tropicalis | Ascomycetes |
| CERFI | Ceratocystis fimbriata | Ascomycetes |
| CHAGB | Chaetomium globosum | Ascomycetes |
| CHATD | Chaetomium thermophilum | Ascomycetes |
| CLAP2 | Claviceps purpurea | Ascomycetes |
| CLAL4 | Clavispora lusitania | Ascomycetes |
| COCIT | Coccidioides immitis | Ascomycetes |
| COCPS | Coccidioides posadasii | Ascomycetes |
| COCCA | Cochliobolus carbonum | Ascomycetes |
| COCH5 | Cochliobolus heterostrophus | Ascomycetes |
| COCSN | Cochliobolus sativus | Ascomycetes |
| COLGN | Colletotrichum gloeosporioides | Ascomycetes |
| COLGM | Colletotrichum graminicola | Ascomycetes |
| COLHI | Colletotrichum higginsianum | Ascomycetes |
| COLOR | Colletotrichum orbiculare | Ascomycetes |
| COLSU | Colletotrichum sublineola | Ascomycetes |
| CONA1 | Coniosporium apollinis | Ascomycetes |
| CORDF | Cordyceps confragosa | Ascomycetes |
| CORMM | Cordyceps militaris | Ascomycetes |
| CYBFA | Cyberlindnera fabianii | Ascomycetes |
| CYBJA | Cyberlindnera jadinii | Ascomycetes |
| DACHA | Dactylellina haptotyla | Ascomycetes |
| DEBHA | Debaryomyces hansenii | Ascomycetes |
| DEKBR | Dekkera bruxellensis | Ascomycetes |
| DIDRA | Didymella rabiei | Ascomycetes |
| DOTSN | Dothistroma septosporum | Ascomycetes |
| ENDPU | Endocarpon pusillum | Ascomycetes |
| ERECY | Eremothecium cymbalariae | Ascomycetes |
| EUTLA | Eutypa lata | Ascomycetes |
| EXODN | Exophiala dermatitidis | Ascomycetes |
| 9EURO | Exophiala xenobiotica | Ascomycetes |
| FUSLA | Fusarium langsethiae | Ascomycetes |
| FUSPO | Fusarium poae | Ascomycetes |
| 9PEZI | Fusarium pseudograminearum | Ascomycetes |
| GAGT3 | Gaeumannomyces graminis var. tritici | Ascomycetes |
| GIBF5 | Gibberella fujikuroi | Ascomycetes |
| GIBM7 | Gibberella moniliformis | Ascomycetes |
| GIBZE | Gibberella zeae | Ascomycetes |
| GLAL7 | Glarea lozoyensis | Ascomycetes |
| GROCL | Grosmannia clavigera | Ascomycetes |
| HANUV | Hanseniaspora uvarum | Ascomycetes |
| HYPAI | Hypocrea atroviridis | Ascomycetes |
| HYPJQ | Hypocrea jecorina | Ascomycetes |
| HYPVG | Hypocrea virens | Ascomycetes |
| KAZAF | Kazachstania africana | Ascomycetes |
| KAZNA | Kazachstania naganishii | Ascomycetes |
| 9SACH | Kluyveromyces dobzhanskii | Ascomycetes |
| KLULA | Kluyveromyces lactis | Ascomycetes |
| KLUMA | Kluyveromyces marxianus | Ascomycetes |
| KOMPG | Komagataella phaffii | Ascomycetes |
| LACFM | Lachancea fermentati | Ascomycetes |
| LACTC | Lachancea thermotolerans | Ascomycetes |
| LEPMJ | Leptosphaeria maculans | Ascomycetes |
| LIPST | Lipomyces starkeyi | Ascomycetes |
| LODEL | Lodderomyces elongisporus | Ascomycetes |
| MACPH | Macrophomina phaseolina | Ascomycetes |
| MAGO7 | Magnaporthe oryzae | Ascomycetes |
| MAGP6 | Magnaporthiopsis poae | Ascomycetes |
| MARBU | Marssonina brunnea | Ascomycetes |
| METCM | Metacordyceps chlamydosporia | Ascomycetes |
| METAQ | Metarhizium acridum | Ascomycetes |
| METAN | Metarhizium anisopliae | Ascomycetes |
| METRA | Metarhizium robertsii | Ascomycetes |
| PICGU | Meyerozyma guilliermondii | Ascomycetes |
| MYCTT | Myceliophthora thermophila | Ascomycetes |
| NAUCC | Naumovozyma castellii | Ascomycetes |
| NAUDC | Naumovozyma dairenensis | Ascomycetes |
| NECH7 | Nectria haematococca | Ascomycetes |
| NEOFI | Neosartorya fischeri | Ascomycetes |
| ASPFU | Neosartorya fumigata | Ascomycetes |
| NEUCR | Neurospora crassa | Ascomycetes |
| NEUT9 | Neurospora tetrasperma | Ascomycetes |
| OGAPD | Ogataea parapolymorpha | Ascomycetes |
| OPHSC | Ophiocordyceps sinensis | Ascomycetes |
| 9HYPO | Ophiocordyceps unilateralis | Ascomycetes |
| OPHP1 | Ophiostoma piceae | Ascomycetes |
| PACTA | Pachysolen tannophilus | Ascomycetes |
| PARBD | Paracoccidioides brasiliensis | Ascomycetes |
| PARBA | Paracoccidioides lutzii | Ascomycetes |
| PENCA | Penicillium camembertii | Ascomycetes |
| PEND2 | Penicillium digitatum | Ascomycetes |
| PENEN | Penicillium expansum | Ascomycetes |
| PENIT | Penicillium italicum | Ascomycetes |
| PENNA | Penicillium nalgiovense | Ascomycetes |
| PENO1 | Penicillium oxalicum | Ascomycetes |
| PENPA | Penicillium patulum | Ascomycetes |
| PENRF | Penicillium roqueforti | Ascomycetes |
| PENRW | Penicillium rubens | Ascomycetes |
| PHANO | Phaeosphaeria nodorum | Ascomycetes |
| 9HELO | Phialocephala scopiformis | Ascomycetes |
| PICKU | Pichia kudriavzevii | Ascomycetes |
| PICSO | Pichia sorbitophila | Ascomycetes |
| PNEJI | Pneumocystis jirovecii | Ascomycetes |
| PNEMU | Pneumocystis murina | Ascomycetes |
| PODAN | Podospora anserina | Ascomycetes |
| PRIPA | Pristionchus pacificus | Ascomycetes |
| PSEFD | Pseudocercospora fijiensis | Ascomycetes |
| PSED2 | Pseudogymnoascus destructans | Ascomycetes |
| PYRTT | Pyrenophora teres | Ascomycetes |
| PYRTR | Pyrenophora tritici-repentis | Ascomycetes |
| PYROM | Pyronema omphalodes | Ascomycetes |
| ROSNE | Rosellinia necatrix | Ascomycetes |
| SACAR | Saccharomyces arboricola | Ascomycetes |
| YEAST | Saccharomyces cerevisiae | Ascomycetes |
| SACK1 | Saccharomyces kudriavzevii | Ascomycetes |
| PICST | Scheffersomyces stipitis | Ascomycetes |
| SCHCR | Schizosaccharomyces cryophilus | Ascomycetes |
| SCHJY | Schizosaccharomyces japonicus | Ascomycetes |
| SCHPO | Schizosaccharomyces pombe | Ascomycetes |
| SCLS1 | Sclerotinia sclerotiorum | Ascomycetes |
| SETT2 | Setosphaeria turcica | Ascomycetes |
| SORMK | Sordaria macrospora | Ascomycetes |
| SPAPN | Spathaspora passalidarum | Ascomycetes |
| SPHMS | Sphaerulina musiva | Ascomycetes |
| SPOS1 | Sporothrix schenckii | Ascomycetes |
| STACH | Stachybotrys chartarum | Ascomycetes |
| 9PLEO | Stemphylium lycopersici | Ascomycetes |
| 9ASCO | Sugiyamaella lignohabitans | Ascomycetes |
| TALEM | Talaromyces emersonii | Ascomycetes |
| TALIS | Talaromyces islandicus | Ascomycetes |
| TALMA | Talaromyces marneffei | Ascomycetes |
| TALSN | Talaromyces stipitatus | Ascomycetes |
| TAPDE | Taphrina deformans | Ascomycetes |
| TETBL | Tetrapisispora blattae | Ascomycetes |
| TETPH | Tetrapisispora phaffii | Ascomycetes |
| THITE | Thielavia terrestris | Ascomycetes |
| TOGMI | Togninia minima | Ascomycetes |
| TORDC | Torulaspora delbrueckii | Ascomycetes |
| TRIHA | Trichoderma harzianum | Ascomycetes |
| TRIEC | Trichophyton equinum | Ascomycetes |
| TRIRC | Trichophyton rubrum | Ascomycetes |
| TUBMM | Tuber melanosporum | Ascomycetes |
| UNCRE | Uncinocarpus reesii | Ascomycetes |
| UNCNE | Uncinula necator | Ascomycetes |
| VANPO | Vanderwaltozyma polyspora | Ascomycetes |
| VERA1 | Verticillium alfalfae | Ascomycetes |
| VERDV | Verticillium dahliae | Ascomycetes |
| WICAO | Wickerhamomyces anomalus | Ascomycetes |
| WICCF | Wickerhamomyces ciferrii | Ascomycetes |
| YARLI | Yarrowia lipolytica | Ascomycetes |
| ZYGB2 | Zygosaccharomyces bailii | Ascomycetes |
| ZYGRC | Zygosaccharomyces rouxii | Ascomycetes |
| ZYMTI | Zymoseptoria tritici | Ascomycetes |
| AGABU | Agaricus bisporus | Basidomycetes |
| AMAMU | Amanita muscaria | Basidomycetes |
| CERS8 | Ceriporiopsis subvermispora | Basidomycetes |
| CONPW | Coniophora puteana | Basidomycetes |
| COPC7 | Coprinopsis cinerea | Basidomycetes |
| CRYGR | Cryptococcus gattii | Basidomycetes |
| CRYNJ | Cryptococcus neoformans | Basidomycetes |
| DACPD | Dacryopinax primogenitus | Basidomycetes |
| 9APHY | Daedalea quercina | Basidomycetes |
| DICSQ | Dichomitus squalens | Basidomycetes |
| EXIGL | Exidia glandulosa | Basidomycetes |
| FOMPI | Fomitopsis pinicola | Basidomycetes |
| 9AGAR | Galerina marginata | Basidomycetes |
| GLOTA | Gloeophyllum trabeum | Basidomycetes |
| GRIFR | Grifola frondosa | Basidomycetes |
| HEBCY | Hebeloma cylindrosporum | Basidomycetes |
| HYPMA | Hypsizygus marmoreus | Basidomycetes |
| KALBG | Kalmanozyma brasiliensis | Basidomycetes |
| 9TREE | Kwoniella heveanensis | Basidomycetes |
| LACBS | Laccaria bicolor | Basidomycetes |
| LENED | Lentinula edodes | Basidomycetes |
| MALGO | Malassezia globosa | Basidomycetes |
| MALS4 | Malassezia sympodialis | Basidomycetes |
| MELLP | Melampsora larici-populina | Basidomycetes |
| USTV1 | Microbotryum lychnidis-dioicae | Basidomycetes |
| MIXOS | Mixia osmundae | Basidomycetes |
| MONRO | Moniliophthora roreri | Basidomycetes |
| PAXIN | Paxillus involutus | Basidomycetes |
| PHACS | Phanerochaete carnosa | Basidomycetes |
| PHLGI | Phlebiopsis gigantea | Basidomycetes |
| PISTI | Pisolithus tinctorius | Basidomycetes |
| PLEOS | Pleurotus ostreatus | Basidomycetes |
| POSPM | Postia placenta | Basidomycetes |
| PSEA2 | Pseudozyma antarctica | Basidomycetes |
| PSEHS | Pseudozyma hubeiensis | Basidomycetes |
| PUCGT | Puccinia graminis | Basidomycetes |
| 9BASI | Puccinia sorghi | Basidomycetes |
| PUCT1 | Puccinia triticina | Basidomycetes |
| PUNST | Punctularia strigosozonata | Basidomycetes |
| PYCCI | Pycnoporus cinnabarinus | Basidomycetes |
| 9HOMO | Rhizoctonia solani | Basidomycetes |
| RHOT1 | Rhodosporidium toruloides | Basidomycetes |
| RHOGW | Rhodotorula graminis | Basidomycetes |
| SCHCM | Schizophyllum commune | Basidomycetes |
| SERID | Serendipita indica | Basidomycetes |
| SERL3 | Serpula lacrymans | Basidomycetes |
| SPORE | Sporisorium reilianum | Basidomycetes |
| THACA | Thanatephorus cucumeris | Basidomycetes |
| TRAPU | Trametes pubescens | Basidomycetes |
| TRIAC | Trichosporon asahii var. asahii | Basidomycetes |
| USTH4 | Ustilago hordei | Basidomycetes |
| USTMA | Ustilago maydis | Basidomycetes |
| WALI9 | Wallemia ichthyophaga | Basidomycetes |
| WALMC | Wallemia mellicola | Basidomycetes |
| ALLMA | Allomyces macrogynus | Blastocladiomycota(fungi) |
| LINUN | Lingula unguis | Brachipoda |
| MONBE | Monosiga brevicollis | Choanoflagellida |
| SALR5 | Salpingoeca rosetta | Choanoflagellida |
| AILME | Ailuropoda melanoleuca | Chordata |
| ALLMI | Alligator mississippiensis | Chordata |
| ALLSI | Alligator sinensis | Chordata |
| AMAAE | Amazona aestiva | Chordata |
| ANAPL | Anas platyrhynchos | Chordata |
| ANOCA | Anolis carolinensis | Chordata |
| ANTCR | Antrostomus carolinensis | Chordata |
| APAVI | Apaloderma vittatum | Chordata |
| APTFO | Aptenodytes forsteri | Chordata |
| ASTMX | Astyanax mexicanus | Chordata |
| BALRE | Balearica regulorum | Chordata |
| BOVIN | Bos taurus | Chordata |
| BRAFL | Branchiostoma floridae | Chordata |
| BUCRH | Buceros rhinoceros | Chordata |
| CALJA | Callithrix jacchus | Chordata |
| CALAN | Calypte anna | Chordata |
| CAMFR | Camelus ferus | Chordata |
| CANLF | Canis lupus familiaris | Chordata |
| CARIC | Cariama cristata | Chordata |
| CAVPO | Cavia porcellus | Chordata |
| CHAPE | Chaetura pelagica | Chordata |
| CHAVO | Charadrius vociferus | Chordata |
| CHEMY | Chelonia mydas | Chordata |
| CHLSB | Chlorocebus sabaeus | Chordata |
| CIOIN | Ciona intestinalis | Chordata |
| CIOSA | Ciona savignyi | Chordata |
| COLST | Colius striatus | Chordata |
| COLLI | Columba livia | Chordata |
| CORBR | Corvus brachyrhynchos | Chordata |
| CRIGR | Cricetulus griseus | Chordata |
| DANRE | Danio rerio | Chordata |
| DIPOR | Dipodomys ordii | Chordata |
| HORSE | Equus caballus | Chordata |
| ERIEU | Erinaceus europaeus | Chordata |
| EURHL | Eurypyga helias | Chordata |
| FELCA | Felis catus | Chordata |
| FICAL | Ficedula albicollis | Chordata |
| FUKDA | Fukomys damarensis | Chordata |
| FULGA | Fulmarus glacialis | Chordata |
| CHICK | Gallus gallus | Chordata |
| GASAC | Gasterosteus aculeatus | Chordata |
| GAVST | Gavia stellata | Chordata |
| GORGO | Gorilla gorilla | Chordata |
| HALAL | Haliaeetus albicilla | Chordata |
| HETGA | Heterocephalus glaber | Chordata |
| HUMAN | Homo sapiens | Chordata |
| ICTTR | Ictidomys tridecemlineatus | Chordata |
| LARCR | Larimichthys crocea | Chordata |
| LATCH | Latimeria chalumnae | Chordata |
| LEPOC | Lepisosteus oculatus | Chordata |
| LEPDC | Leptosomus discolor | Chordata |
| LOXAF | Loxodonta africana | Chordata |
| MACFA | Macaca fascicularis | Chordata |
| MACMU | Macaca mulatta | Chordata |
| 9PASS | Manacus vitellinus | Chordata |
| MELGA | Meleagris gallopavo | Chordata |
| MERNU | Merops nubicus | Chordata |
| 9GRUI | Mesitornis unicolor | Chordata |
| MESAU | Mesocricetus auratus | Chordata |
| MONDO | Monodelphis domestica | Chordata |
| MOUSE | Mus musculus | Chordata |
| MUSPF | Mustela putorius furo | Chordata |
| MYOBR | Myotis brandtii | Chordata |
| MYODS | Myotis davidii | Chordata |
| MYOLU | Myotis lucifugus | Chordata |
| NEOLE | Neotoma lepida | Chordata |
| NESNO | Nestor notabilis | Chordata |
| NIPNI | Nipponia nippon | Chordata |
| NOMLE | Nomascus leucogenys | Chordata |
| OIKDI | Oikopleura dioica | Chordata |
| OPHHA | Ophiophagus hannah | Chordata |
| OPIHO | Opisthocomus hoazin | Chordata |
| ORENI | Oreochromis niloticus | Chordata |
| ORNAN | Ornithorhynchus anatinus | Chordata |
| RABIT | Oryctolagus cuniculus | Chordata |
| ORYLA | Oryzias latipes | Chordata |
| OTOGA | Otolemur garnettii | Chordata |
| SHEEP | Ovis aries | Chordata |
| PANTR | Pan troglodytes | Chordata |
| PAPAN | Papio anubis | Chordata |
| PATFA | Patagioenas fasciata | Chordata |
| PELSI | Pelodiscus sinensis | Chordata |
| PHALP | Phaethon lepturus | Chordata |
| PHACA | Phalacrocorax carbo | Chordata |
| PHORB | Phoenicopterus ruber | Chordata |
| PICPB | Picoides pubescens | Chordata |
| 9AVES | Podiceps cristatus | Chordata |
| POEFO | Poecilia formosa | Chordata |
| PONAB | Pongo abelii | Chordata |
| PTEAL | Pteropus alecto | Chordata |
| RAT | Rattus norvegicus | Chordata |
| SALSA | Salmo salar | Chordata |
| SARHA | Sarcophilus harrisii | Chordata |
| 9TELE | Scleropages formosus | Chordata |
| STRCA | Struthio camelus | Chordata |
| PIG | Sus scrofa | Chordata |
| TAEGU | Taeniopygia guttata | Chordata |
| TAKRU | Takifugu rubripes | Chordata |
| TARSY | Tarsius syrichta | Chordata |
| TAUER | Tauraco erythrolophus | Chordata |
| TETNG | Tetraodon nigroviridis | Chordata |
| TINGU | Tinamus guttatus | Chordata |
| TUPCH | Tupaia chinensis | Chordata |
| TYTAL | Tyto alba | Chordata |
| XENLA | Xenopus laevis | Chordata |
| XENTR | Xenopus tropicalis | Chordata |
| XIPMA | Xiphophorus maculatus | Chordata |
| BATDJ | Batrachochytrium dendrobatidis | Chytridiomycota |
| GONPR | Gonapodya prolifera | Chytridiomycota |
| SPIPN | Spizellomyces punctatus | Chytridiomycota |
| NEMVE | Nematostella vectensis | Cnidaria |
| THEKT | Thelohanellus kitauei | Cnidaria |
| GUITH | Guillardia theta | Cryptophyta |
| 9METZ | Lampea lactea | Ctenophora |
| RAMVA | Ramazzottius varieornatus | Ecdysozoa |
| STRPU | Strongylocentrotus purpuratus | Echinodermata |
| 9EUKA | Chrysochromulina sp. | Haptophyceae |
| EMIHU | Emiliania huxleyi | Haptophyceae |
| NAEGR | Naegleria gruberi | Heterolobosea |
| CAPO3 | Capsaspora owczarzaki | Ichthyosporea |
| 9EUGL | Bodo saltans | Euglenozoa |
| 9TRYP | Crithidia sp | Euglenozoa |
| LEIBR | Leishmania braziliensis | Euglenozoa |
| LEIMA | Leishmania major | Euglenozoa |
| TRYB2 | Trypanosoma brucei | Euglenozoa |
| TRYCC | Trypanosoma cruzi | Euglenozoa |
| TRYRA | Trypanosoma rangeli | Euglenozoa |
| ENCCU | Encephalitozoon cuniculi | Microsporidia |
| NEMP3 | Nematocida parisii | Microsporidia |
| SPRLO | Spraguea lophii | Microsporidia |
| TRAHO | Trachipleistophora hominis | Microsporidia |
| BIOGL | Biomphalaria glabrata | Mollusca |
| CRAGI | Crassostrea gigas | Mollusca |
| LOTGI | Lottia gigantea | Mollusca |
| OCTBM | Octopus bimaculoides | Mollusca |
| ABSGL | Absidia glauca | Mucoromycota |
| 9FUNG | Mortierella verticillata | Mucoromycota |
| MUCCL | Mucor circinelloides | Mucoromycota |
| PHYB8 | Phycomyces blakesleeanus | Mucoromycota |
| RHIID | Rhizophagus irregularis | Mucoromycota |
| RHIO9 | Rhizopus delemar | Mucoromycota |
| 9BILA | Ancylostoma ceylanicum | Nematoda |
| ANGCA | Angiostrongylus cantonensis | Nematoda |
| ANGCS | Angiostrongylus costaricensis | Nematoda |
| ANISI | Anisakis simplex | Nematoda |
| ASCLU | Ascaris lumbricoides | Nematoda |
| ASCSU | Ascaris suum | Nematoda |
| BRUMA | Brugia malayi | Nematoda |
| BRUPA | Brugia pahangi | Nematoda |
| BURXY | Bursaphelenchus xylophilus | Nematoda |
| CAEBE | Caenorhabditis brenneri | Nematoda |
| CAEBR | Caenorhabditis briggsae | Nematoda |
| CAEEL | Caenorhabditis elegans | Nematoda |
| CAEJA | Caenorhabditis japonica | Nematoda |
| CAERE | Caenorhabditis remanei | Nematoda |
| DICVI | Dictyocaulus viviparus | Nematoda |
| DRAME | Dracunculus medinensis | Nematoda |
| ENTVE | Enterobius vermicularis | Nematoda |
| GLOPA | Globodera pallida | Nematoda |
| HAECO | Haemonchus contortus | Nematoda |
| HAEPC | Haemonchus placei | Nematoda |
| HELBK | Heligmosomoides polygyrus bakeri | Nematoda |
| HETBA | Heterorhabditis bacteriophora | Nematoda |
| LOALO | Loa loa | Nematoda |
| MELHA | Meloidogyne hapla | Nematoda |
| NECAM | Necator americanus | Nematoda |
| NIPBR | Nippostrongylus brasiliensis | Nematoda |
| OESDE | Oesophagostomum dentatum | Nematoda |
| ONCOC | Onchocerca ochengi | Nematoda |
| ONCVO | Onchocerca volvulus | Nematoda |
| PARTI | Parastrongyloides trichosuri | Nematoda |
| STREA | Strongyloides papillosus | Nematoda |
| STRRB | Strongyloides ratti | Nematoda |
| STRER | Strongyloides stercoralis | Nematoda |
| THECL | Thelazia callipaeda | Nematoda |
| TOXCA | Toxocara canis | Nematoda |
| TRIBR | Trichinella britovi | Nematoda |
| TRIPS | Trichinella pseudospiralis | Nematoda |
| TRISP | Trichinella spiralis | Nematoda |
| TRIMR | Trichuris muris | Nematoda |
| TRITR | Trichuris trichiura | Nematoda |
| WUCBA | Wuchereria bancrofti | Nematoda |
| TRIAD | Trichoplax adhaerens | Placozoa |
| CLOSI | Clonorchis sinensis | Platyhelminthese |
| 9PLAT | Dendrocoelum lacteum | Platyhelminthese |
| ECHGR | Echinococcus granulosus | Platyhelminthese |
| ECHMU | Echinococcus multilocularis | Platyhelminthese |
| HYMDI | Hymenolepis diminuta | Platyhelminthese |
| HYMMI | Hymenolepis microstoma | Platyhelminthese |
| HYMNN | Hymenolepis nana | Platyhelminthese |
| 9CEST | Mesocestoides corti | Platyhelminthese |
| 9TREM | Opisthorchis viverrini | Platyhelminthese |
| SCHSO | Schistocephalus solidus | Platyhelminthese |
| SCHHA | Schistosoma haematobium | Platyhelminthese |
| SCHMA | Schistosoma mansoni | Platyhelminthese |
| TAEAS | Taenia asiatica | Platyhelminthese |
| TRIRE | Trichobilharzia regenti | Platyhelminthese |
| AMPQE | Amphimedon queenslandica | Porifera |
| PLABS | Plasmodiophora brassicae | Rhizaria |
| RETFI | Reticulomyxa filosa | Rhizaria |
| GALSU | Galdieria sulphuraria | Rhodophyta |
| 9STRA | Albugo candida | Stramenopiles |
| AURAN | Aureococcus anophagefferens | Stramenopiles |
| BLAHN | Blastocystis sp. subtype 1 | Stramenopiles |
| ECTSI | Ectocarpus siliculosus | Stramenopiles |
| HYAAE | Hyaloperonospora arabidopsidis | Stramenopiles |
| PHATC | Phaeodactylum tricornutum | Stramenopiles |
| PHYIT | Phytophthora infestans | Stramenopiles |
| PHYNI | Phytophthora nicotianae | Stramenopiles |
| PHYPR | Phytophthora parasitica | Stramenopiles |
| PHYSP | Phytophthora sojae | Stramenopiles |
| PYTUL | Pythium ultimum | Stramenopiles |
| SAPPC | Saprolegnia parasitica | Stramenopiles |
| THAOC | Thalassiosira oceanica | Stramenopiles |
| THAPS | Thalassiosira pseudonana | Stramenopiles |
| HYPDU | Hypsibius dujardini | Tardigrada |
| AMBTC | Amborella trichopoda | Viridiplantae |
| ANACO | Ananas comosus | Viridiplantae |
| ARALL | Arabidopsis lyrata | Viridiplantae |
| ARATH | Arabidopsis thaliana | Viridiplantae |
| ARAAL | Arabis alpina | Viridiplantae |
| BETVU | Beta vulgaris | Viridiplantae |
| BRADI | Brachypodium distachyon | Viridiplantae |
| BRANA | Brassica napus | Viridiplantae |
| BRAOL | Brassica oleracea | Viridiplantae |
| BRARP | Brassica rapa | Viridiplantae |
| CAJCA | Cajanus cajan | Viridiplantae |
| 9BRAS | Capsella rubella | Viridiplantae |
| CAPAN | Capsicum annuum | Viridiplantae |
| CEPFO | Cephalotus follicularis | Viridiplantae |
| CHLVA | Chlorella variabilis | Viridiplantae |
| CICAR | Cicer arietinum | Viridiplantae |
| 9ROSI | Citrus clementina | Viridiplantae |
| COCSC | Coccomyxa subellipsoidea | Viridiplantae |
| CUCME | Cucumis melo | Viridiplantae |
| DAUCA | Daucus carota | Viridiplantae |
| ERYGU | Erythranthe guttata | Viridiplantae |
| EUCGR | Eucalyptus grandis | Viridiplantae |
| EUTSA | Eutrema salsugineum | Viridiplantae |
| FUSOX | Fusarium oxysporum | Viridiplantae |
| 9LAMI | Genlisea aurea | Viridiplantae |
| SOYBN | Glycine max | Viridiplantae |
| GLYSO | Glycine soja | Viridiplantae |
| GONPE | Gonium pectorale | Viridiplantae |
| GOSAR | Gossypium arboreum | Viridiplantae |
| GOSHI | Gossypium hirsutum | Viridiplantae |
| GOSRA | Gossypium raimondii | Viridiplantae |
| HORVV | Hordeum vulgare | Viridiplantae |
| JATCU | Jatropha curcas | Viridiplantae |
| KLEFL | Klebsormidium flaccidum | Viridiplantae |
| LUPAN | Lupinus angustifolius | Viridiplantae |
| MARPO | Marchantia polymorpha | Viridiplantae |
| MEDTR | Medicago truncatula | Viridiplantae |
| MICCC | Micromonas commoda | Viridiplantae |
| 9CHLO | Monoraphidium neglectum | Viridiplantae |
| 9ROSA | Morus notabilis | Viridiplantae |
| MUSAM | Musa acuminata | Viridiplantae |
| NELNU | Nelumbo nucifera | Viridiplantae |
| NICAT | Nicotiana attenuata | Viridiplantae |
| NICSY | Nicotiana sylvestris | Viridiplantae |
| TOBAC | Nicotiana tabacum | Viridiplantae |
| ORYBR | Oryza brachyantha | Viridiplantae |
| ORYGL | Oryza glaberrima | Viridiplantae |
| 9ORYZ | Oryza meridionalis | Viridiplantae |
| ORYNI | Oryza nivara | Viridiplantae |
| ORYPU | Oryza punctata | Viridiplantae |
| ORYSI | Oryza sativa | Viridiplantae |
| OSTLU | Ostreococcus lucimarinus | Viridiplantae |
| OSTTA | Ostreococcus tauri | Viridiplantae |
| PHAAN | Phaseolus angularis | Viridiplantae |
| PHAVU | Phaseolus vulgaris | Viridiplantae |
| PHYPA | Physcomitrella patens | Viridiplantae |
| POPTR | Populus trichocarpa | Viridiplantae |
| PRUPE | Prunus persica | Viridiplantae |
| RICCO | Ricinus communis | Viridiplantae |
| SELML | Selaginella moellendorffii | Viridiplantae |
| SETIT | Setaria italica | Viridiplantae |
| SOLLC | Solanum lycopersicum | Viridiplantae |
| SOLTU | Solanum tuberosum | Viridiplantae |
| SORBI | Sorghum bicolor | Viridiplantae |
| SPIOL | Spinacia oleracea | Viridiplantae |
| THECC | Theobroma cacao | Viridiplantae |
| WHEAT | Triticum aestivum | Viridiplantae |
| TRIUA | Triticum urartu | Viridiplantae |
| VIGRR | Vigna radiata | Viridiplantae |
| VITVI | Vitis vinifera | Viridiplantae |
| VOLCA | Volvox carteri | Viridiplantae |
| MAIZE | Zea mays | Viridiplantae |
| ZOSMR | Zostera marina | Viridiplantae |
| CONC2 | Conidiobolus coronatus | Zoopagomycota |
| ZANCU | Zancudomyces culisetae | Zoopagomycota |

Sample Codeml.ctl file

seqfile = nup133_c_nucl_aligned.fas

treefile = outtree

outfile = mlc

noisy = 4

verbose = 1

runmode = 0

seqtype = 1

CodonFreq = 2

estFreq = 0

ndata = 1

clock = 0

aaDist = 0

model = 0

NSsites = 2

icode = 0

Mgene = 0

fix_kappa = 0

kappa = 2

fix_omega = 0

omega = 0.4

fix_alpha = 1

alpha = 0

Malpha = 0

ncatG = 5

getSE = 1

RateAncestor = 0

Small_Diff = 5e-7

cleandata = 0

fix_blength = 0

method = 0

**References**

1. Pei, J., Kim, B. H., & Grishin, N. V. (2008). PROMALS3D: A tool for multiple protein sequence and structure alignments. *Nucleic Acids Research*, *36*(7), 2295–2300. doi:10.1093/nar/gkn072

2. Waterhouse, A. M., Procter, J. B., Martin, D. M. A., Clamp, M., & Barton, G. J. (2009). Jalview Version 2-A multiple sequence alignment editor and analysis workbench. *Bioinformatics*, *25*(9), 1189–1191. doi:10.1093/bioinformatics/btp033

3. Drozdetskiy, A., Cole, C., Procter, J., & Barton, G. J. (2015). JPred4: A protein secondary structure prediction server. *Nucleic Acids Research*, *43*(W1), W389–W394. doi:10.1093/nar/gkv332

4. Finn, R. D., Clements, J., Arndt, W., Miller, B. L., Wheeler, T. J., Schreiber, F., Bateman, A., & Eddy, S. R. (2015). HMMER web server: 2015 Update. *Nucleic Acids Research*, *43*(W1), W30–W38. doi:10.1093/nar/gkv397

5. Liu, W. hong, Xie, Y., Ma, J., Luo, X., Nie, P., Zuo, Z., Lahrmann, U., Zhao, Q., Zheng, Y., Zhao, Y., Xue, Y., & Ren, J. (2015). IBS: An illustrator for the presentation and visualization of biological sequences. *Bioinformatics*, *31*(20), 3359–3361. doi:10.1093/bioinformatics/btv362

6. Yang, Z. (2017). PAML 4 : Phylogenetic Analysis by Maximum Likelihood, (December), 1586–1591. doi:10.1093/molbev/msm088
